# Multivariate Analysis of Data From Crossover Clinical Trials

**DOI:** 10.1002/bimj.70193

**Published:** 2026-10-11

**Authors:** Yi Jiang, Vernon M. Chinchilli

**Affiliations:** ^1^ Department of Public Health Sciences, College of Medicine The Pennsylvania State University Hershey Pennsylvania USA

**Keywords:** crossover trials, intra‐subject variability, linear mixed‐effects model, multivariate analysis

## Abstract

In crossover designs, multiple outcomes are often collected to assess treatment effectiveness in a comprehensive manner. Also, interest has grown in comparing intra‐subject variability between treatments in such designs. We present three distinct multivariate linear mixed‐effects models, with the choice of the model depending on the design and assumptions regarding carryover effects. We discuss maximum likelihood (ML) and restricted ML (REML) approaches for parameter estimation. For a special case that a crossover design is uniform within sequences, we derive the closed‐form expressions for the ML and REML estimators of the variance components. We propose general hypothesis testing methods for the treatment effects and the intra‐subject variance matrices. The latter situation has not received much attention in the statistical literature. For the special case, we derive the exact test statistics for treatment means comparisons and for testing the null hypothesis of homogeneous intra‐subject variance matrices. Simulation studies are conducted to validate the superior performance of the proposed multivariate approach compared with the usual univariate approach in testing treatment effects. Finally, we apply the methodologies to two data examples.

## 1 Introduction

Crossover designs are repeated measurements designs in which participants (or subjects) are randomized to different sequences of interventions with the objective to study the difference between these interventions (reviewed in details in Senn 2002; Jones and Kenward 2003; Vonesh and Chinchilli 1996). Unlike subjects in the conventional parallel designs who are randomized to receive only one intervention during the whole trial, subjects in crossover designs are randomized to receive two or more interventions sequentially, that is, the patients cross over from one intervention to another during the course of the trial. In this manner, crossover designs allow each participant to serve as his/her own control (Jones and Kenward 2003). This feature yields an advantage for crossover designs relative to parallel designs—the variance of the estimated treatment comparisons usually is reduced. Because of the high efficiency of comparisons, crossover designs are widely used in medicine, agriculture, and education. In the field of clinical trials, crossover designs often are considered for those trials studying chronic and relatively stable diseases, for example, asthma or diabetes. A review of all 519 PubMed‐indexed randomized trials published in December, 2000 showed that nearly one‐fourth (22%) of them used crossover design (Chan and Altman 2005). Despite the saving in resources and the wide use, the design and analysis of crossover trials require special attention (Wellek and Blettner 2012). That is because nuisance effects (sequence effects, period effects, and carryover effects) may be aliased with direct treatment effects, which may bias the interpretation of the data analysis. Recent guidance, including the CONSORT 2010 extension for randomized crossover trials (Dwan et al. 2019), further emphasizes the need for transparent and standardized reporting practices.

In randomized clinical trials, multiple outcomes are increasingly collected to assess treatment effectiveness and to explore the relationships among the outcomes. The decision to include several outcomes stems from various factors, such as the absence of agreement on the most crucial clinical outcomes or the intention to show effectiveness across multiple outcomes (Teixeira‐Pinto et al. 2009). Although many clinical trials analyze the multiple outcomes as a single primary outcome with several secondary outcomes, for numerous diseases and disorders the health status of a patient cannot be sufficiently quantified using only one primary outcome (Vickerstaff et al. 2021). Examples include chronic obstructive pulmonary disease (COPD) (Agustí and Vestbo 2011), mental health disorders (Tyler et al. 2011; De Los Reyes et al. 2011), and stroke (Mayo and Scott 2011). A straightforward method for analyzing multiple outcomes is to evaluate each one individually, but this method fails to consider the pairwise correlations among the outcomes. Another method frequently used in clinical trials is to combine the multiple outcomes into a composite outcome. However, such measures cause some loss of information and difficulty in interpretation. Composite endpoints can exaggerate the perceived benefits of a treatment if the more frequent but less critical events dominate the composite measure (Freemantle et al. 2003). Multivariate analysis, however, offers advantages over these methods. It assesses the effects of an intervention on multiple outcomes simultaneously. Outcomes in clinical trials are often interrelated. By accounting for the correlations among different outcomes, multivariate analysis provides a more detailed picture of how an intervention affects each outcome individually and jointly. One main objective in our research is to develop a methodology that is able to accommodate multivariate outcomes in crossover trials.

Another important goal of our research is to develop a multivariate model that allows the estimations and comparisons of the intra‐subject variance matrices of treatments within the framework of crossover designs. In certain circumstances, for instance, bioequivalence trials, where test drugs and the corresponding reference drugs are compared to determine if they yield comparable blood concentration × time profiles (Midha and McKay 2009), investigators may be concerned that the test formulation displays greater variability within subjects compared to the reference formulation (Chinchilli and Esinhart 1996). The difference in within‐subject variability may suggest differences in drug formulation and/or administration (Guilbaud 1993). Larger within‐subject variance of the test drug may imply that it cannot provide the same predictable therapeutic effect as the reference drug, potentially leading to inferior efficacy and safety.

There are some previous publications on using multivariate methods to analyze data from crossover trials. Many of them focused on analyzing crossover trials in which repeated measures are obtained within each period but only with a single outcome variable, which is different from the multivariate problem in which we are interested. For example, Patel and Hearne III (1980) introduced a multivariate linear model and developed corresponding tests to deal with a repeated measures 2×2 crossover design. Putt and Chinchilli (1999) developed a generalized framework with a mixed‐effects model which allows both the between‐ and within‐subject variance to differ among treatments. Although the research question is not exactly the same, these articles provide some framework for analyzing crossover trials with multivariate outcomes. Additionally, there are publications that have developed specific methods for multivariate outcome crossover trials. Rodriguez‐Carvajal (1999) adapted the methods proposed by Patel and Hearne III (1980) for the analysis of the multivariate structured data from 2×2 crossover trials. Grender and Johnson (1993, 1992) proposed a general multivariate linear model and discussed testing the multivariate general linear hypotheses. Their methods can be generalized to more complex crossover designs than the simplest 2×2 design. However, the aforementioned multivariate linear models do not consider two sources of variation (within‐ and between‐subject) separately, and the use of PROC GLM in SAS for multivariate analysis excludes a subject if any of his/her measurements are missing. Johnson and Grender (1993), Johnson and Mercante (1996) addressed the application of nonparametric methods (multivariate rank tests and permutation tests) to the analysis of data arising in 2×2 crossover designs with a multivariate response. Farrow and Goldstein (1993) outlined a general approach based on Bayes linear methods to the analysis of mean effects for grouped multivariate repeated measurements studies. Liu and Li (2016) invoked a Bayesian joint model to analyze three visual analog scale responses (three non‐Gaussian variables) and a time‐to‐event response jointly. Mwangi et al. (2024) developed a model to study the associations among six outcomes from a 2×2 crossover trial. The flexible piecewise joint models allow for six outcomes to depend on separate random effects, which themselves are correlated. Although the above studies can solve the problem of multivariate analysis for crossover trials to some extent, they do not allow for heterogeneous intra‐subject variance matrices for different treatments. Plus, many of the approaches are developed within the framework of 2×2 crossover trials, and it is not always possible to extend them to more complicated crossover designs. For these reasons, we wish to provide a more general approach that enables multivariate analysis in any crossover trial, while also allowing for statistical inference with respect to intra‐subject variance matrices of the treatments.

Linear mixed‐effects models (LMMs) are widely used in analyzing studies with repeated measurements on each subject. The correlations among the repeated measurements are captured by the random effects and their distributional assumption. Thus, LMMs also are suitable for analyzing data from crossover designs (Jones and Kenward 2003; Zhou 2012). Chinchilli and Esinhart (1996) developed statistical models and hypothesis tests for crossover trials with a single outcome, which also enables the estimation and comparison of intra‐subject variances. The latter situation has not received much attention in the statistical literature. The statistical models they proposed are LMMs adjusted for nuisance location effects (sequence effects, period effects and their interactions) and potential carryover effects. The work we develop here is a multivariate extension to their method that conducts the analysis of multiple outcomes simultaneously.

The motivating data example in our study is from a crossover trial called the tiotropium bromide as an alternative to increased inhaled glucocorticoid in patients inadequately controlled on a lower dose of inhaled corticosteroid (TALC) study reported by Peters et al. (2010). This crossover clinical trial involved randomization of 210 adult patients into 6 treatment sequences. All participants received background therapy with beclomethasone 80 μg twice daily throughout the study, including washout periods. The three treatments compared, in addition to the background therapy, were: (A) tiotropium bromide at a dose of 18 μg every morning; (B) an additional dose of beclomethasone 80 μg twice daily (i.e., doubling the beclomethasone dose); and (C) salmeterol xinafoate at a dose of 50 μg twice daily. The patients were randomized to one of the six treatment sequences: ABC, ACB, BAC, BCA, CAB, and CBA. In this data example, the multivariate response variable we are interested in is the change of four lung function measurements from baseline: forced vital capacity (FVC), forced expiratory volume in 1 s (FEV1), peak expiratory flow rate (PEFR), and forced mid‐expiratory flow (FEF2575).

The remainder of this article is constructed as follows. In Section 2, We present three distinct multivariate linear mixed‐effects models, with the choice of the model depending on the design and assumptions regarding carryover effects. Each model includes the maximum allowable number of nuisance parameters (saturated model) and incorporates qt‐variate random subject effects, where t represents the number of treatments and q represents the number of outcome variables. We discuss both maximum likelihood (ML) and restricted maximum likelihood (REML) approaches for deriving parameter estimators. We show that a closed‐form solution exists for the ML and REML estimators of the vector of fixed parameters given the variance components, although the variance components usually are unknown. However, for the special case of a crossover design that is uniform within sequences, there are closed‐form expressions for the ML and REML estimators of the variance components. We propose general hypothesis testing methods for the treatment (and/or other covariates) effects and the intra‐subject variance matrices. Additionally, for the special case, we demonstrate that (i) the null hypothesis that a vector of treatment contrast means is zero reduces to a Hotelling's $T^{2}$ test, and (ii) an exact test based on likelihood ratio test exists for the null hypothesis of homogeneous intra‐subject matrices. In Section 3, we perform simulation studies based on the TALC dataset to validate the superior performance of the proposed multivariate approach compared with the usual univariate approach in testing treatment effects. In Section 4, we illustrate the methodologies by implementing our models on two data examples: the dataset from TALC study, and a hypothetical four‐period crossover trial dataset generated based on the TALC data. Finally, Section 5 concludes the article by discussing the strengths and limitations of the proposed methods, the directions for future research, and critical insights into the design considerations of crossover trials.

## 2 Methods

### 2.1 Definitions of Crossover Design Properties

Prior to demonstration of the statistical models, we first need to introduce some definitions of crossover designs. A cross‐over design is considered uniform within sequences if each treatment k, k=1,⋯,t, appears the same number of times ($p_{k}$) within each sequence. A cross‐over design is uniform within periods if each treatment appears the same number of times within each period across all sequences. If a design is uniform within both sequences and periods, it is simply referred to as uniform.

The (first‐order) carryover effect is defined as the lingering effect of a treatment administered in the immediately preceding period on the response measurement in the current period. A crossover design is balanced with respect to first‐order carryover effects if each treatment immediately precedes every other treatment the same number of times. It is strongly balanced with respect to first‐order carryover effects if each treatment immediately precedes every other treatment, including itself, the same number of times. A strongly balanced design can be constructed by repeating the last period in a balanced design.

Figure 1 presents a schematic illustration of a hypothetical two‐sequence, two‐treatment, two‐period crossover design (a 2×2 crossover design). This design is both uniform and balanced. In general, a crossover design may consist of various sets of treatment sequences, depending on the number of treatments, the sequence length, and the specific objectives of the trial. The chosen design determines which nuisance effects are not confounded with the treatment mean effects. Because sequence, period, and carryover effects can be aliased with direct treatment effects, the analysis of crossover trials requires special consideration (Chinchilli and Esinhart 1996).

**FIGURE 1:**
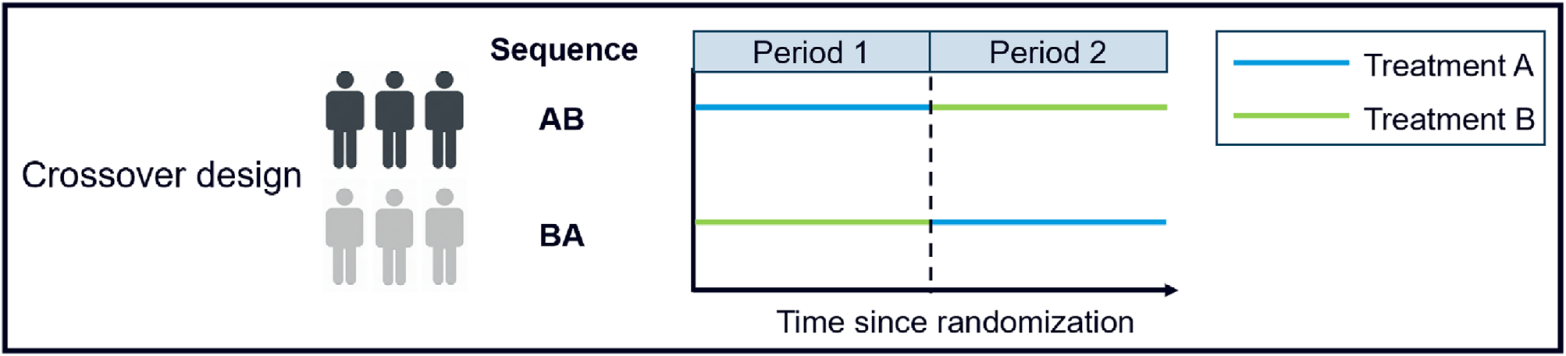
A schematic figure of a hypothetical two‐sequence, two‐treatment, two‐period crossover design (a 2×2 crossover design).

### 2.2 Multivariate Statistical Models

We consider a general s×p crossover design involving t treatments or interventions. The design comprises s distinct treatment sequences, each spanning p periods during which a multivariate response consisting of q outcome variables is recorded. It is assumed that the multivariate response is measured exactly once in each treatment period.

For our general s×p crossover design with t treatments, we assume that within sequence i, where i=1,2,…,s, there are $p_{ik}$ replications of treatment k, for k=1,2,…,t, such that

$$p_{i1}+p_{i2}+\cdots +p_{it}=p.$$

Although this condition is not strictly necessary, we assume for ease of presentation that each $p_{ik}\geq 1$. Furthermore, we assume that $n_{i}$ experimental units are randomized to sequence i, for i=1,2,…,s, such that

$$n_{1}+n_{2}+\cdots +n_{s}=n,$$

where n denotes the total sample size.

We consider saturated models in our study, which have the advantage of preserving a one‐to‐one correspondence between the sp cell means and the sp location parameters (Chinchilli and Esinhart 1996).

#### 2.2.1 Multivariate Model With no Carryover Effects

We develop a multivariate extension of the crossover design model originally proposed by Chinchilli and Esinhart (Chinchilli and Esinhart 1996).

(1) $$Y_{ijkl}=\mu_{k}+\left(I_{q\times q}⊗X_{ijkl}^{T}\right)\beta +\gamma_{ikl}+u_{ijk}+e_{ijkl}$$

where

- $Y_{ijkl}$ is a q×1 vector of outcome variables measured for the lth replication of the kth treatment for the jth participant within the ith sequence, $i=1,2,\text{…},s,\;j=1,2,\text{…},n_{i},k=1,2,\text{…},t,\;l=1,2,\text{…},p_{ik}$
- $\mu_{k}$ is a q×1 vector of population means for the kth treatment
- $X_{ijkl}$ is a c×1 vector of patient‐level baseline covariates
- $\beta =\left[\begin{array}{c} \beta_{1}\\ \beta_{2}\\ \vdots \\ \beta_{q} \end{array}\right]$ is a qc×1 vector of unknown fixed‐effects parameters
- $\gamma_{ikl}$ is a q×1 vector of unknown fixed‐effects nuisance parameters
- $u_{ijk}$ is a q×1 vector of random effects in which $u_{ij}=\left[\begin{array}{c} u_{ij1}\\ \begin{array}{c} u_{ij2}\\ \vdots \end{array}\\ u_{ijt} \end{array}\right]$ and the $u_{ij}$’s are mutually independent with $u_{ij}\;∼\;N_{qt}\left(0_{qt\times 1},\Omega \right)$, and $\Omega =\left[\begin{array}{ccc} \Omega_{11} & \Omega_{12} & \begin{array}{cc} \cdots & \Omega_{1t} \end{array}\\ \Omega_{21} & \Omega_{22} & \begin{array}{cc} \cdots & \Omega_{2t} \end{array}\\ \begin{array}{c} \vdots \\ \Omega_{t1} \end{array} & \begin{array}{c} \vdots \\ \Omega_{t2} \end{array} & \begin{array}{cc} \begin{array}{c} \ddots \\ \cdots \end{array} & \begin{array}{c} \vdots \\ \Omega_{tt} \end{array} \end{array} \end{array}\right]$ is a qt×qt positive definite matrix
- $e_{ijkl}$ is a q×1 vector of random error terms in which the $e_{ijkl}$’s are mutually independent with $e_{ijkl}\;∼\;N_{q}\left(0_{q\times 1},\Sigma_{k}\right)$, and $\Sigma_{k}$ is a q×q positive definite matrix
- the $u_{ij}$’s and the $e_{ijkl}$’s are mutually independent

For treatment k, where k=1,2,…,t, the covariance matrix $\Sigma_{k}$ is not estimable if $p_{ik}\leq 1$ for all i=1,2,…,s, since in this case $\Omega_{kk}+\Sigma_{k}$ is not separable.

In the statistical model described above, we assume that carryover effects are null. The nuisance parameters, $\gamma_{ikl}$, represent the sequence‐by‐period interactions nested within each treatment. Following (Chinchilli and Esinhart 1996), and to avoid over‐parameterization, we impose the following constraint on the nuisance parameters:

(2) $$\sum_{i=1}^{s}\sum_{l=1}^{p_{ik}}\gamma_{ikl}=0_{q\times 1},\;k=1,2,\text{…},t.$$

If the crossover design is balanced (or strongly balanced) with respect to first‐order carryover effects, the statistical model can be extended to incorporate a q×1 vector of common carryover effects, λ, or alternatively, q×1 vectors of treatment‐specific carryover effects, $\lambda_{k}$, for k=1,2,…,t. In the former case, a single additional constraint is imposed on the nuisance parameters, whereas in the latter case, t additional constraints are required, as detailed below.

#### 2.2.2 Multivariate Model With a Common Carryover Effect

The statistical model in Equation (1) can be modified to include a q×1 vector of common carryover effects, λ, with one additional constraint imposed on the nuisance parameters.

(3) $$Y_{ijkl}=\mu_{k}+\left(I_{q\times q}⊗X_{ijkl}^{T}\right)\beta +r_{ikl}\lambda +\gamma_{ikl}+u_{ijk}+e_{ijkl}$$

where $r_{ikl}$ is an indicator variable for carryover effect.

(4) $$r_{ikl}=\left\{\begin{array}{cc} 1 & \;\text{if replication}\;l\;\text{of treatment}\;k\;\text{in sequence}\;i\\ & \;\text{occurs after period 1}\\ 0 & \;\text{otherwise} \end{array}\right.$$

In model (3), there are s×p total cells, and the parameters to be estimated include $\mu_{k}$ for k=1,…,t, and the common carryover effect λ. This leaves sp−t−1 degrees of freedom for the nuisance parameters $\gamma_{ikl}$. Accordingly, we impose t+1 constraints on the nuisance parameters:

(5) $$\begin{array}{cc} & \sum_{i=1}^{s}\sum_{l=1}^{p_{ik}}\gamma_{ikl}=0_{q\times 1},\;k=1,2,\text{…},t\\ & \sum_{i,k,l\in J}\gamma_{ikl}=0_{q\times 1} \end{array}$$

where

$$\begin{array}{ccc} J & = & \{\left(i,k,l\right):r_{ikl}=1,\text{for each}\;i=1,2,\text{…},s,k=1,\text{…},t,\text{and}\;\\ l & = & 1,\text{…},p_{ik}\} \end{array}$$

#### 2.2.3 Multivariate Model With Treatment‐Specific Carryover Effects

If the crossover design is strongly balanced with respect to first‐order carryover effects, the statistical model can be modified to include q×1 vectors of treatment‐specific carryover effects, $\lambda_{k}$, for k=1,2,…,t. In this case, t additional constraints are imposed on the nuisance parameters.

(6) $$\begin{array}{ccc} Y_{ijkl} & = & \mu_{k}+\left(I_{q\times q}⊗X_{ijkl}^{T}\right)\beta +r_{ikl1}\lambda_{1}+\cdots +r_{iklt}\lambda_{t}\\ & & +\gamma_{ikl}+u_{ijk}+e_{ijkl} \end{array}$$

where for m=1,…,t,

(7) $$r_{iklm}=\left\{\begin{array}{cc} 1 & \;\text{if treatment}\;m\;\text{immediately precedes}\\ & \;\text{replicate}\;l\;\text{of treatment}\;k\;\text{in sequence}\;i\\ 0 & \;\text{otherwise} \end{array}\right.$$

 $\lambda_{1},\text{…},\lambda_{t}$ are q×1 vectors representing the carryover effects for treatments 1,…,t, respectively. In the model specified in Equation (6), the parameters to be estimated include $\mu_{k}$ and $\lambda_{k}$ for k=1,…,t. This leaves sp−2t degrees of freedom for the nuisance parameters $\gamma_{ikl}$. Accordingly, we impose 2t constraints on the nuisance parameters:

(8) $$\begin{array}{ccc} & \sum_{i=1}^{s}\sum_{l=1}^{p_{ik}}\gamma_{ikl}=0_{q\times 1}, & k=1,2,\text{…},t\\ & \sum_{i,k,l\in J_{m}}\gamma_{ikl}=0_{q\times 1}, & m=1,2,\text{…},t \end{array}$$

where

$$\begin{array}{ccc} J_{m} & = & \{\left(i,k,l\right):r_{iklm}=1,\text{for each}\;i=1,2,\text{…},s,k=1,\text{…},t,\text{and}\;\\ l & = & 1,\text{…},p_{ik}\} \end{array}$$

### 2.3 Estimations for the Mixed‐Effects Models

We denote the pq×1 vector of measurements for subject j in sequence i, where i=1,…,s and $j=1,\text{…},n_{i}$, as

(9) $$Y_{ij}=\text{vec}\left[Y_{ij11}\cdots Y_{ij1p_{i1}}\cdots Y_{ijt1}\cdots Y_{ijtp_{it}}\right]$$

The expectation of the vector $Y_{ij}$ depends on the choice of the statistical model. For example, under the model specified in Equation (1), the expectation is given by

(10) $$E\left[Y_{ij}\right]=\left[\begin{array}{c} \mu_{1}+\left(I_{q\times q}⊗X_{ij11}^{T}\right)\beta +\gamma_{i11}\\ \vdots \\ \mu_{1}+\left(I_{q\times q}⊗X_{ij1p_{i1}}^{T}\right)\beta +\gamma_{i1p_{i1}}\\ \vdots \\ \vdots \\ \mu_{t}+\left(I_{q\times q}⊗X_{ijt1}^{T}\right)\beta +\gamma_{it1}\\ \vdots \\ \mu_{t}+\left(I_{q\times q}⊗X_{ijtp_{it}}^{T}\right)\beta +\gamma_{itp_{it}} \end{array}\right]$$

The variance‐covariance matrix of the vector $Y_{ij}$ is

(11) $$\begin{array}{ccc} & & \text{var}(Y_{ij})\\ & & \;=\left[\begin{array}{cccc} & J_{p_{i1}\times p_{i1}}⊗\Omega_{11}+I_{p_{i1}\times p_{i1}}⊗\Sigma_{1} & \cdots & J_{p_{i1}\times p_{it}}⊗\Omega_{1t}\\ & \vdots & \ddots & \vdots \\ & J_{p_{it}\times p_{i1}}⊗\Omega_{t1} & \cdots & J_{p_{it}\times p_{it}}⊗\Omega_{tt}+I_{p_{it}\times p_{it}}⊗\Sigma_{t} \end{array}\right]=\Lambda_{i} \end{array}$$

In Equations (10) and (11), $I_{u\times u}$ denotes the u×u identity matrix, and $J_{u\times v}$ denotes the u×v matrix of unit values.

We combine the treatment effects $\mu_{k}$ (k=1,…,t), covariate effects β, and fixed‐effects location parameters $\gamma_{ikl}$ (along with either the common carryover effect λ or the treatment‐specific carryover effects $\lambda_{m}$, m=1,…,t) into a vector $\beta_{q(c+sp)\times 1}^{*}$. Here, qc is the dimension corresponding to the covariate coefficients in β, and qsp is the total dimension associated with the treatment effects and the fixed‐effects nuisance parameters.

Let $X_{ij}^{*}$ denote the corresponding design matrix for subject j in sequence i. Then, the distribution of the vector $Y_{ij}$ becomes

(12) $$Y_{ij}∼N_{qp}\left(X_{ij}^{*}\beta^{*},\Lambda_{i}\right)$$

#### 2.3.1 ML/REML Estimates

The log‐likelihood for data $Y_{11},\text{…},Y_{1n_{1}},\text{…}$, $Y_{s1},\text{…},Y_{sn_{s}}$ is

(13) $$\begin{array}{ccc} l & = & \text{const}-\frac{1}{2}\sum_{i=1}^{s}\sum_{j=1}^{n_{i}}\log\left|\Lambda_{i}\right|\\ & & -\frac{1}{2}\sum_{i=1}^{s}\sum_{j=1}^{n_{i}}{\left(Y_{ij}-X_{ij}^{*}\beta^{*}\right)}^{T}\Lambda_{i}^{-1}\left(Y_{ij}-X_{ij}^{*}\beta^{*}\right) \end{array}$$

Using the Fisher scoring algorithm, we iteratively update ${\hat{\beta }}^{*}|{\hat{\Lambda }}_{i}$ and ${\hat{\Lambda }}_{i}|{\hat{\beta }}^{*}$ until convergence (Jennrich and Schluchter 1986). The detailed derivation of the algorithm is in the Appendix A.1. The resultant ML and REML estimators of $\beta^{*}$ are

(14) $${\hat{\beta }}_{\mathrm{ML}}^{*}={\left(\sum_{i=1}^{s}\sum_{j=1}^{n_{i}}X_{ij}^{*T}{\hat{\Lambda }}_{i,\mathrm{ML}}^{-1}X_{ij}^{*}\right)}^{-1}\left(\sum_{i=1}^{s}\sum_{j=1}^{n_{i}}X_{ij}^{*T}{\hat{\Lambda }}_{i,\mathrm{ML}}^{-1}Y_{ij}\right)$$

(15) $${\hat{\beta }}_{\mathrm{REML}}^{*}={\left(\sum_{i=1}^{s}\sum_{j=1}^{n_{i}}X_{ij}^{*T}{\hat{\Lambda }}_{i,\mathrm{REML}}^{-1}X_{ij}^{*}\right)}^{-1}\left(\sum_{i=1}^{s}\sum_{j=1}^{n_{i}}X_{ij}^{*T}{\hat{\Lambda }}_{i,\mathrm{REML}}^{-1}Y_{ij}\right)$$

#### 2.3.2 Special Case With Closed‐Form Solutions

Consider a special case in which closed‐form solutions exist for the ML and REML estimators of the variance components. This situation arises when there are no baseline covariates in the model, the crossover design is uniform within sequences, and each of the t treatments has replication so that all variance components are estimable; that is, $p_{1k}=\cdots =p_{sk}\left(=p_{k}\geq 2\right)$ for k=1,2,…,t. In this case, the number of replications for treatment k is the same across all sequences, resulting in identical variance matrices: $\Lambda_{1}=\Lambda_{2}=\cdots =\Lambda_{s}$.

For treatment k within sequence i (i=1,2,…,s;k=1,2,…,t), we apply a full‐rank transformation to the set of $p_{k}$ measurements on experimental unit j ($j=1,2,\text{…},n_{i}$), from $Y_{ijk}=\text{vec}\left[Y_{ijk1},\text{…},Y_{ijkp_{k}}\right]$ to: (1) a q×1 average vector of these $p_{k}$ measurements, and (2) a vector of $\left(p_{k}-1\right)$ orthogonal contrasts (each is a q×1 vector), denoted respectively as

(16) $$\begin{array}{ccc} & & \left(1\right)\;{\bar{Y}}_{ijk\cdot }=\frac{1}{p_{k}}\sum_{l=1}^{p_{k}}Y_{ijkl}\;\text{and}\\ & & \left(2\right)\;Y_{ijk}^{*}=\text{vec}\left[Y_{ijk,1}^{*},Y_{ijk,2}^{*},\text{…},Y_{ijk,p_{k}-1}^{*}\right] \end{array}$$

Since the transformations are linear functions of the $Y_{ij}$’s, the transformed variables described above are normally distributed. The expectation for subject j within sequence i ($i=1,2,\text{…},s;\;j=1,2,\text{…},n_{i}$) is given by:

(17) $$\begin{array}{ccc} & & E\left[\begin{array}{c} {\bar{Y}}_{ij1\cdot }\\ {\bar{Y}}_{ij2\cdot }\\ \vdots \\ {\bar{Y}}_{ijt\cdot }\\ Y_{ij1}^{*}\\ \vdots \\ Y_{ijt}^{*} \end{array}\right]=\left[\begin{array}{c} \mu_{1}+{\bar{\gamma }}_{i1\cdot }\\ \mu_{2}+{\bar{\gamma }}_{i2\cdot }\\ \vdots \\ \mu_{t}+{\bar{\gamma }}_{it\cdot }\\ \nu_{i1}^{\left(1\right)}\\ \vdots \\ \nu_{it}^{\left(1\right)} \end{array}\right]\text{or}=\left[\begin{array}{c} \mu_{1}+{\bar{\gamma }}_{i1\cdot }+{\bar{r}}_{i1\cdot }\lambda \\ \mu_{2}+{\bar{\gamma }}_{i2\cdot }+{\bar{r}}_{i2\cdot }\lambda \\ \vdots \\ \mu_{t}+{\bar{\gamma }}_{it\cdot }+{\bar{r}}_{it\cdot }\lambda \\ \nu_{i1}^{\left(2\right)}\\ \vdots \\ \nu_{it}^{\left(2\right)} \end{array}\right]\text{or}=\left[\begin{array}{c} \mu_{1}+{\bar{\gamma }}_{i1\cdot }+\sum_{m=1}^{t}{\bar{r}}_{i1\cdot m}\lambda_{m}\\ \mu_{2}+{\bar{\gamma }}_{i2\cdot }+\sum_{m=1}^{t}{\bar{r}}_{i2\cdot m}\lambda_{m}\\ \vdots \\ \mu_{t}+{\bar{\gamma }}_{it\cdot }+\sum_{m=1}^{t}{\bar{r}}_{it\cdot m}\lambda_{m}\\ \nu_{i1}^{\left(3\right)}\\ \vdots \\ \nu_{it}^{\left(3\right)} \end{array}\right]\;\;\; \end{array}$$

for the models in Equations (1), (3), and (6), respectively. In Equation (17), the ${\bar{\gamma }}_{ik\cdot }$’s are averages of the $\gamma_{ikl}$’s, the ${\bar{r}}_{ik\cdot }$’s are averages of the $r_{ikl}$’s, and the $\nu_{ik}^{\left(1\right)}$’s, $\nu_{ik}^{\left(2\right)}$’s, and $\nu_{ik}^{\left(3\right)}$’s are nuisance parameters. The variance matrix of the vector of transformed variables in Equation (16) is

(18) $$\begin{array}{ccc} & & \text{var}\left[\begin{array}{c} {\bar{Y}}_{ij1\cdot }\\ {\bar{Y}}_{ij2\cdot }\\ \vdots \\ {\bar{Y}}_{ijt\cdot }\\ Y_{ij1}^{*}\\ \vdots \\ Y_{ijt}^{*} \end{array}\right]\\ & & =\left[\begin{array}{cccccccc} & \Omega_{11}+\frac{1}{p_{1}}\Sigma_{1} & \Omega_{12} & \cdots & \Omega_{1t} & 0 & \cdots & 0\\ & \Omega_{21} & \Omega_{22}+\frac{1}{p_{2}}\Sigma_{2} & \cdots & \Omega_{2t} & 0 & \cdots & 0\\ & \vdots & \vdots & \ddots & \vdots & \vdots & \ddots & \vdots \\ & \Omega_{t1} & \Omega_{t2} & \cdots & \Omega_{tt}+\frac{1}{p_{t}}\Sigma_{t} & 0 & \cdots & 0\\ & 0 & 0 & \cdots & 0 & I_{\left(p_{1}-1\right)}⊗\Sigma_{1} & \cdots & 0\\ & \vdots & \vdots & \ddots & \vdots & \vdots & \ddots & \vdots \\ & 0 & 0 & \cdots & 0 & 0 & \cdots & I_{\left(p_{t}-1\right)}⊗\Sigma_{t} \end{array}\right] \end{array}$$

From Equation (18), we observe that for each subject, the sample treatment means and the sample treatment orthogonal contrasts are independent. Let $\Omega^{*}$ denote the qt×qt submatrix within Equation (18).

(19) $$\begin{array}{ccc} \Omega^{*}=\text{var}\left[\begin{array}{c} {\bar{Y}}_{ij1\cdot }\\ {\bar{Y}}_{ij2\cdot }\\ \vdots \\ {\bar{Y}}_{ijt\cdot } \end{array}\right] & = & \left[\begin{array}{ccccc} & \Omega_{11}+\frac{1}{p_{1}}\Sigma_{1} & \Omega_{12} & \cdots & \Omega_{1t}\\ & \Omega_{21} & \Omega_{22}+\frac{1}{p_{2}}\Sigma_{2} & \cdots & \Omega_{2t}\\ & \vdots & \vdots & \ddots & \vdots \\ & \Omega_{t1} & \Omega_{t2} & \cdots & \Omega_{tt}+\frac{1}{p_{t}}\Sigma_{t} \end{array}\right]\\ & \overset{\Delta }{=} & \left[\begin{array}{ccccc} & \Omega_{11}^{*} & \Omega_{12}^{*} & \cdots & \Omega_{1t}^{*}\\ & \Omega_{21}^{*} & \Omega_{22}^{*} & \cdots & \Omega_{2t}^{*}\\ & \vdots & \vdots & \ddots & \vdots \\ & \Omega_{t1}^{*} & \Omega_{t2}^{*} & \cdots & \Omega_{tt}^{*} \end{array}\right] \end{array}$$

This represents a one‐to‐one reparameterization from Ω to $\Omega^{*}$. The likelihood‐based estimators of $\Omega^{*}$ and the set of intra‐subject variance matrices $\Sigma_{1},\text{…},\Sigma_{t}$ can be obtained separately. The corresponding likelihood‐based estimators of Ω are then derived by applying the inverse transformation to the estimators of $\Omega^{*}$.

The vectors $\text{vec}\left[{\bar{Y}}_{ij1\cdot }\cdots {\bar{Y}}_{ijt\cdot }\right]$, for i=1,…,s and $j=1,\text{…},n_{i}$, constitute a one‐way multivariate analysis of variance (MANOVA) model with homogeneous covariance matrix $\Omega^{*}$. Let ${\bar{Y}}_{ij}=\text{vec}\left[{\bar{Y}}_{ij1\cdot }\cdots {\bar{Y}}_{ijt\cdot }\right]$, so that $\text{var}\left({\bar{Y}}_{ij}\right)=\Omega^{*}$. In this MANOVA setup, the ith sample consists of ${\bar{Y}}_{i1},{\bar{Y}}_{i2},\text{…},{\bar{Y}}_{in_{i}}$, for i=1,2,…,s. For the ith sample, let $S_{i}$ denote the qt×qt sum of squares and cross‐products (SSCP) matrix.

(20) $$S_{i}=\sum_{j=1}^{n_{i}}\left({\bar{Y}}_{ij}-{\bar{Y}}_{i}\right){\left({\bar{Y}}_{ij}-{\bar{Y}}_{i}\right)}^{T}$$

in which

(21) $${\bar{Y}}_{i}=\frac{1}{n_{i}}\sum_{j=1}^{n_{i}}{\bar{Y}}_{ij}=\frac{1}{n_{i}}\left[\begin{array}{c} \sum_{j=1}^{n_{i}}{\bar{Y}}_{ij1\cdot }\\ \vdots \\ \sum_{j=1}^{n_{i}}{\bar{Y}}_{ijt\cdot } \end{array}\right]\overset{\Delta }{=}\left[\begin{array}{c} {\bar{Y}}_{i\cdot 1\cdot }\\ \vdots \\ {\bar{Y}}_{i\cdot t\cdot } \end{array}\right]$$

Then the SSCP matrix $S_{i}$ becomes

(22) $$S_{i}={\left(\begin{array}{c} \left(\sum_{j=1}^{n_{i}}\left({\bar{Y}}_{ijk\cdot }-{\bar{Y}}_{i\cdot k\cdot }\right){\left({\bar{Y}}_{ijk^{\prime }\cdot }-{\bar{Y}}_{i\cdot k^{\prime }\cdot }\right)}^{T}\right) \end{array}\right)}_{k,k^{\prime }=1,\text{…},t}$$

The likelihood‐based estimators of $\Omega^{*}$ are

(23) $${\hat{\Omega }}_{\mathrm{ML}}^{*}=\frac{1}{n}\sum_{i=1}^{s}S_{i}\;\text{and}\;{\hat{\Omega }}_{\mathrm{REML}}^{*}=\frac{1}{n-s}\sum_{i=1}^{s}S_{i}$$

The derivations of the likelihood‐based estimators of the intra‐subject variance‐covariance matrices can be found in Appendix A.2. The maximum likelihood estimators of $\Sigma_{k}$, k=1,…,t are

(24) $${\hat{\Sigma }}_{k,\mathrm{ML}}=\frac{1}{n(p_{k}-1)}\sum_{i=1}^{s}\sum_{j=1}^{n_{i}}\sum_{l=1}^{p_{k}-1}\left(Y_{ijk,l}^{*}-{\bar{Y}}_{i\cdot k,l}^{*}\right){\left(Y_{ijk,l}^{*}-{\bar{Y}}_{i\cdot k,l}^{*}\right)}^{T}$$

The REML estimators of $\Sigma_{k}$, k=1,…,t are

(25) $$\begin{array}{c} {\hat{\Sigma }}_{k,\mathrm{REML}}\;=\;\frac{1}{\left(n-s\right)\left(p_{k}-1\right)}\sum_{i=1}^{s}\sum_{j=1}^{n_{i}}\sum_{l=1}^{p_{k}-1}\left(Y_{ijk,l}^{*}-{\bar{Y}}_{i\cdot k,l}^{*}\right){\left(Y_{ijk,l}^{*}-{\bar{Y}}_{i\cdot k,l}^{*}\right)}^{T}\;\;\;\; \end{array}$$

where ${\bar{Y}}_{i\cdot k,l}^{*}=\frac{1}{n_{i}}\sum_{j=1}^{n_{i}}Y_{ijk,l}^{*}$ in Equations (24) and (25). Finally, we apply the inverse transformation from $\Omega^{*}$ to Ω, which yields the likelihood‐based estimators of Ω:

(26) $$\begin{array}{cc} {\hat{\Omega }}_{\mathrm{ML}} & ={\hat{\Omega }}_{\mathrm{ML}}^{*}-\text{diag}\left(\frac{1}{p_{1}}{\hat{\Sigma }}_{1,\mathrm{ML}},\frac{1}{p_{2}}{\hat{\Sigma }}_{2,\mathrm{ML}},\text{…},\frac{1}{p_{t}}{\hat{\Sigma }}_{t,\mathrm{ML}}\right)\\ {\hat{\Omega }}_{\mathrm{REML}} & ={\hat{\Omega }}_{\mathrm{REML}}^{*}-\text{diag}\left(\frac{1}{p_{1}}{\hat{\Sigma }}_{1,\mathrm{REML}},\frac{1}{p_{2}}{\hat{\Sigma }}_{2,\mathrm{REML}},\text{…},\frac{1}{p_{t}}{\hat{\Sigma }}_{t,\mathrm{REML}}\right) \end{array}$$

### 2.4 Hypothesis Testing

#### 2.4.1 Hypothesis Tests for Comparing Treatment Effects and Covariate Effects

Typically, we are interested in testing the null hypothesis that two treatment effects are equal, that is, $H_{0}:\mu_{k}=\mu_{k^{\prime }}$ for $k\neq k^{\prime }$, where $k,k^{\prime }=1,\text{…},t$. In general settings, by fitting nested models and substituting the maximum likelihood estimators of $\beta^{*}$ and the $\Lambda_{i}$’s into the log‐likelihood function defined in Equation (13), we can construct likelihood ratio statistics to test such null hypotheses. Likelihood ratio statistics can also be used to test whether any covariate effects are null.

There exists a special case in which an exact test for comparing treatment effects can be constructed. Suppose there are no baseline covariates in the model and the crossover design is uniform within sequences; that is, $p_{1k}=\cdots =p_{sk}\left(=p_{k}\right)$ for each treatment k=1,2,…,t. We collect the population mean vectors into a t×q matrix μ:

(27) $$\mu =\left[\begin{array}{c} \mu_{1}^{T}\\ \vdots \\ \mu_{t}^{T} \end{array}\right]$$

The general linear hypothesis for the multivariate linear model in this special case is

(28) $$H_{0}:C\mu U=0$$

where C is a known, full‐rank c×t matrix with c≤t, and U is a known, full‐rank q×u matrix with u≤q. In crossover trials, we are typically interested in comparing treatment effect vectors with respect to all q outcomes; that is, $U=I_{q\times q}$. Under this setting, the null hypothesis of interest reduces to

(29) $$H_{0}:C\mu =0$$

where C is a c×t contrast matrix of full rank (c≤t) and each row of C sums of zero. This null hypothesis is equivalent to

(30) $$H_{0}:\text{vec}\left[{\left(C\mu \right)}^{T}\right]=0$$

From Equations (17) and (18), it follows that the sample means and the orthogonal contrasts are independent. Therefore, statistical inference concerning μ can focus solely on the t×q observation matrices $\left[\begin{array}{c} {\bar{Y}}_{ij1\cdot }^{T}\\ \vdots \\ {\bar{Y}}_{ijt\cdot }^{T} \end{array}\right]$, i=1,2,…,s,j=1,2, $\text{…},n_{i}$. We let

(31) $$\hat{\mu }=\left[\begin{array}{c} \frac{1}{s}\sum_{i=1}^{s}{\bar{Y}}_{i\cdot 1\cdot }^{T}\\ \vdots \\ \frac{1}{s}\sum_{i=1}^{s}{\bar{Y}}_{i\cdot t\cdot }^{T} \end{array}\right]$$

We can then show that the ML and REML estimators of $\text{vec}(\mu^{T})$ under the model specified in Equation (1) are given by $\text{vec}({\hat{\mu }}^{T})$. This implies that $\hat{\mu }$ is the maximum likelihood estimator of μ for the model in Equation (1). The corresponding variance matrix is

(32) $$\text{var}\left[\text{vec}\left({\hat{\mu }}^{T}\right)\right]=\frac{1}{s^{2}}\left(\sum_{i=1}^{s}\frac{1}{n_{i}}\right)\Omega^{*}$$

where $\Omega^{*}$ is defined in Equation (19). Although $\hat{\mu }$ is not the likelihood‐based estimator of μ under the models in Equations (3) and (6), if C is a c×t contrast matrix with c≤t (i.e., each row of C sums to zero), then $C\hat{\mu }$ serves as both the ML and REML estimator of Cμ, regardless of the model used.

An exact test of the null hypothesis $H_{0}:\text{vec}\left[{\left(C\mu \right)}^{T}\right]=0$ versus the alternative $H_{1}:\text{vec}\left[{\left(C\mu \right)}^{T}\right]\neq 0$ is available. Similar to the estimation procedure, we construct a one‐way MANOVA model with homogeneous variance $\Omega^{*}$ using the vectors $\text{vec}\left[{\bar{Y}}_{ij1\cdot }\cdots {\bar{Y}}_{ijt\cdot }\right]$, for i=1,…,s and $j=1,\text{…},n_{i}$. Under this framework, it can be shown that $\text{vec}[{\left(C\hat{\mu }\right)}^{T}]$ and $\left(C⊗I_{q\times q}\right)\;{\hat{\Omega }}_{\mathrm{REML}}^{*}\;{\left(C⊗I_{q\times q}\right)}^{T}$ are independently distributed as a multivariate normal and a Wishart variate, respectively, where ${\hat{\Omega }}_{\mathrm{REML}}^{*}$ is defined in Equation 23):

(33) $$\begin{array}{cc} & \text{vec}[{\left(C\hat{\mu }\right)}^{T}]\\ & \;=\left(C⊗I_{q\times q}\right)\text{vec}\left({\hat{\mu }}^{T}\right)\\ & \;∼N_{qc}\left(\text{vec}\left[{\left(C\mu \right)}^{T}\right],\frac{1}{s^{2}}\left(\sum_{i=1}^{s}\frac{1}{n_{i}}\right)\left(C⊗I_{q\times q}\right)\Omega^{*}{\left(C⊗I_{q\times q}\right)}^{T}\right) \end{array}$$

(34) $$\begin{array}{ccc} & & \left(n-s\right)\left(C⊗I_{q\times q}\right){\hat{\Omega }}_{\mathrm{REML}}^{*}{\left(C⊗I_{q\times q}\right)}^{T}\\ & & \;∼W_{qc}\left(n-s,\left(C⊗I_{q\times q}\right)\Omega^{*}{\left(C⊗I_{q\times q}\right)}^{T}\right) \end{array}$$

The above two independent distributions render a Hotelling's $T^{2}$ statistic of the form

(35) $$\begin{array}{ccc} & & \frac{n-s-qc+1}{qc(n-s)}\text{vec}{\left[{\left(C\hat{\mu }\right)}^{T}\right]}^{T}\\ & & \;\times {\left[\frac{1}{s^{2}}\left(\sum_{i=1}^{s}\frac{1}{n_{i}}\right)\left(C⊗I_{q\times q}\right){\hat{\Omega }}_{\mathrm{REML}}^{*}{\left(C⊗I_{q\times q}\right)}^{T}\right]}^{-1}\text{vec}\left[{\left(C\hat{\mu }\right)}^{T}\right]\\ & & \;∼F_{qc,n-s-qc+1} \end{array}$$

#### 2.4.2 Hypothesis Tests for Comparing Intra‐Subject Variance Matrices

In general settings, by fitting nested models and substituting the maximum likelihood estimators of $\beta^{*}$ and the $\Lambda_{i}$’s into the log‐likelihood function defined in Equation (13), we can construct a likelihood ratio test for the null hypothesis that two intra‐subject variance matrices are equal; that is, $H_{0}:\Sigma_{k}=\Sigma_{k^{\prime }}$, for $k\neq k^{\prime }$, where $k,k^{\prime }=1,\text{…},t$.

Similarly, there exists a special case where an exact test is available for the null hypothesis

(36) $$H_{0}:\Sigma_{1}=\Sigma_{2}=\cdots =\Sigma_{t}$$

versus

(37) $$H_{1}:\text{not all}\;\Sigma_{1},\Sigma_{2},\text{…},\Sigma_{t}\;\text{are equal}$$

Suppose there are no baseline covariates in the model, the crossover design is uniform within sequences, and each $p_{k}\geq 2$ for k=1,2,…,t. Under this special case, the REML estimators of the t intra‐subject variance matrices $\Sigma_{k}$ (k=1,…,t) are given in Equation (25). Owing to the independence of subjects and the independence of the $Y_{ijk,l}^{*}$’s ($k=1,\text{…},t;\;l=1,\text{…},p_{k}-1$) within each subject, the REML estimators of the $\Sigma_{k}$’s, after removing the denominators, are mutually independent, and each follows a central Wishart distribution:

(38) $$\begin{array}{c} \left(n-s\right)\left(p_{k}-1\right){\hat{\Sigma }}_{k,\mathrm{REML}}∼W_{q}\left(\left(n-s\right)\left(p_{k}-1\right),\Sigma_{k}\right),\;k=1,\text{…},t \end{array}$$

We can derive a likelihood ratio test based on the central Wishart distribution for the REML estimates. Define

(39) $$A_{k}=\left(n-s\right)\left(p_{k}-1\right){\hat{\Sigma }}_{k,\mathrm{REML}}∼W_{q}\left(\left(n-s\right)\left(p_{k}-1\right),\Sigma_{k}\right)$$

 $A_{k}$’s (k=1,…,t) are independent. Then, based on the probability density function of a central Wishart distribution, the log‐likelihood function for $A_{k}$’s (k=1,…,t) is

(40) $$\begin{array}{ccc} l & = & \sum_{k=1}^{t}\left[\log\left(\frac{1}{2^{qc_{k}}\Gamma_{q}\left(c_{k}\right)}\right)+c_{k}\log\left[\det\left(\Sigma_{k}^{-1}\right)\right]\right.\\ & & \left.+\mathrm{trace}\left(-\frac{1}{2}\Sigma_{k}^{-1}A_{k}\right)+\log\left({\left[\det\left(A_{k}\right)\right]}^{c_{k}-\frac{q+1}{2}}\right)\right] \end{array}$$

where $c_{k}$ is a constant defined as $c_{k}=\frac{\left(p_{k}-1\right)\left(n-s\right)}{2}$. The MLE based on the log‐likelihood is

(41) $$\begin{array}{ccc} {\hat{\Sigma }}_{k} & = & \frac{1}{\left(p_{k}-1\right)\left(n-s\right)}A_{k}\\ & = & \frac{1}{\left(p_{k}-1\right)\left(n-s\right)}\sum_{i=1}^{s}\sum_{j=1}^{n_{i}}\sum_{l=1}^{p_{k}-1}\left(Y_{ijk,l}^{*}-{\bar{Y}}_{i\cdot k,l}^{*}\right){\left(Y_{ijk,l}^{*}-{\bar{Y}}_{i\cdot k,l}^{*}\right)}^{T} \end{array}$$

Then investigate the log‐likelihood and the corresponding MLE under $H_{0}:\Sigma_{1}=\Sigma_{2}=\cdots =\Sigma_{t}=\Sigma$. Under $H_{0}$, $A_{k}$’s (k=1,…,t) independently follow central Wishart distribution, $A_{k}∼W_{q}\left(\left(n-s\right)\left(p_{k}-1\right),\Sigma \right)$. The log‐likelihood is

(42) $$\begin{array}{ccc} l_{0} & = & \sum_{k=1}^{t}\left[\log\left(\frac{1}{2^{qc_{k}}\Gamma_{q}\left(c_{k}\right)}\right)+c_{k}\log\left(\det\left(\Sigma^{-1}\right)\right)\right.\\ & & \left.+\mathrm{trace}\left(-\frac{1}{2}\Sigma^{-1}A_{k}\right)+\log\left({\left[\det\left(A_{k}\right)\right]}^{c_{k}-\frac{q+1}{2}}\right)\right] \end{array}$$

where $c_{k}=\frac{\left(p_{k}-1\right)\left(n-s\right)}{2}$. The MLE of Σ based on the log‐likelihood is

(43) $$\hat{\Sigma }=\frac{1}{\left(n-s)(p-t\right)}\sum_{i=1}^{s}\sum_{j=1}^{n_{i}}\sum_{k=1}^{t}\sum_{l=1}^{p_{k}-1}\left(Y_{ijk,l}^{*}-{\bar{Y}}_{i\cdot k,l}^{*}\right){\left(Y_{ijk,l}^{*}-{\bar{Y}}_{i\cdot k,l}^{*}\right)}^{T}$$

We can contruct the likelihood ratio statistic as

(44) $$\begin{array}{cc} T_{LR} & =-2[l_{0}\left(\hat{\Sigma }\right)-l\left({\hat{\Sigma }}_{1},\text{…},{\hat{\Sigma }}_{t}\right)]\\ & =\left(n-s\right)\left(p-t\right)\log\left(\det\left(\hat{\Sigma }\right)\right)-\sum_{k=1}^{t}\left(p_{k}-1\right)\left(n-s\right)\log\left(\det\left({\hat{\Sigma }}_{k}\right)\right) \end{array}$$

This likelihood ratio statistic asymptotically follows a chi‐square distribution with degrees of freedom $df=\left(t-1\right)\frac{q(q+1)}{2}$.

## 3 Simulation Study

In this section, we conduct a simulation study to evaluate whether the proposed multivariate approach outperforms the conventional univariate approach in hypothesis testing for treatment effect comparisons. The simulation datasets are generated based on the TALC study, a three‐treatment, three‐period, six‐sequence crossover trial described in Section 4. The three treatments are denoted as treatment A, treatment B, and treatment C, and the six sequences are ABC, ACB, BAC, BCA, CAB, and CBA. There are four continuous outcomes of interest. In this study, we focus on comparing the mean responses between treatment A and treatment B. The performance of the multivariate hypothesis testing procedure is assessed by comparing its statistical power to that of the univariate approach under varying levels of treatment mean differences.

### 3.1 Simulation Set‐Up

In the simulation study, we generate crossover trial datasets that closely resemble the data from the TALC study described in Section 4. Throughout the simulation, we fix the sample size of the generated datasets to 187, consistent with the TALC study original dataset. For each subject, we retain the same covariate values (such as sequence and study center) as observed in the original dataset. The simulated data are generated using the model

(45) $$Y_{ijkl}=\mu_{k}+\left(I_{q\times q}⊗X_{ijkl}^{T}\right)\beta +r_{ikl}\lambda +\gamma_{ikl}+u_{ijk}$$

This is the same model as that specified in Equation (3), except that the intra‐subject variability term is omitted, as it is inestimable when there are no replications of treatments within sequences. With the exception of the mean effect vector for treatment A ($\mu_{A}$), the REML estimates obtained from the TALC dataset (as reported in Section 4) for the remaining fixed effects—including the treatment B and C effects, study center (stratifying variable) coefficients, and nuisance parameters as well as the variance components, are used as parameters for data generation.

To compare the statistical power of the multivariate and univariate approaches under varying levels of treatment mean difference, the mean vector for treatment A, $\mu_{A}$, is set based on the REML estimate of the treatment B effect, ${\hat{\mu }}_{B,\mathrm{REML}}$. To evaluate type I error, we first set $\mu_{A}={\hat{\mu }}_{B,\mathrm{REML}}=\mu_{B}$. Next, to investigate statistical power for testing $H_{0}:\mu_{A}=\mu_{B}$, we consider scenarios in which 10% mean differences are introduced between some (or all) components of $\mu_{A}$ and $\mu_{B}$. In these cases, while maintaining ${\hat{\mu }}_{B,\mathrm{REML}}$ as the baseline for $\mu_{A}$, we multiply selected components of $\mu_{A}$ by a factor of 1.1.

Since $\mu_{A}$ is a four‐dimensional vector, we consider all nonempty subsets of its components for modification, yielding a total of 15 distinct scenarios. For each scenario, 1000 datasets are generated. Similarly, we assess statistical power under 20% mean differences and in an additional scenario where the components of $\mu_{A}$ are scaled by the vector (1.15,1.1,1.15,1.2).

For each simulated dataset, we fit the multivariate linear model specified in Equation (45) using maximum likelihood (ML) estimation. We also fit a reduced multivariate model under the null hypothesis $H_{0}:\mu_{A}=\mu_{B}$, also using ML estimation. A likelihood ratio test is then conducted based on the log‐likelihood values of the two models. The resulting test statistic, $T_{\text{LR}}$, asymptotically follows a $\chi_{4}^{2}$ distribution. The empirical type I error rate and statistical power of the multivariate approach are calculated as the proportion of the 1000 simulated datasets in which $T_{\text{LR}}$ exceeds the critical value $\chi_{4,0.95}^{2}$.

For the univariate approach, we conduct a separate Student's t‐test for each outcome component, based on the ML estimates of the treatment effects and their standard errors. To account for multiple comparisons, a Bonferroni correction is applied. The overall multivariate null hypothesis is considered rejected if at least one of the four individual univariate null hypotheses is rejected. The empirical type I error rate and power of the univariate approach are defined as the proportion of simulations in which the multivariate null hypothesis is rejected across the 1000 simulated datasets.

To provide context for simulation variability, note that with 1000 simulation runs, the standard error of an estimated probability is $\sqrt{p(1-p)/1000}$. In particular, this is approximately 0.0069 when p=0.05, and at most approximately 0.016 (attained at p=0.5).

### 3.2 Simulation Results

The empirical type I error rate of the multivariate approach is 0.053, which is close to the nominal rate of 0.05 and within Monte Carlo variability. In contrast, the univariate approach yields an empirical type I error rate of 0.038, which is slightly below the nominal level of 0.05; however, the exact binomial 95% confidence interval is (0.027, 0.052), indicating that this estimate is within the expected Monte Carlo variability and is not statistically distinguishable from the nominal level.

Let ⊙ denote the element‐wise (Hadamard) product for vectors. Table 1 presents the statistical power of the multivariate and univariate approaches under various configurations of $\mu_{A}$, where one or more components of the treatment A mean vector are increased by 10% relative to $\mu_{B}$. An overall increasing pattern in the power of the univariate approach is observed as more components of $\mu_{A}$ deviate from $\mu_{B}$. This may be attributed to the testing strategy, which rejects the multivariate null hypothesis if any of the univariate null hypotheses are rejected, which may increase the chance of rejection when multiple components differ.

**TABLE 1:** Statistical power for testing $H_{0}:\mu_{A}=\mu_{B}$, under different $\mu_{A}$ configurations (10% mean increase).

Case No.	Modified variables	$\mu_{A}$ configuration	Seed	Multivariate power	Univariate power
1	(1)	$\left(1.1,1,1,1\right)⊙\mu_{B}$	952163715	0.2640	0.0910
2	(2)	$\left(1,1.1,1,1\right)⊙\mu_{B}$	579938472	0.8830	0.1460
3	(3)	$\left(1,1,1.1,1\right)⊙\mu_{B}$	464992795	0.1730	0.1020
4	(4)	$\left(1,1,1,1.1\right)⊙\mu_{B}$	318761843	0.3890	0.1240
5	(1,2)	$\left(1.1,1.1,1,1\right)⊙\mu_{B}$	150064306	0.5440	0.1410
6	(1,3)	$\left(1.1,1,1.1,1\right)⊙\mu_{B}$	578964932	0.3890	0.1230
7	(1,4)	$\left(1.1,1,1,1.1\right)⊙\mu_{B}$	774756114	0.8650	0.1540
8	(2,3)	$\left(1,1.1,1.1,1\right)⊙\mu_{B}$	724722169	0.8720	0.1740
9	(2,4)	$\left(1,1.1,1,1.1\right)⊙\mu_{B}$	308362909	0.4660	0.1890
10	(3,4)	$\left(1,1,1.1,1.1\right)⊙\mu_{B}$	665810388	0.5440	0.1450
11	(1,2,3)	$\left(1.1,1.1,1.1,1\right)⊙\mu_{B}$	737769486	0.4340	0.1860
12	(1,2,4)	$\left(1.1,1.1,1,1.1\right)⊙\mu_{B}$	247016352	0.2830	0.1970
13	(1,3,4)	$\left(1.1,1,1.1,1.1\right)⊙\mu_{B}$	304984445	0.8950	0.1650
14	(2,3,4)	$\left(1,1.1,1.1,1.1\right)⊙\mu_{B}$	659557202	0.3640	0.1970
15	(1,2,3,4)	$1.1\times \mu_{B}$	422645571	0.1830	0.2070

For the multivariate approach, the statistical power tends to increase initially as more components differ from $\mu_{B}$, but may decrease when all components are modified. Despite this non‐monotonic pattern, the multivariate approach generally achieves higher power than the univariate approach in most scenarios. When all components of the outcome vector are increased by a factor of 1.1, the statistical power of the two approaches becomes comparable.

Similar patterns are observed in Table 2, which reports results for 20% increases. Table 3 shows the power when the mean vector of treatment A is increased from that of treatment B by an arbitrary vector of factors (15%,10%,15%,20%). In this setting, the univariate approach still exhibits increasing power as more components are modified. However, in contrast to earlier observations, the multivariate approach does not show a drop in power when all components are increased. Across all configurations in this scenario, the multivariate approach demonstrates consistently higher statistical power than the univariate approach.

**TABLE 2:** Statistical power for testing $H_{0}:\mu_{A}=\mu_{B}$, under different $\mu_{A}$ configurations (20% mean increase).

Case No.	Modified variables	$\mu_{A}$ configuration	Seed	Multivariate power	Univariate power
1	(1)	$\left(1.2,1,1,1\right)⊙\mu_{B}$	708465414	0.8260	0.2720
2	(2)	$\left(1,1.2,1,1\right)⊙\mu_{B}$	678619043	1.0000	0.5560
3	(3)	$\left(1,1,1.2,1\right)⊙\mu_{B}$	141597709	0.5520	0.2820
4	(4)	$\left(1,1,1,1.2\right)⊙\mu_{B}$	590627186	0.9600	0.4210
5	(1,2)	$\left(1.2,1.2,1,1\right)⊙\mu_{B}$	797610800	0.9920	0.6070
6	(1,3)	$\left(1.2,1,1.2,1\right)⊙\mu_{B}$	984071851	0.9390	0.3780
7	(1,4)	$\left(1.2,1,1,1.2\right)⊙\mu_{B}$	103702586	1.0000	0.5460
8	(2,3)	$\left(1,1.2,1.2,1\right)⊙\mu_{B}$	918551906	1.0000	0.6060
9	(2,4)	$\left(1,1.2,1,1.2\right)⊙\mu_{B}$	336753264	0.9710	0.6060
10	(3,4)	$\left(1,1,1.2,1.2\right)⊙\mu_{B}$	437852689	0.9950	0.5440
11	(1,2,3)	$\left(1.2,1.2,1.2,1\right)⊙\mu_{B}$	687234581	0.9640	0.5920
12	(1,2,4)	$\left(1.2,1.2,1,1.2\right)⊙\mu_{B}$	478873831	0.8200	0.6330
13	(1,3,4)	$\left(1.2,1,1.2,1.2\right)⊙\mu_{B}$	679928235	1.0000	0.6290
14	(2,3,4)	$\left(1,1.2,1.2,1.2\right)⊙\mu_{B}$	132644359	0.9300	0.6500
15	(1,2,3,4)	$1.2\times \mu_{B}$	575129698	0.5570	0.6570

**TABLE 3:** Statistical power for testing $H_{0}:\mu_{A}=\mu_{B}$, under different $\mu_{A}$ configurations (mean increase by an arbitrary vector of factors, (15%, 10%, 15%, 20%)).

Case No.	Modified variables	$\mu_{A}$ configuration	Seed	Multivariate power	Univariate power
1	(1)	$\left(1.15,1,1,1\right)⊙\mu_{B}$	456870290	0.5760	0.1370
2	(2)	$\left(1,1.1,1,1\right)⊙\mu_{B}$	898483049	0.8870	0.1540
3	(3)	$\left(1,1,1.15,1\right)⊙\mu_{B}$	652966902	0.3380	0.1870
4	(4)	$\left(1,1,1,1.2\right)⊙\mu_{B}$	497067242	0.9470	0.4220
5	(1,2)	$\left(1.15,1.1,1,1\right)⊙\mu_{B}$	234816698	0.4280	0.1830
6	(1,3)	$\left(1.15,1,1.15,1\right)⊙\mu_{B}$	531015519	0.7260	0.2330
7	(1,4)	$\left(1.15,1,1,1.2\right)⊙\mu_{B}$	170237910	1.0000	0.4800
8	(2,3)	$\left(1,1.1,1.15,1\right)⊙\mu_{B}$	341052939	0.8700	0.2310
9	(2,4)	$\left(1,1.1,1,1.2\right)⊙\mu_{B}$	329350957	0.4890	0.4420
10	(3,4)	$\left(1,1,1.15,1.2\right)⊙\mu_{B}$	311292379	0.9780	0.4900
11	(1,2,3)	$\left(1.15,1.1,1.15,1\right)⊙\mu_{B}$	119268538	0.3150	0.2560
12	(1,2,4)	$\left(1.15,1.1,1,1.2\right)⊙\mu_{B}$	831915208	0.8390	0.4670
13	(1,3,4)	$\left(1.15,1,1.15,1.2\right)⊙\mu_{B}$	298812116	1.0000	0.4990
14	(2,3,4)	$\left(1,1.1,1.15,1.2\right)⊙\mu_{B}$	659485510	0.5150	0.4720
15	(1,2,3,4)	$\left(1.15,1.1,1.15,1.2\right)⊙\mu_{B}$	391133509	0.8310	0.5000

These results suggest that the proposed multivariate approach is generally preferable for testing treatment effect differences in the presence of multiple correlated outcomes, as it tends to achieve higher statistical power across a range of scenarios while maintaining type I error rates comparable to the nominal level.

## 4 Data Application

### 4.1 The TALC Dataset

Peters et al. (2010) reported a crossover trial titled *Tiotropium Bromide as an Alternative to Increased Inhaled Glucocorticoid in Patients Inadequately Controlled on a Lower Dose of Inhaled Corticosteroid* (TALC study). This crossover clinical trial randomized 210 adult patients into six treatment sequences. All participants received a background therapy of beclomethasone 80 μg twice daily throughout the study, including washout periods. The three treatments being compared in addition to the background therapy are: (A) an additional dose of tiotropium bromide 18 μg every morning; (B) an additional dose of beclomethasone 80 μg twice daily (i.e., doubling the beclomethasone dose); and (C) an additional dose of salmeterol xinafoate 50 μg twice daily. The six treatment sequences were ABC, ACB, BAC, BCA, CAB, and CBA. The primary objectives of the study were to determine whether treatment A was superior to treatment B and whether treatment A was non‐inferior to treatment C.

In this data analysis example, we consider the changes from baseline in four lung function measurements as the multivariate response variable: forced vital capacity (FVC), forced expiratory volume in 1 s (FEV1), peak expiratory flow rate (PEFR), and forced mid‐expiratory flow (${\mathrm{FEF}}_{25-75}$). These outcomes are all routinely reported in asthma clinical trials and capture complementary aspects of pulmonary function, including lung volume, airflow limitation, peak expiratory flow, and mid‐expiratory flow. These measures are known to be correlated due to shared physiological mechanisms. The purpose of their joint inclusion here is to provide a realistic example involving multiple correlated outcomes in order to illustrate the practical application of the proposed multivariate modeling framework to real‐world clinical trial data. The crossover design is uniform and balanced with respect to first‐order carryover effects, meaning that treatment effects are not aliased with a common carryover effect. Therefore, we adopt the model specified in Equation (3).

Since randomization was stratified by study center, we include study center in the model as a blocking factor to account for the stratified randomization design. Intra‐subject variances cannot be estimated due to the absence of treatment replication within sequences. Consequently, we estimate only $\Omega^{*}$ in Equation (19), which reflects inseparable inter‐ and intra‐subject variation (note that $p_{A}=p_{B}=p_{C}=1$ in this setting). A restricted maximum likelihood (REML) estimation procedure is implemented using the PROC MIXED procedure in SAS. The REML estimates of the treatment means and the common carryover effect are presented in Table 4. Each fixed effect has four associated estimates, corresponding to the four outcome variables in the multivariate response.

**TABLE 4:** Estimate (standard error) of treatment means and carryover effects.

	FVC	FEV1	PEFR	FEF2575
$\mu_{A}$	0.22 (0.11)	0.31 (0.10)	0.88 (0.34)	0.45 (0.14)
$\mu_{B}$	0.17 (0.11)	0.21 (0.10)	0.55 (0.34)	0.29 (0.14)
$\mu_{C}$	0.18 (0.12)	0.25 (0.10)	0.73 (0.34)	0.36 (0.15)
λ	−0.03 (0.02)	−0.02 (0.01)	0.01 (0.05)	−0.03 (0.02)

For ease of presentation, we do not report the REML estimates of the remaining 14 nuisance parameter vectors or the 9 coefficient vectors associated with the center covariate. The REML estimate of the variance matrix $\Omega^{*}$ in Equation (19) is

(46) $$\begin{array}{ccc} & & {\hat{\Omega }}_{\mathrm{REML}}^{*}\\ & & =\left[\begin{array}{cccccccccccc} 0.07 & 0.05 & 0.13 & 0.02 & 0.03 & 0.02 & 0.04 & 0.01 & 0.04 & 0.03 & 0.06 & 0.02\\ 0.05 & 0.05 & 0.12 & 0.05 & 0.02 & 0.02 & 0.03 & 0.02 & 0.03 & 0.03 & 0.05 & 0.03\\ 0.13 & 0.12 & 0.70 & 0.11 & 0.04 & 0.05 & 0.26 & 0.03 & 0.06 & 0.07 & 0.31 & 0.08\\ 0.02 & 0.05 & 0.11 & 0.12 & 0.01 & 0.02 & 0.01 & 0.04 & 0.02 & 0.03 & 0.03 & 0.06\\ 0.03 & 0.02 & 0.04 & 0.01 & 0.06 & 0.03 & 0.07 & 0.01 & 0.02 & 0.02 & 0.03 & 0.01\\ 0.02 & 0.02 & 0.05 & 0.02 & 0.03 & 0.04 & 0.07 & 0.04 & 0.02 & 0.02 & 0.05 & 0.02\\ 0.04 & 0.03 & 0.26 & 0.01 & 0.07 & 0.07 & 0.44 & 0.04 & 0.02 & 0.03 & 0.24 & 0.03\\ 0.01 & 0.02 & 0.03 & 0.04 & 0.01 & 0.04 & 0.04 & 0.09 & 0.02 & 0.03 & 0.06 & 0.04\\ 0.04 & 0.03 & 0.06 & 0.02 & 0.02 & 0.02 & 0.02 & 0.02 & 0.06 & 0.04 & 0.09 & 0.03\\ 0.03 & 0.03 & 0.07 & 0.03 & 0.02 & 0.02 & 0.03 & 0.03 & 0.04 & 0.05 & 0.10 & 0.06\\ 0.06 & 0.05 & 0.31 & 0.03 & 0.03 & 0.05 & 0.24 & 0.06 & 0.09 & 0.10 & 0.57 & 0.12\\ 0.02 & 0.03 & 0.08 & 0.06 & 0.01 & 0.02 & 0.03 & 0.04 & 0.03 & 0.06 & 0.12 & 0.11 \end{array}\right]\;\;\; \end{array}$$

In expression (46), the 12 entries correspond to the four outcomes measured under each of the three treatments (A, B, and C). Specifically, the ordering of the entries is treatment A (FVC), treatment A (FEV1), treatment A (PEFR), treatment A (${\mathrm{FEF}}_{25-75}$), …, treatment C (${\mathrm{FEF}}_{25-75}$). Accordingly, the estimated 12×12 variance‐covariance matrix can be partitioned to nine 4×4 matrices, as shown in Figure B1. A detailed illustration of the block structure and an explicit interpretation of individual entries are provided in the Appendix B.

Using the ESTIMATE statements in PROC MIXED, we obtain the estimates, standard errors, and t‐test results for the pairwise treatment differences for each univariate outcome. These results are summarized in Table 5. For each of the four outcome variables, the point estimate of the mean difference between treatment A and treatment B is positive and statistically significant. Similarly, the point estimates of the mean differences between treatment A and treatment C are also positive and statistically significant across all outcomes. Since larger values are preferable for all four outcomes, these findings suggest that treatment A outperforms both treatments B and C.

**TABLE 5:** Estimates of pairwise treatment differences for each univariate outcome.

Label	Estimate	SE	DF	t value	Pr >|t|	Lower	Upper
FVC: Treatment A versus B	0.05	0.02	178	2.52	0.0125	0.01	0.09
FVC: Treatment A versus C	0.04	0.02	220	2.01	0.0461	0.00	0.08
FVC: Treatment B versus C	−0.01	0.02	202	−0.54	0.5929	−0.06	0.03
FEV1: Treatment A versus B	0.10	0.02	178	5.93	<0.0001	0.07	0.13
FEV1: Treatment A versus C	0.06	0.02	215	3.50	0.0006	0.03	0.09
FEV1: Treatment B versus C	−0.04	0.02	206	−2.21	0.0281	−0.07	−0.00
PEFR: Treatment A versus B	0.33	0.06	175	5.54	<0.0001	0.21	0.44
PEFR: Treatment A versus C	0.15	0.07	209	2.31	0.0221	0.02	0.28
PEFR: Treatment B versus C	−0.18	0.06	206	−2.92	0.0039	−0.30	−0.06
FEF2575: Treatment A versus B	0.16	0.03	177	5.77	<0.0001	0.10	0.21
FEF2575: Treatment A versus C	0.09	0.03	212	3.38	0.0009	0.04	0.14
FEF2575: Treatment B versus C	−0.07	0.03	205	−2.44	0.0156	−0.12	−0.01

We assess covariate effects and compare treatment effects by fitting both full and nested models using the ML estimation method, and constructing likelihood ratio tests. The results are presented in Table 6. The effect of study center is not statistically significant in the model (p=0.26). The effect of treatment A is significantly different from that of treatment B (p<0.0001). As shown in Table 4, for each outcome variable, the estimated effect of treatment A exceeds that of treatment B, indicating that treatment A is superior to treatment B.

**TABLE 6:** Likelihood ratio tests of covariate effects and treatment effects.

$H_{0}$	−2 log‐likeli (reduced)	−2 log‐likeli (full)	LR statistic	df	p‐value
$\beta_{\text{center}}=0$	−701.2	−742.1	40.90	36	0.2641
$\mu_{A}=\mu_{B}$	−697.9	−742.1	44.22	4	<0.0001
$\mu_{A}=\mu_{C}$	−728.5	−742.1	13.67	4	0.0084
$\mu_{B}=\mu_{C}$	−729.9	−742.1	12.22	4	0.0158
$\mu_{A}=\mu_{B}=\mu_{C}$	−692.1	−742.1	50.00	8	<0.0001

Similarly, the effect of treatment A is significantly different from that of treatment C (p=0.0084). Table 4 shows that, for each outcome variable, the estimated effect of treatment A is greater than that of treatment C. These results suggest that treatment A is not only superior but also non‐inferior to treatment C.

### 4.2 A Hypothetical 4‐Period Dataset Based on the TALC Trial

This dataset is derived from the original TALC dataset, with the only modification being the addition of a fourth period in each sequence. Specifically, we extended the original six‐sequence, three‐period crossover design by constructing a fourth period as a replicate of the third period. This yielded the following six sequences: ABCC, ACBB, BACC, BCAA, CABB, and CBAA. For participants with non‐missing data in the third period, a value for the fourth period was generated by adding a random component to their third‐period value:

1. FVC(period4) = FVC(period3) + $N(0.035,0.07^{2})$
2. FEV1(period4) = FEV1(period3) + $N(0.050,0.10^{2})$
3. PEFR(period4) = PEFR(period3) + $N(-0.035,0.07^{2})$
4. ${\mathrm{FEF}}_{25-75}$(period4) = ${\mathrm{FEF}}_{25-75}$(period3) + $N(-0.05,0.10^{2})$

The 4‐period dataset is uniform within periods but not within sequences, and is strongly balanced. As a result, treatment effects are aliased with sequence effects (necessitating the assumption that sequence effects are null), but are not aliased with differential carryover effects. Accordingly, we apply the model specified in Equation (6) to the 4‐period dataset. As in the previous analysis, study center is included as a covariate in the model.

With the 4‐period design, we are now able to estimate the intra‐subject variance components. The REML estimation procedure is implemented using the PROC MIXED procedure in SAS. The REML estimates of the treatment means and the differential carryover effects are presented in Table 7.

**TABLE 7:** Estimate (standard error) of treatment means and carryover effects.

	FVC	FEV1	PEFR	FEF2575
$\mu_{A}$	0.23 (0.12)	0.31 (0.10)	0.92 (0.34)	0.47 (0.14)
$\mu_{B}$	0.17 (0.12)	0.21 (0.10)	0.58 (0.33)	0.33 (0.14)
$\mu_{C}$	0.20 (0.12)	0.27 (0.10)	0.82 (0.33)	0.41 (0.14)
$\lambda_{A}$	−0.02 (0.02)	0.00 (0.02)	−0.06 (0.06)	−0.03 (0.03)
$\lambda_{B}$	−0.03 (0.02)	−0.01 (0.02)	−0.01 (0.06)	−0.04 (0.03)
$\lambda_{C}$	−0.01 (0.02)	−0.01 (0.02)	−0.02 (0.06)	−0.05 (0.03)

For ease of presentation, we do not report the REML estimates of the remaining 18 nuisance parameter vectors or the 9 coefficient vectors associated with the study center covariate. The REML estimates of the inter‐subject variance‐covariance matrix Ω and the intra‐subject variance‐covariance matrices $\Sigma_{A}$, $\Sigma_{B}$, and $\Sigma_{C}$ are presented below. Similar to expression (46), in expression (47), the 12 entries correspond to the four outcomes measured under each of the three treatments (A, B, and C). Accordingly, the estimated 12×12 variance‐covariance matrix can be partitioned to nine 4×4 matrices, as shown in Figure B2. A detailed illustration of the block structure and an explicit interpretation of individual entries are provided in the Appendix B. Expressions (48) to (50) present the estimated intra‐subject variance–covariance matrices, with the 4 entries within each expression corresponding to the four outcomes. As is typically observed in crossover studies, the estimated intra‐subject variation is substantially smaller than the inter‐subject variation.

(47) $$\begin{array}{ccc} & & \;\;\;\;{\hat{\Omega }}_{\mathrm{REML}}\\ & & \;\;\;\;=\left[\begin{array}{cccccccccccc} 0.07 & 0.05 & 0.13 & 0.02 & 0.03 & 0.02 & 0.04 & 0.01 & 0.04 & 0.03 & 0.06 & 0.02\\ 0.05 & 0.05 & 0.12 & 0.05 & 0.02 & 0.02 & 0.03 & 0.02 & 0.03 & 0.03 & 0.04 & 0.03\\ 0.13 & 0.12 & 0.70 & 0.11 & 0.04 & 0.05 & 0.26 & 0.03 & 0.06 & 0.06 & 0.30 & 0.08\\ 0.02 & 0.05 & 0.11 & 0.11 & 0.01 & 0.02 & 0.02 & 0.04 & 0.02 & 0.03 & 0.04 & 0.07\\ 0.03 & 0.02 & 0.04 & 0.01 & 0.06 & 0.03 & 0.07 & 0.01 & 0.02 & 0.02 & 0.03 & 0.01\\ 0.02 & 0.02 & 0.05 & 0.02 & 0.03 & 0.03 & 0.07 & 0.04 & 0.02 & 0.02 & 0.05 & 0.03\\ 0.04 & 0.03 & 0.26 & 0.02 & 0.07 & 0.07 & 0.44 & 0.04 & 0.02 & 0.03 & 0.24 & 0.03\\ 0.01 & 0.02 & 0.03 & 0.04 & 0.01 & 0.04 & 0.04 & 0.09 & 0.02 & 0.03 & 0.06 & 0.04\\ 0.04 & 0.03 & 0.06 & 0.02 & 0.02 & 0.02 & 0.02 & 0.02 & 0.06 & 0.04 & 0.09 & 0.03\\ 0.03 & 0.03 & 0.06 & 0.03 & 0.02 & 0.02 & 0.03 & 0.03 & 0.04 & 0.05 & 0.10 & 0.06\\ 0.06 & 0.04 & 0.30 & 0.04 & 0.03 & 0.05 & 0.24 & 0.06 & 0.09 & 0.10 & 0.57 & 0.12\\ 0.02 & 0.03 & 0.08 & 0.07 & 0.01 & 0.03 & 0.03 & 0.04 & 0.03 & 0.06 & 0.12 & 0.11 \end{array}\right]\;\;\; \end{array}$$

(48) $${\hat{\Sigma }}_{A,\mathrm{REML}}=\left[\begin{array}{cccc} 0.0018 & 0.0003 & -0.0001 & 0.0002\\ 0.0003 & 0.0027 & -0.0002 & 0.0002\\ -0.0001 & -0.0002 & 0.0018 & 0.0000\\ 0.0002 & 0.0002 & 0.0000 & 0.0046 \end{array}\right]$$

(49) $${\hat{\Sigma }}_{B,\mathrm{REML}}=\left[\begin{array}{cccc} 0.0026 & 0.0005 & 0.0002 & 0.0000\\ 0.0005 & 0.0037 & 0.0004 & 0.0005\\ 0.0002 & 0.0004 & 0.0033 & -0.0001\\ 0.0000 & 0.0005 & -0.0001 & 0.0050 \end{array}\right]$$

(50) $${\hat{\Sigma }}_{C,\mathrm{REML}}=\left[\begin{array}{cccc} 0.0025 & 0.0006 & 0.0000 & 0.0005\\ 0.0006 & 0.0049 & -0.0002 & -0.0005\\ 0.0000 & -0.0002 & 0.0026 & -0.0004\\ 0.0005 & -0.0005 & -0.0004 & 0.0053 \end{array}\right]$$

Using the ESTIMATE statements in PROC MIXED, we obtain the estimates, standard errors, and t‐test results for the pairwise treatment differences for each univariate outcome. The results are presented in Table 8. For each of the four outcome variables, the point estimate of the mean difference between treatment A and treatment B is positive and statistically significant. Similarly, for each outcome, the point estimate of the mean difference between treatment A and treatment C is also positive; however, statistical significance is observed only for FEV1.

**TABLE 8:** Estimates of pairwise treatment differences for each univariate outcome.

Label	Estimate	SE	DF	t value	Pr >|t|	Lower	Upper
FVC: Treatment A versus B	0.06	0.02	248	2.81	0.0053	0.02	0.11
FVC: Treatment A versus C	0.03	0.02	268	1.27	0.2054	−0.01	0.07
FVC: Treatment B versus C	−0.04	0.02	235	−1.58	0.1152	−0.08	0.01
FEV1: Treatment A versus B	0.10	0.02	245	5.54	<0.0001	0.07	0.14
FEV1: Treatment A versus C	0.04	0.02	257	2.10	0.0366	0.00	0.08
FEV1: Treatment B versus C	−0.06	0.02	245	−3.46	0.0006	−0.10	−0.03
PEFR: Treatment A versus B	0.34	0.07	246	5.05	<0.0001	0.21	0.48
PEFR: Treatment A versus C	0.10	0.07	254	1.36	0.1762	−0.04	0.24
PEFR: Treatment B versus C	−0.25	0.06	252	−3.92	0.0001	−0.37	−0.12
FEF2575: Treatment A versus B	0.14	0.03	236	4.56	<0.0001	0.08	0.20
FEF2575: Treatment A versus C	0.05	0.03	266	1.89	0.0600	−0.00	0.11
FEF2575: Treatment B versus C	−0.08	0.03	240	−2.92	0.0039	−0.14	−0.03

We assess covariate effects, compare treatment effects, and test differences in intra‐subject variance‐covariance matrices by fitting full and nested models using the ML estimation method and constructing likelihood ratio tests. The results are summarized in Table 9. The effect of study center is not statistically significant in the model (p=0.28). The effect of treatment A is significantly different from that of treatment B (p<0.0001). As shown in Table 8, for each outcome variable, the estimated mean difference between treatment A and treatment B is positive, indicating that treatment A is superior to treatment B.

**TABLE 9:** Likelihood ratio tests of covariate effects, treatment effects, and intra‐subject variance matrices.

$H_{0}$	−2 log‐likeli (reduced)	−2 log‐likeli (full)	LR statistic	df	p‐value
$\beta_{\text{center}}=0$	−1983.0	−2023.4	40.46	36	0.2799
$\mu_{A}=\mu_{B}$	−1986.5	−2023.4	36.88	4	<0.0001
$\mu_{A}=\mu_{C}$	−2018.6	−2023.4	4.85	4	0.3027
$\mu_{B}=\mu_{C}$	−2003.5	−2023.4	19.93	4	0.0005
$\mu_{A}=\mu_{B}=\mu_{C}$	−1979.9	−2023.4	43.54	8	<0.0001
$\Sigma_{A}=\Sigma_{B}=\Sigma_{C}$	−2002.2	−2023.4	21.18	20	0.3867

In contrast, the effect of treatment A is not significantly different from that of treatment C (p=0.30). In the original study by Peters et al. (2010), the primary outcome was the change in morning peak expiratory flow rate (PEFR) from baseline (measured in liters per minute), and the objective was to assess whether treatment A was non‐inferior to treatment C. The non‐inferiority margin was defined as 10.6 L/min. In our analysis, PEFR is measured in liters per second, so we convert the non‐inferiority margin to Ψ=0.18 L/s. The lower 97.5% confidence limit for the difference in mean change in PEFR from baseline between treatment A and treatment C is −0.04, which lies within the interval (−Ψ,+∞), indicating that treatment A is non‐inferior to treatment C with respect to PEFR.

The comparison between the full model and a reduced model assuming a common intra‐subject variance‐covariance matrix across treatments indicates that there is insufficient evidence to support the need for heterogeneous intra‐subject variance‐covariance matrices in the model (p=0.39).

## 5 Discussion

In this study, we introduce a class of multivariate models and develop inference procedures tailored for general crossover trials involving t treatments and q continuous outcomes. Our modeling framework is characterized by three main features: (1) a saturated parameterization that includes the maximum number of nuisance location parameters; (2) treatment‐specific q×q intra‐subject variance‐covariance matrices; and (3) a full qt×qt inter‐subject variance‐covariance matrix. The models also accommodate the inclusion of covariates, enabling adjustment for potential confounders.

Parameter estimation is performed using iterative procedures developed for mixed‐effects linear models (Jennrich and Schluchter 1986; Laird and Ware 1982; Ware 1985), which naturally extend to the multivariate outcome setting. Based on results from Equations (14) and (15), the maximum likelihood (ML) and restricted maximum likelihood (REML) estimators of the fixed effects are computed using weights derived from the corresponding variance component estimates. In our data analysis, we rely on REML estimation due to its reduced bias in estimating variance components compared to ML. For hypothesis testing regarding treatment effects, covariate effects, and intra‐subject variance components, we employ ML estimation and construct likelihood ratio tests.

When covariates are excluded and the design is uniform within sequences, closed‐form expressions for both ML and REML estimators of the variance components are available. Furthermore, if each treatment is replicated at least twice within a sequence, then all intra‐ and inter‐subject variance components become identifiable. Under these conditions, the structure of the ML and REML estimators is described in Equations (23)–(26), and their differences tend to diminish as the total sample size n increases.

Our proposed method offers several advantages. First, the multivariate linear mixed‐effects model allows for separate modeling of intra‐ and inter‐subject variability and accommodates heterogeneity in intra‐subject variance across treatments. This feature is especially useful in applications such as bioequivalence studies, where comparing intra‐subject variability is critical. In contrast to the general multivariate model introduced by Grender and Johnson (1993, 1992), our approach can incorporate all available participant data, even in the presence of missing outcome values. Second, we propose a general framework that is applicable to a broad range of crossover designs. Third, the methodology is readily implementable using standard statistical software, such as the PROC MIXED procedure in SAS. Finally, simulation studies demonstrate that the proposed multivariate testing procedure consistently achieves strong performance and superior statistical power relative to the univariate approach across a variety of scenarios.

A limitation of our framework is its current restriction to continuous multivariate outcomes and reliance on the assumption of multivariate normality. The model's performance under violations of this assumption, such as with heavy‐tailed distributions, remains an open question. We are currently investigating methodological extensions to address these situations. And in this work, we adopt the simple first‐order carry‐over model as a pragmatic and widely used working model for cross‐over trials. As noted by Senn (2002), such a model may not be particularly grounded in biological or pharmacological reality, and there would be potential efficiency loss relative to models that ignore carry‐over. Also, there is no guarantee that a simple carry‐over specification will adequately account for more elaborate forms of carry‐over (for example, in Sheiner et al. (1989)). At the same time, we note that another alternative to simple carryover is complex carryover. However, estimation with the complex carryover model requires repeated measurements within each period (Cruz et al. 2024). Extending the proposed framework to accommodate more complex carry‐over structures remains a direction for future research.

In addition to its analytic strengths, our approach offers practical guidance for the design of crossover trials. Trial designs should align with the study's primary objectives and anticipated sources of nuisance variation. For instance, if assessing intra‐subject variability is a key objective, designs should include treatment replication within sequences. To mitigate potential carryover effects, balanced or strongly balanced designs are recommended. Uniformity across periods is generally desirable. Moreover, our closed‐form estimators and exact test results suggest that uniformity within sequences is preferable to designs lacking this property, as it enables more straightforward sample size and power calculations.

## Conflicts of Interest

The authors declare no conflicts of interest.

## Open Research Badges

This article has earned an Open Data badge for making publicly available the digitally‐shareable data necessary to reproduce the reported results. The data is available in the Supporting Information section.

This article has earned an open data badge “**Reproducible Research**” for making publicly available the code necessary to reproduce the reported results. The results reported in this article could fully be reproduced.

## Supporting information


**Supporting File:** bimj70193‐sup‐0001‐SuppMat.zip.

## Figures and Tables

**FIGURE B1:**
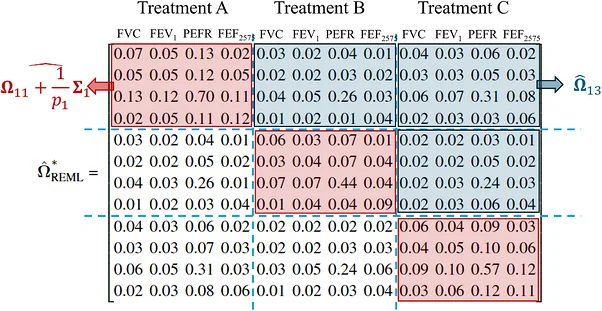
Illustration of the block‐matrix structure of the covariance matrix estimate in Expression (46).

**FIGURE B2:**
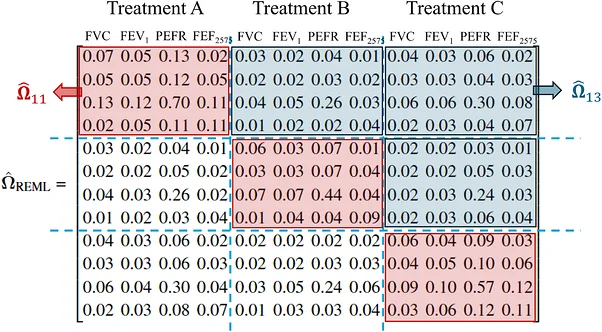
Illustration of the block‐matrix structure of the covariance matrix estimate in Expression (47).

## Data Availability

The de‐identified TALC dataset is available to the public at the Biologic Specimen and Data Repository Information Coordinating Center (BioLINCC) web site of the National Heart, Lung and Blood Institute of the National Institutes of Health (https://biolincc.nhlbi.nih.gov/home/), accession number HLB00981313a.

## Appendix A Estimations for the Mixed‐Effects Models

Here are the detailed deductions of estimations in Sections 2.3.1 and 2.3.2.

### A.1 ML/REML Estimates

The log‐likelihood for data $Y_{11},\text{…},Y_{1n_{1}},\text{…}$, $Y_{s1},\text{…},Y_{sn_{s}}$ is

(A1)
$$\begin{array}{c} l=\text{const}-\frac{1}{2}\sum_{i=1}^{s}\sum_{j=1}^{n_{i}}\log\left|\Lambda_{i}\right|-\frac{1}{2}\sum_{i=1}^{s}\sum_{j=1}^{n_{i}}{\left(Y_{ij}-X_{ij}^{*}\beta^{*}\right)}^{T}\Lambda_{i}^{-1}\left(Y_{ij}-X_{ij}^{*}\beta^{*}\right)\;\;\; \end{array}$$

Take derivative with respect to $\beta^{*}$

(A2)
$$\frac{\partial l}{\partial \beta^{*}}=\sum_{i=1}^{s}\sum_{j=1}^{n_{i}}\left[X_{ij}^{*T}\Lambda_{i}^{-1}Y_{ij}-X_{ij}^{*}\Lambda_{i}^{-1}X_{ij}^{*}\beta^{*}\right]$$

By setting it to zero, we obtain the estimate of $\beta^{*}$ given the variance components.

(A3)
$${\hat{\beta }}_{\mathrm{ML}}^{*}\left|\Lambda_{i}={\left(\sum_{i=1}^{s}\sum_{j=1}^{n_{i}}X_{ij}^{*T}\Lambda_{i}^{-1}X_{ij}^{*}\right)}^{-1}\right)\left(\sum_{i=1}^{s}\sum_{j=1}^{n_{i}}X_{ij}^{*T}\Lambda_{i}^{-1}Y_{ij}\right)$$

However, the variance components are typically unknown. Using the Fisher scoring algorithm, we iteratively update ${\hat{\beta }}^{*}|{\hat{\Lambda }}_{i}$ and ${\hat{\Lambda }}_{i}|{\hat{\beta }}^{*}$ until convergence Jennrich and Schluchter (1986). The equations for updating ${\hat{\Lambda }}_{i}$ are obtained as follows.

Collect all the variance and covariance components in the model into a vector θ. Then, $\Lambda_{i}$ for i=1,…,s are known functions of the unknown covariance parameters contained in θ.

In the model, $\Omega_{11},\Omega_{12},\text{…},\Omega_{tt},\Sigma_{1},\text{…},\Sigma_{t}$ are q×q positive definite matrices. Therefore, the total number of unknown parameters in θ is $\left[\frac{t(t+1)}{2}+t\right]\cdot \frac{q(q+1)}{2}$.

Log‐likelihood function:

(A4)
$$\begin{array}{ccc} l(\beta^{*},\theta ) & = & \text{const}-\frac{1}{2}\sum_{i=1}^{s}\sum_{j=1}^{n_{i}}\log\left|\Lambda_{i}\left(\theta \right)\right|\\ & & -\frac{1}{2}\sum_{i=1}^{s}\sum_{j=1}^{n_{i}}{\left(Y_{ij}-X_{ij}^{*}\beta^{*}\right)}^{T}\Lambda_{i}{\left(\theta \right)}^{-1}\left(Y_{ij}-X_{ij}^{*}\beta^{*}\right) \end{array}$$

Score function for θ: Suppose $\theta_{k}$ is the $k^{th}$ element in θ. Then the $k^{th}$ element of the score function vector $u_{\theta }$ is:

(A5)
$$\begin{array}{cc} u_{\theta }{\left(\beta^{*},\theta \right)}_{k} & =\frac{\partial }{\partial \theta_{k}}l\left(\beta^{*},\theta \right)\\ & =\frac{1}{2}\sum_{i=1}^{s}\sum_{j=1}^{n_{i}}[{\left(Y_{ij}-X_{ij}^{*}\beta^{*}\right)}^{T}\Lambda_{i}^{-1}\frac{\partial \Lambda_{i}}{\partial \theta_{k}}\Lambda_{i}^{-1}\left(Y_{ij}-X_{ij}^{*}\beta^{*}\right)\\ & \;-\mathrm{trace}(\Lambda_{i}^{-1}\frac{\partial \Lambda_{i}}{\partial \theta_{k}})] \end{array}$$

Set $u_{\theta }\left(\beta^{*},\theta \right)=0$ and replace $\beta^{*}$ with ${\hat{\beta }}_{\mathrm{ML}}^{*}$ in the equation. Then, ${\hat{\Lambda }}_{i,\mathrm{ML}}$ can be obtained by iteratively solving $u_{\theta }\left({\hat{\beta }}_{\mathrm{ML}}^{*},\theta \right)=0$.

Restricted log‐likelihood function:

(A6)
$$l_{R}\left(\theta \right)=l\left({\hat{\beta }}_{\mathrm{REML}}^{*},\theta \right)-\frac{1}{2}\log\left|\sum_{i=1}^{s}\sum_{j=1}^{n_{i}}X_{ij}^{*T}\Lambda_{i}^{-1}X_{ij}^{*}\right|$$

Corresponding score function for θ: Suppose $\theta_{k}$ is the $k^{th}$ element in θ. Then the $k^{th}$ component of the score vector $u_{R}{\left(\theta \right)}_{k}$ is given by:

(A7)
$$u_{R}{\left(\theta \right)}_{k}=\frac{\partial }{\partial \theta_{k}}l_{R}\left(\theta \right)=\frac{\partial }{\partial \theta_{k}}l\left({\hat{\beta }}^{*},\theta \right)-\frac{1}{2}\frac{\partial }{\partial \theta_{k}}\log\left|\sum_{i=1}^{s}\sum_{j=1}^{n_{i}}X_{ij}^{*T}\Lambda_{i}^{-1}X_{ij}^{*}\right|$$

We then obtain ${\hat{\Lambda }}_{i,\mathrm{REML}}$ by iteratively solving $u_{R}\left(\theta \right)=0$.

### A.2 Special Case With Closed‐Form Solutions

Here are the detailed deductions of the estimations $\Sigma_{k}$ (k=1,2,…,t). Since

(A8)
$$\text{var}\left[\begin{array}{c} Y_{ij1}^{*}\\ \vdots \\ Y_{ijt}^{*} \end{array}\right]=\left[\begin{array}{ccccc} & I_{p_{1}-1}⊗\Sigma_{1} & 0 & \cdots & 0\\ & 0 & I_{p_{2}-1}⊗\Sigma_{2} & \cdots & 0\\ & \vdots & \vdots & \ddots & \vdots \\ & 0 & 0 & \cdots & I_{p_{t}-1}⊗\Sigma_{t} \end{array}\right]$$

the estimation of $\Sigma_{k}$ is only related to $Y_{ijk}^{*}$’s (i=1,2,…,s and $j=1,2,\text{…},n_{i}$). The variances and expectations of $Y_{ijk}^{*}$’s are as follows:

(A9)
$$\text{var}\left(Y_{ijk}^{*}\right)=\text{var}\left[\begin{array}{c} Y_{ijk,1}^{*}\\ \vdots \\ Y_{ijk,p_{k}-1}^{*} \end{array}\right]=\left[\begin{array}{cccc} & \Sigma_{k} & \cdots & 0\\ & \vdots & \ddots & \vdots \\ & 0 & \cdots & \Sigma_{k} \end{array}\right]$$

(A10)
$$E\left(Y_{ijk}^{*}\right)=\nu_{ik}^{\left(1\right)}\;\text{or}=\nu_{ik}^{\left(2\right)}\;\text{or}=\nu_{ik}^{\left(3\right)}$$

The expectations of the $Y_{ijk}^{*}$’s are determined by the choice of model; however, the likelihood‐based estimators of $\Sigma_{k}$ do not depend on the specific model. Therefore, if we now adopt the model in Equation (1).

(A11)
$$E\left(Y_{ijk}^{*}\right)=E\left[\begin{array}{c} Y_{ijk,1}^{*}\\ \vdots \\ Y_{ijk,p_{k}-1}^{*} \end{array}\right]=\nu_{ik}^{\left(1\right)}=\left[\begin{array}{c} \nu_{ik,1}^{\left(1\right)}\\ \vdots \\ \nu_{ik,p_{k}-1}^{\left(1\right)} \end{array}\right]$$

As discussed above, since the $Y_{ijk,l}^{*}$’s ($l=1,2,\text{…},p_{k}-1$) are linear functions of the $Y_{ij}$’s, each $Y_{ijk,l}^{*}$ follows a q‐variate normal distribution:

(A12)
$$Y_{ijk,l}^{*}∼N_{q}\left(\nu_{ik,l}^{\left(1\right)},\Sigma_{k}\right)$$

From Equation (A9), the covariance matrix of $Y_{ijk,l}^{*}$ and $Y_{ijk,l^{\prime }}^{*}$ ($l,l^{\prime }=1,2,\text{…},p_{k}-1$, $l\neq l^{\prime }$) is **0**. So $Y_{ijk,1}^{*}$, $Y_{ijk,2}^{*}$, …, $Y_{ijk,p_{k}-1}^{*}$ are independent. The likelihood function is:

(A13)
$$\begin{array}{ccc} L & = & \prod_{i=1}^{s}\prod_{j=1}^{n_{i}}\prod_{l=1}^{p_{k}-1}{\left(2\pi \right)}^{-q/2}\det{\left(\Sigma_{k}\right)}^{-1/2}\\ & & \times \exp\left[-\frac{1}{2}{\left(Y_{ijk,l}^{*}-\nu_{ik,l}^{\left(1\right)}\right)}^{T}\Sigma_{k}^{-1}\left(Y_{ijk,l}^{*}-\nu_{ik,l}^{\left(1\right)}\right)\right] \end{array}$$

The log‐likelihood function is:

(A14)
$$\begin{array}{ccc} l & = & \sum_{i=1}^{s}\sum_{j=1}^{n_{i}}\sum_{l=1}^{p_{k}-1}\left[-\frac{1}{2}\log\left(|\Sigma_{k}|\right)\right]\\ & & -\frac{1}{2}\sum_{i=1}^{s}\sum_{j=1}^{n_{i}}\sum_{l=1}^{p_{k}-1}{\left(Y_{ijk,l}^{*}-\nu_{ik,l}^{\left(1\right)}\right)}^{T}\Sigma_{k}^{-1}\left(Y_{ijk,l}^{*}-\nu_{ik,l}^{\left(1\right)}\right)+\mathrm{const}\; \end{array}$$

The maximum likelihood estimator of $\Sigma_{k}$ is:

(A15)
$${\hat{\Sigma }}_{k,\mathrm{ML}}=\frac{1}{n(p_{k}-1)}\sum_{i=1}^{s}\sum_{j=1}^{n_{i}}\sum_{l=1}^{p_{k}-1}\left(Y_{ijk,l}^{*}-{\bar{Y}}_{i\cdot k,l}^{*}\right){\left(Y_{ijk,l}^{*}-{\bar{Y}}_{i\cdot k,l}^{*}\right)}^{T}$$

where ${\bar{Y}}_{i\cdot k,l}^{*}=\frac{1}{n_{i}}\sum_{j=1}^{n_{i}}Y_{ijk,l}^{*}$. The restricted log‐likelihood function is:

(A16)
$$l_{R}=l-\frac{1}{2}\log\left[\det\sum_{i=1}^{s}\sum_{j=1}^{n_{i}}\sum_{l=1}^{p_{k}-1}Z_{ijkl}^{T}\Sigma_{k}^{-1}Z_{ijkl}\right]$$

where l is the likelihood function in Equation (A14), $Z_{ijkl}$ is the indicator matrix for the lth orthogonal contrast of kth treatment for jth subject in ith sequence corresponding to the mean vector:

(A17)
$$\nu =\left[\begin{array}{c} \nu_{1k,1}\\ \vdots \\ \nu_{1k,p_{k}-1}\\ \vdots \\ \nu_{sk,1}\\ \vdots \\ \nu_{sk,p_{k}-1} \end{array}\right]$$

For example, for any subject in the first sequence, the matrix $Z_{ijkl}$ corresponding to the first orthogonal contrast of the kth treatment is given by $Z_{ijkl}=\left[I_{q\times q}\;0\;\cdots \;0\right]$. Based on the restricted likelihood, the restricted maximum likelihood (REML) estimator of $\Sigma_{k}$ for k=1,2,…,t is:

(A18)
$${\hat{\Sigma }}_{k,\mathrm{REML}}=\frac{1}{\left(n-s\right)\left(p_{k}-1\right)}\sum_{i=1}^{s}\sum_{j=1}^{n_{i}}\sum_{l=1}^{p_{k}-1}\left(Y_{ijk,l}^{*}-{\bar{Y}}_{i\cdot k,l}^{*}\right){\left(Y_{ijk,l}^{*}-{\bar{Y}}_{i\cdot k,l}^{*}\right)}^{T}$$

## Appendix B Explanation for the Estimates of Variance‐Covariance Matrices in the Data Application

Figure B1 illustrates the block‐matrix structure of the estimated variance‐covariance matrix in Expression (46). The matrix is partitioned by treatment (A, B, and C). Within each block, the 4 entries correspond to the four outcomes: FVC, FEV1, PEFR, ${\mathrm{FEF}}_{25-75}$. The diagonal blocks (highlighted in red) correspond to within‐treatment variance‐covariance matrices, each consisting of the inseparable between‐subject variance component and the within‐subject variance component (as defined in Equation 19). The off‐diagonal blocks (highlighted in blue) represent cross‐treatment covariance components, capturing the variance‐covariance matrices of the random effect vectors of different treatments.

Figure B2 presents the block‐matrix structure of the estimated variance‐covariance matrix in Expression (47). Similarly, the matrix is partitioned by treatment (A, B and C). In contrast to Figure B1, the diagonal blocks (highlighted in red) now comprise only the between‐subject variability and correspond to the variance‐covariance matrices of random‐effect vectors within the same treatment. The off‐diagonal blocks (highlighted in blue) continue to represent cross‐treatment covariance components, capturing the variance‐covariance relationships between random‐effect vectors from different treatments.
