# Supplementary material for: Multivariate Analysis of Data From Crossover Clinical Trials
Source: Biom J. 2026 Oct 11;68(5):e70193. doi: 10.1002/bimj.70193 (PMC13655226; doi:10.1002/bimj.70193)
Supplement: Supplementary file 1 [file BIMJ-68-e70193-s001.zip › code_and_data_supplement/Data_Application/SAS_output_results.pdf]

Multivariate Analysis of FVC, FEV1, PEFR, and FEF2575 with Center as covariate

The Mixed Procedure

Model Information	
**Data Set**	WORK.TALC_3PERIOD
**Dependent Variable**	outcome
**Covariance Structure**	Unstructured
**Subject Effect**	participant
**Estimation Method**	REML
**Residual Variance Method**	None
**Fixed Effects SE Method**	Model-Based
**Degrees of Freedom Method**	Satterthwaite

Class Level Information

Class	Levels	Values
**participant**	187	1 2 3 5 7 8 10 11 12 13 14 15 16 17 18 19 20 21 22 23 24 25 26 27 28 29 30 31 32 33 35 36 37 38 39 41 42 43 44 45 47 48 50 52 53 54 55 56 57 58 59 60 61 62 63 65 66 67 68 69 70 71 72 73 74 75 76 77 78 79 80 81 82 83 84 85 86 87 88 91 92 93 94 95 96 97 98 99 100 101 102 103 104 105 106 108 109 110 111 113 114 116 117 118 119 120 121 122 123 124 125 126 127 128 129 130 131 132 133 134 135 136 137 139 140 141 142 143 144 145 146 147 148 149 150 151 152 153 154 155 156 157 158 159 160 161 163 164 165 167 168 170 171 173 174 175 176 179 180 181 182 183 184 185 186 187 188 189 190 191 192 193 194 196 197 198 199 200 201 202 203 204 206 207 208 209 210
**variable**	4	FVC FEV1 PEFR FEF2575
**sequence**	6	ABC ACB BAC BCA CAB CBA
**period**	3	1 2 3
**treatment**	3	Tiotropium Double-glucocorticoid Salmeterol

Dimensions	
**Covariance Parameters**	78
**Columns in X**	108
**Columns in Z**	0
**Subjects**	187
**Max Obs per Subject**	12

Number of Observations	
**Number of Observations Read**	2244
**Number of Observations Used**	2244
**Number of Observations Not Used**	0

Iteration History			
**Iteration**	**Evaluations**	**-2 Res Log Like**	**Criterion**
**0**	1	2915.33586104	
**1**	2	-361.21207274	0.00000262
**2**	1	-361.21773775	0.00000000

Convergence criteria met.

Covariance Parameter Estimates		
**Cov Parm**	**Subject**	**Estimate**
**UN(1,1)**	participant	0.06099
**UN(2,1)**	participant	0.04091
**UN(2,2)**	participant	0.04761
**UN(3,1)**	participant	0.1211
**UN(3,2)**	participant	0.1073
**UN(3,3)**	participant	0.6289
**UN(4,1)**	participant	0.01679
**UN(4,2)**	participant	0.05232
**UN(4,3)**	participant	0.08126
**UN(4,4)**	participant	0.1215
**UN(5,1)**	participant	0.02390
**UN(5,2)**	participant	0.01593
**UN(5,3)**	participant	0.03534
**UN(5,4)**	participant	0.01470
**UN(5,5)**	participant	0.05738
**UN(6,1)**	participant	0.01844
**UN(6,2)**	participant	0.01958
**UN(6,3)**	participant	0.03332
**UN(6,4)**	participant	0.02089
**UN(6,5)**	participant	0.03210
**UN(6,6)**	participant	0.03307
**UN(7,1)**	participant	0.04608
**UN(7,2)**	participant	0.02818
**UN(7,3)**	participant	0.2496
**UN(7,4)**	participant	0.009479

**UN(7,5)**	participant	0.08935
**UN(7,6)**	participant	0.06509
**UN(7,7)**	participant	0.4609
**UN(8,1)**	participant	0.007367
**UN(8,2)**	participant	0.02265
**UN(8,3)**	participant	0.01457
**UN(8,4)**	participant	0.03634
**UN(8,5)**	participant	0.001118
**UN(8,6)**	participant	0.03074
**UN(8,7)**	participant	0.006535
**UN(8,8)**	participant	0.07471
**UN(9,1)**	participant	0.02874
**UN(9,2)**	participant	0.02428
**UN(9,3)**	participant	0.04392
**UN(9,4)**	participant	0.01661
**UN(9,5)**	participant	0.01860
**UN(9,6)**	participant	0.01689
**UN(9,7)**	participant	0.02486
**UN(9,8)**	participant	0.01285
**UN(9,9)**	participant	0.05062
**UN(10,1)**	participant	0.02191
**UN(10,2)**	participant	0.02727
**UN(10,3)**	participant	0.05436
**UN(10,4)**	participant	0.02962
**UN(10,5)**	participant	0.01572
**UN(10,6)**	participant	0.02106
**UN(10,7)**	participant	0.02988
**UN(10,8)**	participant	0.02376
**UN(10,9)**	participant	0.03863
**UN(10,10)**	participant	0.04890
**UN(11,1)**	participant	0.03388
**UN(11,2)**	participant	0.02024
**UN(11,3)**	participant	0.2240
**UN(11,4)**	participant	-0.00443
**UN(11,5)**	participant	0.03108

	participant	0.04018
UN(11,7)	participant	0.2287
UN(11,8)	participant	0.03740
UN(11,9)	participant	0.06554
UN(11,10)	participant	0.09785
UN(11,11)	participant	0.5706
UN(12,1)	participant	0.01564
UN(12,2)	participant	0.03607
UN(12,3)	participant	0.06565
UN(12,4)	participant	0.06885
UN(12,5)	participant	0.01636
UN(12,6)	participant	0.02817
UN(12,7)	participant	0.02398
UN(12,8)	participant	0.04277
UN(12,9)	participant	0.03121
UN(12,10)	participant	0.06462
UN(12,11)	participant	0.1268
UN(12,12)	participant	0.1284

Fit Statistics	
-2 Res Log Likelihood	-361.2
AIC (Smaller is Better)	-205.2
AICC (Smaller is Better)	-199.2
BIC (Smaller is Better)	46.8

Null Model Likelihood Ratio Test		
DF	Chi-Square	Pr > ChiSq
77	3276.55	<.0001

Solution for Fixed Effects

Effect	variable	Estimate	Standard Error	DF	t Value	Pr > |t|
mu_a(variable)	FVC	0.2099	0.1041	181	2.02	0.0451
mu_a(variable)	FEV1	0.3287	0.09390	180	3.50	0.0006
mu_a(variable)	PEFR	0.9678	0.3190	182	3.03	0.0028
mu_a(variable)	FEF2575	0.5344	0.1387	182	3.85	0.0002

**mu_b(variable)**	FVC	0.1407	0.1040	180	1.35	0.1779
**mu_b(variable)**	FEV1	0.2290	0.09352	177	2.45	0.0153
**mu_b(variable)**	PEFR	0.6006	0.3178	180	1.89	0.0604
**mu_b(variable)**	FEF2575	0.4015	0.1379	178	2.91	0.0041
**mu_c(variable)**	FVC	0.1630	0.1046	180	1.56	0.1209
**mu_c(variable)**	FEV1	0.2656	0.09477	182	2.80	0.0056
**mu_c(variable)**	PEFR	0.7744	0.3216	184	2.41	0.0170
**mu_c(variable)**	FEF2575	0.4617	0.1403	186	3.29	0.0012
**lambda(variable)**	FVC	-0.06059	0.01687	387	-3.59	0.0004
**lambda(variable)**	FEV1	-0.03608	0.01414	353	-2.55	0.0112
**lambda(variable)**	PEFR	0.009259	0.05512	332	0.17	0.8667
**lambda(variable)**	FEF2575	-0.01976	0.02355	356	-0.84	0.4021
**gamma_1a(variable)**	FVC	-0.03173	0.03935	187	-0.81	0.4211
**gamma_1a(variable)**	FEV1	-0.03275	0.03529	187	-0.93	0.3547
**gamma_1a(variable)**	PEFR	0.03271	0.1240	187	0.26	0.7922
**gamma_1a(variable)**	FEF2575	-0.02919	0.05613	190	-0.52	0.6036
**gamma_2a(variable)**	FVC	0.01103	0.03824	198	0.29	0.7732
**gamma_2a(variable)**	FEV1	-0.00244	0.03386	199	-0.07	0.9425
**gamma_2a(variable)**	PEFR	0.1416	0.1223	200	1.16	0.2483
**gamma_2a(variable)**	FEF2575	-0.02758	0.05382	202	-0.51	0.6089
**gamma_3a(variable)**	FVC	0.06277	0.04091	177	1.53	0.1267
**gamma_3a(variable)**	FEV1	0.07979	0.03607	180	2.21	0.0282
**gamma_3a(variable)**	PEFR	-0.00639	0.1317	178	-0.05	0.9614
**gamma_3a(variable)**	FEF2575	0.1089	0.05726	182	1.90	0.0588
**gamma_4a(variable)**	FVC	0.01726	0.04091	177	0.42	0.6736
**gamma_4a(variable)**	FEV1	-0.03680	0.03608	180	-1.02	0.3091
**gamma_4a(variable)**	PEFR	-0.2563	0.1318	178	-1.95	0.0533
**gamma_4a(variable)**	FEF2575	-0.1201	0.05727	182	-2.10	0.0374
**gamma_5a(variable)**	FVC	-0.04972	0.04121	175	-1.21	0.2292
**gamma_5a(variable)**	FEV1	-0.02236	0.03662	179	-0.61	0.5422
**gamma_5a(variable)**	PEFR	-0.1886	0.1312	177	-1.44	0.1523
**gamma_5a(variable)**	FEF2575	0.01716	0.05871	181	0.29	0.7704
**gamma_1b(variable)**	FVC	-0.06983	0.03616	177	-1.93	0.0550
**gamma_1b(variable)**	FEV1	-0.02746	0.02700	179	-1.02	0.3105
**gamma_1b(variable)**	PEFR	-0.1825	0.1020	181	-1.79	0.0752

	FEF2575	-0.04612	0.04069	178	-1.13	0.2586
**gamma_2b(variable)**	FVC	-0.01437	0.03878	177	-0.37	0.7114
**gamma_2b(variable)**	FEV1	-0.01412	0.02967	180	-0.48	0.6348
**gamma_2b(variable)**	PEFR	0.1080	0.1108	182	0.97	0.3312
**gamma_2b(variable)**	FEF2575	0.02714	0.04447	179	0.61	0.5425
**gamma_3b(variable)**	FVC	0.04570	0.03805	195	1.20	0.2311
**gamma_3b(variable)**	FEV1	0.07590	0.02993	203	2.54	0.0120
**gamma_3b(variable)**	PEFR	-0.1082	0.1111	209	-0.97	0.3312
**gamma_3b(variable)**	FEF2575	0.09957	0.04508	207	2.21	0.0283
**gamma_4b(variable)**	FVC	0.03666	0.03808	196	0.96	0.3369
**gamma_4b(variable)**	FEV1	-0.04294	0.02997	203	-1.43	0.1535
**gamma_4b(variable)**	PEFR	-0.04884	0.1113	209	-0.44	0.6611
**gamma_4b(variable)**	FEF2575	-0.1376	0.04514	207	-3.05	0.0026
**gamma_5b(variable)**	FVC	-0.1120	0.03946	177	-2.84	0.0051
**gamma_5b(variable)**	FEV1	-0.04070	0.03059	180	-1.33	0.1850
**gamma_5b(variable)**	PEFR	-0.3666	0.1132	181	-3.24	0.0014
**gamma_5b(variable)**	FEF2575	0.07802	0.04601	178	1.70	0.0917
**gamma_2c(variable)**	FVC	0.03503	0.03611	177	0.97	0.3334
**gamma_2c(variable)**	FEV1	0.01538	0.03517	174	0.44	0.6626
**gamma_2c(variable)**	PEFR	0.1547	0.1189	179	1.30	0.1949
**gamma_2c(variable)**	FEF2575	-0.00163	0.05717	177	-0.03	0.9773
**gamma_3c(variable)**	FVC	0.04551	0.03617	179	1.26	0.2099
**gamma_3c(variable)**	FEV1	0.07846	0.03553	176	2.21	0.0285
**gamma_3c(variable)**	PEFR	-0.00373	0.1220	181	-0.03	0.9757
**gamma_3c(variable)**	FEF2575	0.1528	0.05721	178	2.67	0.0083
**gamma_4c(variable)**	FVC	-0.02443	0.03621	179	-0.67	0.5008
**gamma_4c(variable)**	FEV1	-0.1038	0.03557	176	-2.92	0.0040
**gamma_4c(variable)**	PEFR	-0.3069	0.1221	181	-2.51	0.0128
**gamma_4c(variable)**	FEF2575	-0.2472	0.05726	179	-4.32	<.0001
**gamma_5c(variable)**	FVC	-0.04645	0.03608	190	-1.29	0.1995
**gamma_5c(variable)**	FEV1	-0.00154	0.03521	182	-0.04	0.9651
**gamma_5c(variable)**	PEFR	-0.1223	0.1203	190	-1.02	0.3109
**gamma_5c(variable)**	FEF2575	0.05250	0.05665	186	0.93	0.3553
**center1(variable)**	FVC	-0.1269	0.1098	172	-1.16	0.2494
**center1(variable)**	FEV1	-0.1752	0.09919	172	-1.77	0.0791

	PEFR	-0.3656	0.3358	172	-1.09	0.2778
**center1(variable)**	FEF2575	-0.2698	0.1460	172	-1.85	0.0663
**center2(variable)**	FVC	-0.1147	0.1079	172	-1.06	0.2892
**center2(variable)**	FEV1	-0.1437	0.09753	172	-1.47	0.1424
**center2(variable)**	PEFR	-0.3587	0.3302	172	-1.09	0.2788
**center2(variable)**	FEF2575	-0.2758	0.1435	172	-1.92	0.0563
**center3(variable)**	FVC	-0.1092	0.1094	172	-1.00	0.3196
**center3(variable)**	FEV1	-0.1842	0.09889	172	-1.86	0.0643
**center3(variable)**	PEFR	-0.5509	0.3348	172	-1.65	0.1017
**center3(variable)**	FEF2575	-0.3431	0.1455	172	-2.36	0.0195
**center5(variable)**	FVC	-0.1053	0.1105	172	-0.95	0.3420
**center5(variable)**	FEV1	-0.2282	0.09983	172	-2.29	0.0235
**center5(variable)**	PEFR	-0.4687	0.3380	172	-1.39	0.1672
**center5(variable)**	FEF2575	-0.4741	0.1469	172	-3.23	0.0015
**center6(variable)**	FVC	0.01965	0.1082	172	0.18	0.8562
**center6(variable)**	FEV1	-0.07733	0.09782	172	-0.79	0.4303
**center6(variable)**	PEFR	-0.4229	0.3311	172	-1.28	0.2033
**center6(variable)**	FEF2575	-0.2587	0.1440	172	-1.80	0.0740
**center7(variable)**	FVC	-0.07649	0.1167	172	-0.66	0.5132
**center7(variable)**	FEV1	-0.2006	0.1055	172	-1.90	0.0589
**center7(variable)**	PEFR	-0.3102	0.3571	172	-0.87	0.3863
**center7(variable)**	FEF2575	-0.4110	0.1553	172	-2.65	0.0089
**center8(variable)**	FVC	-0.08905	0.1067	172	-0.83	0.4053
**center8(variable)**	FEV1	-0.2389	0.09646	172	-2.48	0.0142
**center8(variable)**	PEFR	-0.5190	0.3266	172	-1.59	0.1138
**center8(variable)**	FEF2575	-0.4801	0.1420	172	-3.38	0.0009
**center9(variable)**	FVC	0.05941	0.1119	172	0.53	0.5962
**center9(variable)**	FEV1	-0.1234	0.1011	172	-1.22	0.2241
**center9(variable)**	PEFR	-0.4757	0.3424	172	-1.39	0.1665
**center9(variable)**	FEF2575	-0.4125	0.1488	172	-2.77	0.0062
**center10(variable)**	FVC	0.1527	0.1168	172	1.31	0.1926
**center10(variable)**	FEV1	0.08790	0.1055	172	0.83	0.4059
**center10(variable)**	PEFR	0.4330	0.3572	172	1.21	0.2271
**center10(variable)**	FEF2575	-0.04328	0.1553	172	-0.28	0.7808

Type 3 Tests of Fixed Effects				
Effect	Num DF	Den DF	F Value	Pr > F
mu_a(variable)	4	183	4.92	0.0009
mu_b(variable)	4	180	2.61	0.0369
mu_c(variable)	4	184	3.51	0.0087
lambda(variable)	4	364	5.00	0.0006
gamma_1a(variable)	4	188	0.52	0.7205
gamma_2a(variable)	4	199	0.60	0.6644
gamma_3a(variable)	4	179	2.08	0.0849
gamma_4a(variable)	4	179	2.69	0.0329
gamma_5a(variable)	4	178	0.72	0.5790
gamma_1b(variable)	4	179	6.07	0.0001
gamma_2b(variable)	4	180	2.51	0.0432
gamma_3b(variable)	4	206	3.84	0.0050
gamma_4b(variable)	4	207	2.96	0.0210
gamma_5b(variable)	4	179	3.98	0.0041
gamma_2c(variable)	4	179	0.81	0.5220
gamma_3c(variable)	4	181	2.41	0.0508
gamma_4c(variable)	4	181	5.24	0.0005
gamma_5c(variable)	4	191	1.50	0.2038
center1(variable)	4	172	1.02	0.3979
center2(variable)	4	172	1.50	0.2039
center3(variable)	4	172	1.87	0.1171
center5(variable)	4	172	2.94	0.0222
center6(variable)	4	172	1.59	0.1797
center7(variable)	4	172	1.79	0.1321
center8(variable)	4	172	3.09	0.0173
center9(variable)	4	172	3.02	0.0193
center10(variable)	4	172	0.75	0.5564

Estimates								
Label	Estimate	Standard Error	DF	t Value	Pr > |t|	Alpha	Lower	Upper
FVC: Treatment A vs B	0.06922	0.01948	181	3.55	0.0005	0.05	0.03079	0.1076
FVC: Treatment A vs C	0.04694	0.01841	222	2.55	0.0115	0.05	0.01066	0.08322
FVC: Treatment B vs C	-0.02228	0.02080	206	-1.07	0.2853	0.05	-0.06328	0.01872

**FEV1: Treatment A vs B**	0.09970	0.01494	181	6.67	<.0001	0.05	0.07022	0.1292
**FEV1: Treatment A vs C**	0.06317	0.01648	226	3.83	0.0002	0.05	0.03070	0.09565
**FEV1: Treatment B vs C**	-0.03653	0.01621	213	-2.25	0.0253	0.05	-0.06848	-0.00457
**PEFR: Treatment A vs B**	0.3672	0.05634	181	6.52	<.0001	0.05	0.2560	0.4783
**PEFR: Treatment A vs C**	0.1934	0.06775	210	2.85	0.0047	0.05	0.05983	0.3269
**PEFR: Treatment B vs C**	-0.1738	0.06040	210	-2.88	0.0044	0.05	-0.2928	-0.05471
**FEF2575: Treatment A vs B**	0.1329	0.02577	181	5.16	<.0001	0.05	0.08203	0.1837
**FEF2575: Treatment A vs C**	0.07265	0.02693	225	2.70	0.0075	0.05	0.01957	0.1257
**FEF2575: Treatment B vs C**	-0.06024	0.02764	205	-2.18	0.0304	0.05	-0.1147	-0.00575

Contrasts

Label	Num DF	Den DF	F Value	Pr > F
Treatment A - Treatment B	4	181	14.53	<.0001
Treatment A - Treatment C	4	223	3.80	0.0052
Treatment B - Treatment C	4	208	2.55	0.0401

Multivariate Analysis of FVC, FEV1, PEFR, and FEF2575 with no covariates

The Mixed Procedure

Model Information	
Data Set	WORK.TALC_3PERIOD
Dependent Variable	outcome
Covariance Structure	Unstructured
Subject Effect	participant
Estimation Method	ML
Residual Variance Method	None
Fixed Effects SE Method	Model-Based
Degrees of Freedom Method	Satterthwaite

Class Level Information		
**Class**	**Levels**	**Values**
participant	187	1 2 3 5 7 8 10 11 12 13 14 15 16 17 18 19 20 21 22 23 24 25 26 27 28 29 30 31 32 33 35 36 37 38 39 41 42 43 44 45 47 48 50 52 53 54 55 56 57 58 59 60 61 62 63 65 66 67 68 69 70 71 72 73 74 75 76 77 78 79 80 81 82 83 84 85 86 87 88 91 92 93 94 95 96 97 98 99 100 101 102 103 104 105 106 108 109 110 111 113 114 116 117 118 119 120 121 122 123 124 125 126 127 128 129 130 131 132 133 134 135 136 137 139 140 141 142 143 144 145 146 147 148 149 150 151 152 153 154 155 156 157 158 159 160 161 163 164 165 167 168 170 171 173 174 175 176 179 180 181 182 183 184 185 186 187 188 189 190 191 192 193 194 196 197 198 199 200 201 202 203 204 206 207 208 209 210
variable	4	FVC FEV1 PEFR FEF2575
sequence	6	ABC ACB BAC BCA CAB CBA
period	3	1 2 3
treatment	3	Tiotropium Double-glucocorticoid Salmeterol

Dimensions	
Covariance Parameters	78
Columns in X	72
Columns in Z	0
Subjects	187
Max Obs per Subject	12

Number of Observations	
Number of Observations Read	2244
Number of Observations Used	2244

Number of Observations Not Used	0

Iteration History

Iteration	Evaluations	-2 Log Like	Criterion
0	1	2714.18681185	
1	1	-811.79244478	0.00000000

Convergence criteria met.

Covariance Parameter Estimates

Cov Parm	Subject	Estimate
UN(1,1)	participant	0.06688
UN(2,1)	participant	0.02813
UN(2,2)	participant	0.05769
UN(3,1)	participant	0.03451
UN(3,2)	participant	0.02186
UN(3,3)	participant	0.05455
UN(4,1)	participant	0.04438
UN(4,2)	participant	0.01999
UN(4,3)	participant	0.02875
UN(4,4)	participant	0.05132
UN(5,1)	participant	0.02215
UN(5,2)	participant	0.03518
UN(5,3)	participant	0.02113
UN(5,4)	participant	0.02436
UN(5,5)	participant	0.03760
UN(6,1)	participant	0.02496
UN(6,2)	participant	0.01877
UN(6,3)	participant	0.04161
UN(6,4)	participant	0.03166
UN(6,5)	participant	0.02583
UN(6,6)	participant	0.05263
UN(7,1)	participant	0.1260
UN(7,2)	participant	0.04705
UN(7,3)	participant	0.05012
UN(7,4)	participant	0.1136

**UN(7,5)**	participant	0.04458
**UN(7,6)**	participant	0.05986
**UN(7,7)**	participant	0.6282
**UN(8,1)**	participant	0.05162
**UN(8,2)**	participant	0.09756
**UN(8,3)**	participant	0.02991
**UN(8,4)**	participant	0.03958
**UN(8,5)**	participant	0.07795
**UN(8,6)**	participant	0.03878
**UN(8,7)**	participant	0.2720
**UN(8,8)**	participant	0.4874
**UN(9,1)**	participant	0.03645
**UN(9,2)**	participant	0.03781
**UN(9,3)**	participant	0.06593
**UN(9,4)**	participant	0.02797
**UN(9,5)**	participant	0.04991
**UN(9,6)**	participant	0.1006
**UN(9,7)**	participant	0.2358
**UN(9,8)**	participant	0.2513
**UN(9,9)**	participant	0.5710
**UN(10,1)**	participant	0.01605
**UN(10,2)**	participant	0.01658
**UN(10,3)**	participant	0.01787
**UN(10,4)**	participant	0.05598
**UN(10,5)**	participant	0.02634
**UN(10,6)**	participant	0.03495
**UN(10,7)**	participant	0.09420
**UN(10,8)**	participant	0.03080
**UN(10,9)**	participant	0.01210
**UN(10,10)**	participant	0.1307
**UN(11,1)**	participant	0.01067
**UN(11,2)**	participant	0.007172
**UN(11,3)**	participant	0.01797
**UN(11,4)**	participant	0.02869
**UN(11,5)**	participant	0.03731

	participant	0.03070
**UN(11,7)**	participant	0.02918
**UN(11,8)**	participant	0.02747
**UN(11,9)**	participant	0.05210
**UN(11,10)**	participant	0.04668
**UN(11,11)**	participant	0.08232
**UN(12,1)**	participant	0.01635
**UN(12,2)**	participant	0.01961
**UN(12,3)**	participant	0.03342
**UN(12,4)**	participant	0.04073
**UN(12,5)**	participant	0.03386
**UN(12,6)**	participant	0.06930
**UN(12,7)**	participant	0.07396
**UN(12,8)**	participant	0.03970
**UN(12,9)**	participant	0.1340
**UN(12,10)**	participant	0.07840
**UN(12,11)**	participant	0.05166
**UN(12,12)**	participant	0.1348

Fit Statistics	
**-2 Log Likelihood**	-811.8
**AIC (Smaller is Better)**	-511.8
**AICC (Smaller is Better)**	-490.1
**BIC (Smaller is Better)**	-27.1

Null Model Likelihood Ratio Test

DF	Chi-Square	Pr > ChiSq
77	3525.98	<.0001

Solution for Fixed Effects

Effect	variable	Estimate	Standard Error	DF	t Value	Pr > |t|
**mu_a(variable)**	FVC	0.1500	0.02188	302	6.86	<.0001
**mu_a(variable)**	FEV1	0.1721	0.01906	294	9.03	<.0001
**mu_a(variable)**	PEFR	0.5771	0.06805	312	8.48	<.0001
**mu_a(variable)**	FEF2575	0.1935	0.03066	297	6.31	<.0001

**mu_b(variable)**	FVC	0.08080	0.02096	290	3.85	0.0001
**mu_b(variable)**	FEV1	0.07243	0.01723	304	4.20	<.0001
**mu_b(variable)**	PEFR	0.2099	0.06329	323	3.32	0.0010
**mu_b(variable)**	FEF2575	0.06062	0.02643	334	2.29	0.0224
**mu_c(variable)**	FVC	0.1026	0.02313	237	4.43	<.0001
**mu_c(variable)**	FEV1	0.1082	0.02229	217	4.86	<.0001
**mu_c(variable)**	PEFR	0.3822	0.07622	226	5.02	<.0001
**mu_c(variable)**	FEF2575	0.1198	0.03577	219	3.35	0.0010
**lambda(variable)**	FVC	-0.06008	0.01663	406	-3.61	0.0003
**lambda(variable)**	FEV1	-0.03537	0.01408	370	-2.51	0.0124
**lambda(variable)**	PEFR	0.01074	0.05445	347	0.20	0.8437
**lambda(variable)**	FEF2575	-0.01872	0.02331	374	-0.80	0.4224
**gamma_1a(variable)**	FVC	-0.02863	0.04137	204	-0.69	0.4896
**gamma_1a(variable)**	FEV1	-0.02849	0.03691	200	-0.77	0.4410
**gamma_1a(variable)**	PEFR	0.04161	0.1243	205	0.33	0.7383
**gamma_1a(variable)**	FEF2575	-0.02298	0.05847	201	-0.39	0.6947
**gamma_2a(variable)**	FVC	0.004936	0.03995	213	0.12	0.9018
**gamma_2a(variable)**	FEV1	-0.01044	0.03521	211	-0.30	0.7672
**gamma_2a(variable)**	PEFR	0.1171	0.1224	217	0.96	0.3395
**gamma_2a(variable)**	FEF2575	-0.03895	0.05582	213	-0.70	0.4861
**gamma_3a(variable)**	FVC	0.05743	0.04271	192	1.34	0.1803
**gamma_3a(variable)**	FEV1	0.07460	0.03743	192	1.99	0.0477
**gamma_3a(variable)**	PEFR	-0.00426	0.1315	193	-0.03	0.9742
**gamma_3a(variable)**	FEF2575	0.1031	0.05934	192	1.74	0.0840
**gamma_4a(variable)**	FVC	0.02659	0.04271	192	0.62	0.5343
**gamma_4a(variable)**	FEV1	-0.02911	0.03743	192	-0.78	0.4377
**gamma_4a(variable)**	PEFR	-0.2419	0.1315	193	-1.84	0.0675
**gamma_4a(variable)**	FEF2575	-0.1142	0.05934	192	-1.92	0.0557
**gamma_5a(variable)**	FVC	-0.04584	0.04309	191	-1.06	0.2888
**gamma_5a(variable)**	FEV1	-0.02116	0.03794	191	-0.56	0.5777
**gamma_5a(variable)**	PEFR	-0.2035	0.1308	192	-1.56	0.1214
**gamma_5a(variable)**	FEF2575	0.01597	0.06072	191	0.26	0.7928
**gamma_1b(variable)**	FVC	-0.06725	0.03618	191	-1.86	0.0646
**gamma_1b(variable)**	FEV1	-0.02392	0.02866	192	-0.83	0.4050
**gamma_1b(variable)**	PEFR	-0.1751	0.1046	193	-1.67	0.0956

	FEF2575	-0.04094	0.04257	194	-0.96	0.3373
**gamma_2b(variable)**	FVC	-0.02099	0.03893	192	-0.54	0.5905
**gamma_2b(variable)**	FEV1	-0.02282	0.03158	193	-0.72	0.4708
**gamma_2b(variable)**	PEFR	0.08198	0.1137	194	0.72	0.4716
**gamma_2b(variable)**	FEF2575	0.01474	0.04667	195	0.32	0.7525
**gamma_3b(variable)**	FVC	0.04088	0.03826	211	1.07	0.2865
**gamma_3b(variable)**	FEV1	0.07142	0.03177	213	2.25	0.0256
**gamma_3b(variable)**	PEFR	-0.1046	0.1137	220	-0.92	0.3583
**gamma_3b(variable)**	FEF2575	0.09479	0.04718	221	2.01	0.0457
**gamma_4b(variable)**	FVC	0.04650	0.03826	211	1.22	0.2256
**gamma_4b(variable)**	FEV1	-0.03454	0.03177	213	-1.09	0.2783
**gamma_4b(variable)**	PEFR	-0.03295	0.1137	220	-0.29	0.7722
**gamma_4b(variable)**	FEF2575	-0.1307	0.04718	221	-2.77	0.0061
**gamma_5b(variable)**	FVC	-0.1081	0.03952	192	-2.74	0.0068
**gamma_5b(variable)**	FEV1	-0.03949	0.03250	192	-1.22	0.2258
**gamma_5b(variable)**	PEFR	-0.3815	0.1161	193	-3.29	0.0012
**gamma_5b(variable)**	FEF2575	0.07683	0.04813	193	1.60	0.1120
**gamma_2c(variable)**	FVC	0.02893	0.03752	193	0.77	0.4416
**gamma_2c(variable)**	FEV1	0.007384	0.03644	190	0.20	0.8396
**gamma_2c(variable)**	PEFR	0.1302	0.1187	192	1.10	0.2741
**gamma_2c(variable)**	FEF2575	-0.01300	0.05856	190	-0.22	0.8246
**gamma_3c(variable)**	FVC	0.04069	0.03740	194	1.09	0.2779
**gamma_3c(variable)**	FEV1	0.07398	0.03685	191	2.01	0.0461
**gamma_3c(variable)**	PEFR	-0.00011	0.1220	193	-0.00	0.9993
**gamma_3c(variable)**	FEF2575	0.1480	0.05860	192	2.53	0.0123
**gamma_4c(variable)**	FVC	-0.01458	0.03740	194	-0.39	0.6970
**gamma_4c(variable)**	FEV1	-0.09538	0.03685	191	-2.59	0.0104
**gamma_4c(variable)**	PEFR	-0.2911	0.1220	193	-2.39	0.0180
**gamma_4c(variable)**	FEF2575	-0.2403	0.05860	192	-4.10	<.0001
**gamma_5c(variable)**	FVC	-0.04153	0.03732	204	-1.11	0.2671
**gamma_5c(variable)**	FEV1	0.001079	0.03653	198	0.03	0.9765
**gamma_5c(variable)**	PEFR	-0.1342	0.1203	202	-1.12	0.2658
**gamma_5c(variable)**	FEF2575	0.05339	0.05806	199	0.92	0.3590

Type 3 Tests of Fixed Effects

Effect	Num DF	Den DF	F Value	Pr > F
mu_a(variable)	4	299	23.63	<.0001
mu_b(variable)	4	328	4.89	0.0008
mu_c(variable)	4	232	8.17	<.0001
lambda(variable)	4	380	4.99	0.0006
gamma_1a(variable)	4	203	0.45	0.7736
gamma_2a(variable)	4	214	0.60	0.6596
gamma_3a(variable)	4	192	1.75	0.1414
gamma_4a(variable)	4	192	2.52	0.0429
gamma_5a(variable)	4	192	0.79	0.5342
gamma_1b(variable)	4	194	5.85	0.0002
gamma_2b(variable)	4	195	2.28	0.0626
gamma_3b(variable)	4	222	3.47	0.0090
gamma_4b(variable)	4	222	2.85	0.0247
gamma_5b(variable)	4	194	4.20	0.0028
gamma_2c(variable)	4	192	0.70	0.5932
gamma_3c(variable)	4	194	2.12	0.0794
gamma_4c(variable)	4	194	5.00	0.0007
gamma_5c(variable)	4	204	1.48	0.2103

Multivariate Analysis of FVC, FEV1, PEFR, and FEF2575 with Center as covariate

The Mixed Procedure

Model Information	
Data Set	WORK.TALC_3PERIOD
Dependent Variable	outcome
Covariance Structure	Unstructured
Subject Effect	participant
Estimation Method	ML
Residual Variance Method	None
Fixed Effects SE Method	Model-Based
Degrees of Freedom Method	Satterthwaite

Class Level Information

Class	Levels	Values
participant	187	1 2 3 5 7 8 10 11 12 13 14 15 16 17 18 19 20 21 22 23 24 25 26 27 28 29 30 31 32 33 35 36 37 38 39 41 42 43 44 45 47 48 50 52 53 54 55 56 57 58 59 60 61 62 63 65 66 67 68 69 70 71 72 73 74 75 76 77 78 79 80 81 82 83 84 85 86 87 88 91 92 93 94 95 96 97 98 99 100 101 102 103 104 105 106 108 109 110 111 113 114 116 117 118 119 120 121 122 123 124 125 126 127 128 129 130 131 132 133 134 135 136 137 139 140 141 142 143 144 145 146 147 148 149 150 151 152 153 154 155 156 157 158 159 160 161 163 164 165 167 168 170 171 173 174 175 176 179 180 181 182 183 184 185 186 187 188 189 190 191 192 193 194 196 197 198 199 200 201 202 203 204 206 207 208 209 210
variable	4	FVC FEV1 PEFR FEF2575
sequence	6	ABC ACB BAC BCA CAB CBA
period	3	1 2 3
treatment	3	Tiotropium Double-glucocorticoid Salmeterol

Dimensions	
Covariance Parameters	78
Columns in X	108
Columns in Z	0
Subjects	187
Max Obs per Subject	12

Number of Observations	
Number of Observations Read	2244
Number of Observations Used	2244

**Number of Observations Not Used**	0

Iteration History			
**Iteration**	**Evaluations**	**-2 Log Like**	**Criterion**
**0**	1	2501.22076931	
**1**	2	-914.87634552	0.00001313
**2**	1	-914.91013042	0.00000002
**3**	1	-914.91016880	0.00000000

Convergence criteria met.

Covariance Parameter Estimates		
**Cov Parm**	**Subject**	**Estimate**
**UN(1,1)**	participant	0.05754
**UN(2,1)**	participant	0.02164
**UN(2,2)**	participant	0.05404
**UN(3,1)**	participant	0.02632
**UN(3,2)**	participant	0.01651
**UN(3,3)**	participant	0.04751
**UN(4,1)**	participant	0.03855
**UN(4,2)**	participant	0.01437
**UN(4,3)**	participant	0.02245
**UN(4,4)**	participant	0.04486
**UN(5,1)**	participant	0.01679
**UN(5,2)**	participant	0.03002
**UN(5,3)**	participant	0.01530
**UN(5,4)**	participant	0.01773
**UN(5,5)**	participant	0.03079
**UN(6,1)**	participant	0.02016
**UN(6,2)**	participant	0.01417
**UN(6,3)**	participant	0.03634
**UN(6,4)**	participant	0.02518
**UN(6,5)**	participant	0.01917
**UN(6,6)**	participant	0.04612
**UN(7,1)**	participant	0.1151
**UN(7,2)**	participant	0.03208

**UN(7,3)**	participant	0.04038
**UN(7,4)**	participant	0.1018
**UN(7,5)**	participant	0.03014
**UN(7,6)**	participant	0.05050
**UN(7,7)**	participant	0.5947
**UN(8,1)**	participant	0.04248
**UN(8,2)**	participant	0.08435
**UN(8,3)**	participant	0.02193
**UN(8,4)**	participant	0.02516
**UN(8,5)**	participant	0.06089
**UN(8,6)**	participant	0.02681
**UN(8,7)**	participant	0.2276
**UN(8,8)**	participant	0.4321
**UN(9,1)**	participant	0.03067
**UN(9,2)**	participant	0.02796
**UN(9,3)**	participant	0.06132
**UN(9,4)**	participant	0.01748
**UN(9,5)**	participant	0.03678
**UN(9,6)**	participant	0.09260
**UN(9,7)**	participant	0.2028
**UN(9,8)**	participant	0.2074
**UN(9,9)**	participant	0.5383
**UN(10,1)**	participant	0.01571
**UN(10,2)**	participant	0.01369
**UN(10,3)**	participant	0.01553
**UN(10,4)**	participant	0.04928
**UN(10,5)**	participant	0.01885
**UN(10,6)**	participant	0.02731
**UN(10,7)**	participant	0.07708
**UN(10,8)**	participant	0.007609
**UN(10,9)**	participant	-0.00585
**UN(10,10)**	participant	0.1150
**UN(11,1)**	participant	0.006583
**UN(11,2)**	participant	0.000535
**UN(11,3)**	participant	0.01190

	participant	0.02056
**UN(11,5)**	participant	0.02839
**UN(11,6)**	participant	0.02164
**UN(11,7)**	participant	0.01254
**UN(11,8)**	participant	0.004758
**UN(11,9)**	participant	0.03463
**UN(11,10)**	participant	0.03253
**UN(11,11)**	participant	0.06967
**UN(12,1)**	participant	0.01459
**UN(12,2)**	participant	0.01529
**UN(12,3)**	participant	0.02966
**UN(12,4)**	participant	0.03355
**UN(12,5)**	participant	0.02590
**UN(12,6)**	participant	0.06119
**UN(12,7)**	participant	0.06197
**UN(12,8)**	participant	0.02164
**UN(12,9)**	participant	0.1212
**UN(12,10)**	participant	0.06400
**UN(12,11)**	participant	0.03876
**UN(12,12)**	participant	0.1217

**Fit Statistics**	
**-2 Log Likelihood**	-914.9
**AIC (Smaller is Better)**	-542.9
**AICC (Smaller is Better)**	-509.1
**BIC (Smaller is Better)**	58.1

Null Model Likelihood Ratio Test

DF	Chi-Square	Pr > ChiSq
77	3416.13	<.0001

Solution for Fixed Effects

Effect	variable	Estimate	Standard Error	DF	t Value	Pr > |t|
**mu_a(variable)**	FVC	0.2099	0.09986	197	2.10	0.0368
**mu_a(variable)**	FEV1	0.3287	0.09011	196	3.65	0.0003

**mu_a(variable)**	PEFR	0.9678	0.3062	199	3.16	0.0018
**mu_a(variable)**	FEF2575	0.5344	0.1332	199	4.01	<.0001
**mu_b(variable)**	FVC	0.1407	0.09981	197	1.41	0.1603
**mu_b(variable)**	FEV1	0.2290	0.08972	193	2.55	0.0115
**mu_b(variable)**	PEFR	0.6006	0.3050	196	1.97	0.0503
**mu_b(variable)**	FEF2575	0.4015	0.1323	194	3.03	0.0027
**mu_c(variable)**	FVC	0.1630	0.1004	196	1.62	0.1060
**mu_c(variable)**	FEV1	0.2656	0.09096	199	2.92	0.0039
**mu_c(variable)**	PEFR	0.7744	0.3087	200	2.51	0.0129
**mu_c(variable)**	FEF2575	0.4617	0.1347	203	3.43	0.0007
**lambda(variable)**	FVC	-0.06059	0.01656	399	-3.66	0.0003
**lambda(variable)**	FEV1	-0.03608	0.01388	364	-2.60	0.0097
**lambda(variable)**	PEFR	0.009259	0.05412	343	0.17	0.8643
**lambda(variable)**	FEF2575	-0.01976	0.02312	367	-0.85	0.3934
**gamma_1a(variable)**	FVC	-0.03173	0.03813	198	-0.83	0.4062
**gamma_1a(variable)**	FEV1	-0.03275	0.03419	197	-0.96	0.3393
**gamma_1a(variable)**	PEFR	0.03271	0.1202	197	0.27	0.7858
**gamma_1a(variable)**	FEF2575	-0.02919	0.05449	199	-0.54	0.5927
**gamma_2a(variable)**	FVC	0.01103	0.03711	209	0.30	0.7665
**gamma_2a(variable)**	FEV1	-0.00244	0.03284	210	-0.07	0.9407
**gamma_2a(variable)**	PEFR	0.1416	0.1189	210	1.19	0.2347
**gamma_2a(variable)**	FEF2575	-0.02758	0.05231	213	-0.53	0.5985
**gamma_3a(variable)**	FVC	0.06277	0.03971	185	1.58	0.1157
**gamma_3a(variable)**	FEV1	0.07979	0.03500	190	2.28	0.0237
**gamma_3a(variable)**	PEFR	-0.00639	0.1281	186	-0.05	0.9603
**gamma_3a(variable)**	FEF2575	0.1089	0.05566	190	1.96	0.0519
**gamma_4a(variable)**	FVC	0.01726	0.03972	185	0.43	0.6644
**gamma_4a(variable)**	FEV1	-0.03680	0.03500	190	-1.05	0.2944
**gamma_4a(variable)**	PEFR	-0.2563	0.1281	186	-2.00	0.0468
**gamma_4a(variable)**	FEF2575	-0.1201	0.05567	190	-2.16	0.0322
**gamma_5a(variable)**	FVC	-0.04972	0.04001	184	-1.24	0.2156
**gamma_5a(variable)**	FEV1	-0.02236	0.03554	188	-0.63	0.5300
**gamma_5a(variable)**	PEFR	-0.1886	0.1275	185	-1.48	0.1409
**gamma_5a(variable)**	FEF2575	0.01716	0.05710	189	0.30	0.7641
**gamma_1b(variable)**	FVC	-0.06983	0.03513	185	-1.99	0.0483

	FEV1	-0.02746	0.02608	190	-1.05	0.2936
**gamma_1b(variable)**	PEFR	-0.1825	0.09887	191	-1.85	0.0664
**gamma_1b(variable)**	FEF2575	-0.04612	0.03933	189	-1.17	0.2424
**gamma_2b(variable)**	FVC	-0.01437	0.03762	186	-0.38	0.7029
**gamma_2b(variable)**	FEV1	-0.01412	0.02862	192	-0.49	0.6224
**gamma_2b(variable)**	PEFR	0.1080	0.1073	193	1.01	0.3155
**gamma_2b(variable)**	FEF2575	0.02714	0.04293	191	0.63	0.5280
**gamma_3b(variable)**	FVC	0.04570	0.03689	206	1.24	0.2168
**gamma_3b(variable)**	FEV1	0.07590	0.02888	218	2.63	0.0092
**gamma_3b(variable)**	PEFR	-0.1082	0.1076	223	-1.01	0.3155
**gamma_3b(variable)**	FEF2575	0.09957	0.04354	222	2.29	0.0231
**gamma_4b(variable)**	FVC	0.03666	0.03692	207	0.99	0.3219
**gamma_4b(variable)**	FEV1	-0.04294	0.02892	218	-1.48	0.1391
**gamma_4b(variable)**	PEFR	-0.04884	0.1077	223	-0.45	0.6507
**gamma_4b(variable)**	FEF2575	-0.1376	0.04359	222	-3.16	0.0018
**gamma_5b(variable)**	FVC	-0.1120	0.03827	185	-2.93	0.0039
**gamma_5b(variable)**	FEV1	-0.04070	0.02951	192	-1.38	0.1694
**gamma_5b(variable)**	PEFR	-0.3666	0.1096	192	-3.34	0.0010
**gamma_5b(variable)**	FEF2575	0.07802	0.04443	189	1.76	0.0807
**gamma_2c(variable)**	FVC	0.03503	0.03498	186	1.00	0.3180
**gamma_2c(variable)**	FEV1	0.01538	0.03415	182	0.45	0.6531
**gamma_2c(variable)**	PEFR	0.1547	0.1154	188	1.34	0.1819
**gamma_2c(variable)**	FEF2575	-0.00163	0.05564	185	-0.03	0.9766
**gamma_3c(variable)**	FVC	0.04551	0.03501	189	1.30	0.1952
**gamma_3c(variable)**	FEV1	0.07846	0.03448	184	2.28	0.0240
**gamma_3c(variable)**	PEFR	-0.00373	0.1185	190	-0.03	0.9749
**gamma_3c(variable)**	FEF2575	0.1528	0.05565	186	2.75	0.0066
**gamma_4c(variable)**	FVC	-0.02443	0.03505	189	-0.70	0.4867
**gamma_4c(variable)**	FEV1	-0.1038	0.03452	185	-3.01	0.0030
**gamma_4c(variable)**	PEFR	-0.3069	0.1186	190	-2.59	0.0104
**gamma_4c(variable)**	FEF2575	-0.2472	0.05570	187	-4.44	<.0001
**gamma_5c(variable)**	FVC	-0.04645	0.03491	201	-1.33	0.1849
**gamma_5c(variable)**	FEV1	-0.00154	0.03415	191	-0.05	0.9640
**gamma_5c(variable)**	PEFR	-0.1223	0.1168	200	-1.05	0.2963
**gamma_5c(variable)**	FEF2575	0.05250	0.05508	194	0.95	0.3417

	FVC	-0.1269	0.1053	187	-1.21	0.2297
**center1(variable)**	FEV1	-0.1752	0.09513	187	-1.84	0.0671
**center1(variable)**	PEFR	-0.3656	0.3221	187	-1.14	0.2577
**center1(variable)**	FEF2575	-0.2698	0.1400	187	-1.93	0.0555
**center2(variable)**	FVC	-0.1147	0.1035	187	-1.11	0.2691
**center2(variable)**	FEV1	-0.1437	0.09353	187	-1.54	0.1261
**center2(variable)**	PEFR	-0.3587	0.3166	187	-1.13	0.2587
**center2(variable)**	FEF2575	-0.2758	0.1376	187	-2.00	0.0465
**center3(variable)**	FVC	-0.1092	0.1050	187	-1.04	0.2993
**center3(variable)**	FEV1	-0.1842	0.09484	187	-1.94	0.0537
**center3(variable)**	PEFR	-0.5509	0.3211	187	-1.72	0.0878
**center3(variable)**	FEF2575	-0.3431	0.1396	187	-2.46	0.0149
**center5(variable)**	FVC	-0.1053	0.1060	187	-0.99	0.3217
**center5(variable)**	FEV1	-0.2282	0.09574	187	-2.38	0.0181
**center5(variable)**	PEFR	-0.4687	0.3241	187	-1.45	0.1498
**center5(variable)**	FEF2575	-0.4741	0.1409	187	-3.36	0.0009
**center6(variable)**	FVC	0.01965	0.1038	187	0.19	0.8501
**center6(variable)**	FEV1	-0.07733	0.09381	187	-0.82	0.4108
**center6(variable)**	PEFR	-0.4229	0.3176	187	-1.33	0.1846
**center6(variable)**	FEF2575	-0.2587	0.1381	187	-1.87	0.0625
**center7(variable)**	FVC	-0.07649	0.1120	187	-0.68	0.4954
**center7(variable)**	FEV1	-0.2006	0.1012	187	-1.98	0.0489
**center7(variable)**	PEFR	-0.3102	0.3425	187	-0.91	0.3663
**center7(variable)**	FEF2575	-0.4110	0.1489	187	-2.76	0.0064
**center8(variable)**	FVC	-0.08905	0.1024	187	-0.87	0.3855
**center8(variable)**	FEV1	-0.2389	0.09251	187	-2.58	0.0106
**center8(variable)**	PEFR	-0.5190	0.3132	187	-1.66	0.0992
**center8(variable)**	FEF2575	-0.4801	0.1361	187	-3.53	0.0005
**center9(variable)**	FVC	0.05941	0.1073	187	0.55	0.5805
**center9(variable)**	FEV1	-0.1234	0.09699	187	-1.27	0.2049
**center9(variable)**	PEFR	-0.4757	0.3284	187	-1.45	0.1491
**center9(variable)**	FEF2575	-0.4125	0.1427	187	-2.89	0.0043
**center10(variable)**	FVC	0.1527	0.1120	187	1.36	0.1742
**center10(variable)**	FEV1	0.08790	0.1012	187	0.87	0.3861
**center10(variable)**	PEFR	0.4330	0.3426	187	1.26	0.2078

	FEF2575	-0.04328	0.1489	187	-0.29	0.7716

Type 3 Tests of Fixed Effects				
**Effect**	**Num DF**	**Den DF**	**F Value**	**Pr > F**
**mu_a(variable)**	4	200	5.34	0.0004
**mu_b(variable)**	4	196	2.84	0.0255
**mu_c(variable)**	4	200	3.80	0.0053
**lambda(variable)**	4	375	5.19	0.0004
**gamma_1a(variable)**	4	198	0.56	0.6931
**gamma_2a(variable)**	4	210	0.64	0.6368
**gamma_3a(variable)**	4	187	2.23	0.0676
**gamma_4a(variable)**	4	187	2.85	0.0251
**gamma_5a(variable)**	4	186	0.76	0.5515
**gamma_1b(variable)**	4	189	6.49	<.0001
**gamma_2b(variable)**	4	190	2.71	0.0316
**gamma_3b(variable)**	4	220	4.12	0.0030
**gamma_4b(variable)**	4	221	3.16	0.0151
**gamma_5b(variable)**	4	190	4.21	0.0027
**gamma_2c(variable)**	4	188	0.86	0.4863
**gamma_3c(variable)**	4	190	2.57	0.0397
**gamma_4c(variable)**	4	191	5.53	0.0003
**gamma_5c(variable)**	4	202	1.61	0.1726
**center1(variable)**	4	187	1.11	0.3531
**center2(variable)**	4	187	1.63	0.1678
**center3(variable)**	4	187	2.04	0.0908
**center5(variable)**	4	187	3.19	0.0145
**center6(variable)**	4	187	1.73	0.1460
**center7(variable)**	4	187	1.95	0.1038
**center8(variable)**	4	187	3.36	0.0111
**center9(variable)**	4	187	3.29	0.0125
**center10(variable)**	4	187	0.82	0.5138

LR test statistic and p-value for H_0: beta_center=0

Obs	Descr	reduced_model_likeli	full_model_likeli	lr_statistic	df	p_value
1	-2 Log Likelihood	-811.8	-914.9	103.118	36	2.1598E-8

Multivariate Analysis with Center as covariate - reduced model mu_A=mu_B

The Mixed Procedure

Model Information	
Data Set	WORK.TALC_3PERIOD_TEST_MEAN
Dependent Variable	outcome
Covariance Structure	Unstructured
Subject Effect	participant
Estimation Method	ML
Residual Variance Method	None
Fixed Effects SE Method	Model-Based
Degrees of Freedom Method	Satterthwaite

Class	Levels	Values
participant	187	1 2 3 5 7 8 10 11 12 13 14 15 16 17 18 19 20 21 22 23 24 25 26 27 28 29 30 31 32 33 35 36 37 38 39 41 42 43 44 45 47 48 50 52 53 54 55 56 57 58 59 60 61 62 63 65 66 67 68 69 70 71 72 73 74 75 76 77 78 79 80 81 82 83 84 85 86 87 88 91 92 93 94 95 96 97 98 99 100 101 102 103 104 105 106 108 109 110 111 113 114 116 117 118 119 120 121 122 123 124 125 126 127 128 129 130 131 132 133 134 135 136 137 139 140 141 142 143 144 145 146 147 148 149 150 151 152 153 154 155 156 157 158 159 160 161 163 164 165 167 168 170 171 173 174 175 176 179 180 181 182 183 184 185 186 187 188 189 190 191 192 193 194 196 197 198 199 200 201 202 203 204 206 207 208 209 210
variable	4	FVC FEV1 PEFR FEF2575
sequence	6	ABC ACB BAC BCA CAB CBA
period	3	1 2 3
treatment	3	Tiotropium Double-glucocorticoid Salmeterol

Dimensions	
Covariance Parameters	78
Columns in X	104
Columns in Z	0
Subjects	187
Max Obs per Subject	12

Number of Observations	
Number of Observations Read	2244
Number of Observations Used	2244

Number of Observations Not Used	0

Iteration History

Iteration	Evaluations	-2 Log Like	Criterion
0	1	2586.70826061	
1	2	-860.30722542	0.00083295
2	1	-862.67787467	0.00005747
3	1	-862.82844037	0.00000038
4	1	-862.82938505	0.00000000

Convergence criteria met.

Covariance Parameter Estimates

Cov Parm	Subject	Estimate
UN(1,1)	participant	0.05975
UN(2,1)	participant	0.02061
UN(2,2)	participant	0.05453
UN(3,1)	participant	0.02700
UN(3,2)	participant	0.01620
UN(3,3)	participant	0.04771
UN(4,1)	participant	0.04188
UN(4,2)	participant	0.01281
UN(4,3)	participant	0.02347
UN(4,4)	participant	0.04988
UN(5,1)	participant	0.01545
UN(5,2)	participant	0.03065
UN(5,3)	participant	0.01489
UN(5,4)	participant	0.01570
UN(5,5)	participant	0.03161
UN(6,1)	participant	0.02101
UN(6,2)	participant	0.01377
UN(6,3)	participant	0.03660
UN(6,4)	participant	0.02646
UN(6,5)	participant	0.01865
UN(6,6)	participant	0.04644
UN(7,1)	participant	0.1267

**UN(7,2)**	participant	0.02663
**UN(7,3)**	participant	0.04393
**UN(7,4)**	participant	0.1193
**UN(7,5)**	participant	0.02305
**UN(7,6)**	participant	0.05500
**UN(7,7)**	participant	0.6561
**UN(8,1)**	participant	0.03691
**UN(8,2)**	participant	0.08696
**UN(8,3)**	participant	0.02023
**UN(8,4)**	participant	0.01677
**UN(8,5)**	participant	0.06428
**UN(8,6)**	participant	0.02466
**UN(8,7)**	participant	0.1983
**UN(8,8)**	participant	0.4462
**UN(9,1)**	participant	0.02847
**UN(9,2)**	participant	0.02899
**UN(9,3)**	participant	0.06065
**UN(9,4)**	participant	0.01417
**UN(9,5)**	participant	0.03811
**UN(9,6)**	participant	0.09175
**UN(9,7)**	participant	0.1912
**UN(9,8)**	participant	0.2129
**UN(9,9)**	participant	0.5405
**UN(10,1)**	participant	0.02072
**UN(10,2)**	participant	0.01134
**UN(10,3)**	participant	0.01705
**UN(10,4)**	participant	0.05683
**UN(10,5)**	participant	0.01580
**UN(10,6)**	participant	0.02925
**UN(10,7)**	participant	0.1035
**UN(10,8)**	participant	-0.00503
**UN(10,9)**	participant	-0.01083
**UN(10,10)**	participant	0.1264
**UN(11,1)**	participant	0.005366
**UN(11,2)**	participant	0.001105

	participant	0.01152
**UN(11,4)**	participant	0.01873
**UN(11,5)**	participant	0.02913
**UN(11,6)**	participant	0.02117
**UN(11,7)**	participant	0.006126
**UN(11,8)**	participant	0.007825
**UN(11,9)**	participant	0.03584
**UN(11,10)**	participant	0.02977
**UN(11,11)**	participant	0.07034
**UN(12,1)**	participant	0.01647
**UN(12,2)**	participant	0.01441
**UN(12,3)**	participant	0.03024
**UN(12,4)**	participant	0.03637
**UN(12,5)**	participant	0.02476
**UN(12,6)**	participant	0.06191
**UN(12,7)**	participant	0.07186
**UN(12,8)**	participant	0.01691
**UN(12,9)**	participant	0.1193
**UN(12,10)**	participant	0.06825
**UN(12,11)**	participant	0.03773
**UN(12,12)**	participant	0.1232

Fit Statistics

**-2 Log Likelihood**	-862.8
**AIC (Smaller is Better)**	-498.8
**AICC (Smaller is Better)**	-466.5
**BIC (Smaller is Better)**	89.2

Null Model Likelihood Ratio Test

DF	Chi-Square	Pr > ChiSq
77	3449.54	<.0001

Solution for Fixed Effects

Effect	variable	Estimate	Standard Error	DF	t Value	Pr > |t|
**mu_ab(variable)**	FVC	0.1638	0.09935	193	1.65	0.1009

mu_ab(variable)	FEV1	0.2593	0.08961	192	2.89	0.0043
mu_ab(variable)	PEFR	0.7289	0.3043	194	2.39	0.0176
mu_ab(variable)	FEF2575	0.4292	0.1321	193	3.25	0.0014
mu_c(variable)	FVC	0.1491	0.1003	196	1.49	0.1388
mu_c(variable)	FEV1	0.2483	0.09095	199	2.73	0.0069
mu_c(variable)	PEFR	0.8310	0.3086	200	2.69	0.0077
mu_c(variable)	FEF2575	0.4222	0.1345	202	3.14	0.0020
lambda(variable)	FVC	-0.06210	0.01677	400	-3.70	0.0002
lambda(variable)	FEV1	-0.03844	0.01440	374	-2.67	0.0079
lambda(variable)	PEFR	-0.00484	0.05664	324	-0.09	0.9320
lambda(variable)	FEF2575	-0.02242	0.02365	376	-0.95	0.3438
gamma_1a(variable)	FVC	-0.02619	0.03870	188	-0.68	0.4993
gamma_1a(variable)	FEV1	-0.02447	0.03562	174	-0.69	0.4930
gamma_1a(variable)	PEFR	0.05776	0.1243	178	0.46	0.6428
gamma_1a(variable)	FEF2575	-0.01613	0.05664	179	-0.28	0.7761
gamma_2a(variable)	FVC	0.01150	0.03770	197	0.30	0.7607
gamma_2a(variable)	FEV1	-0.00181	0.03433	184	-0.05	0.9579
gamma_2a(variable)	PEFR	0.1400	0.1238	187	1.13	0.2596
gamma_2a(variable)	FEF2575	-0.02603	0.05448	190	-0.48	0.6333
gamma_3a(variable)	FVC	0.06169	0.04039	175	1.53	0.1284
gamma_3a(variable)	FEV1	0.07820	0.03672	166	2.13	0.0347
gamma_3a(variable)	PEFR	-0.01002	0.1339	165	-0.07	0.9405
gamma_3a(variable)	FEF2575	0.1062	0.05815	171	1.83	0.0696
gamma_4a(variable)	FVC	0.01618	0.04039	175	0.40	0.6892
gamma_4a(variable)	FEV1	-0.03839	0.03672	166	-1.05	0.2973
gamma_4a(variable)	PEFR	-0.2599	0.1340	165	-1.94	0.0540
gamma_4a(variable)	FEF2575	-0.1228	0.05816	171	-2.11	0.0362
gamma_5a(variable)	FVC	-0.05080	0.04079	173	-1.25	0.2146
gamma_5a(variable)	FEV1	-0.02395	0.03747	165	-0.64	0.5235
gamma_5a(variable)	PEFR	-0.1923	0.1338	164	-1.44	0.1526
gamma_5a(variable)	FEF2575	0.01446	0.05984	171	0.24	0.8094
gamma_1b(variable)	FVC	-0.07239	0.03525	183	-2.05	0.0414
gamma_1b(variable)	FEV1	-0.03065	0.02638	181	-1.16	0.2468
gamma_1b(variable)	PEFR	-0.1943	0.1000	183	-1.94	0.0536
gamma_1b(variable)	FEF2575	-0.04883	0.03950	186	-1.24	0.2179

	FVC	-0.01456	0.03775	184	-0.39	0.7002
**gamma_2b(variable)**	FEV1	-0.01422	0.02889	184	-0.49	0.6231
**gamma_2b(variable)**	PEFR	0.1090	0.1085	184	1.00	0.3167
**gamma_2b(variable)**	FEF2575	0.02722	0.04306	189	0.63	0.5280
**gamma_3b(variable)**	FVC	0.04544	0.03701	204	1.23	0.2210
**gamma_3b(variable)**	FEV1	0.07528	0.02916	212	2.58	0.0105
**gamma_3b(variable)**	PEFR	-0.1137	0.1091	214	-1.04	0.2989
**gamma_3b(variable)**	FEF2575	0.09867	0.04370	222	2.26	0.0249
**gamma_4b(variable)**	FVC	0.03640	0.03705	205	0.98	0.3270
**gamma_4b(variable)**	FEV1	-0.04355	0.02920	212	-1.49	0.1373
**gamma_4b(variable)**	PEFR	-0.05425	0.1093	214	-0.50	0.6200
**gamma_4b(variable)**	FEF2575	-0.1385	0.04375	222	-3.16	0.0018
**gamma_5b(variable)**	FVC	-0.1108	0.03844	183	-2.88	0.0044
**gamma_5b(variable)**	FEV1	-0.03895	0.02991	182	-1.30	0.1945
**gamma_5b(variable)**	PEFR	-0.3579	0.1117	179	-3.20	0.0016
**gamma_5b(variable)**	FEF2575	0.07977	0.04465	186	1.79	0.0757
**gamma_2c(variable)**	FVC	0.03648	0.03512	183	1.04	0.3004
**gamma_2c(variable)**	FEV1	0.01739	0.03440	177	0.51	0.6138
**gamma_2c(variable)**	PEFR	0.1576	0.1155	188	1.36	0.1743
**gamma_2c(variable)**	FEF2575	0.001785	0.05620	177	0.03	0.9747
**gamma_3c(variable)**	FVC	0.04603	0.03507	188	1.31	0.1910
**gamma_3c(variable)**	FEV1	0.07930	0.03458	183	2.29	0.0230
**gamma_3c(variable)**	PEFR	0.002184	0.1190	188	0.02	0.9854
**gamma_3c(variable)**	FEF2575	0.1537	0.05595	182	2.75	0.0066
**gamma_4c(variable)**	FVC	-0.02391	0.03511	188	-0.68	0.4967
**gamma_4c(variable)**	FEV1	-0.1029	0.03461	183	-2.97	0.0033
**gamma_4c(variable)**	PEFR	-0.3010	0.1191	188	-2.53	0.0123
**gamma_4c(variable)**	FEF2575	-0.2464	0.05599	183	-4.40	<.0001
**gamma_5c(variable)**	FVC	-0.04744	0.03501	200	-1.35	0.1770
**gamma_5c(variable)**	FEV1	-0.00307	0.03434	190	-0.09	0.9289
**gamma_5c(variable)**	PEFR	-0.1305	0.1174	200	-1.11	0.2678
**gamma_5c(variable)**	FEF2575	0.05067	0.05550	190	0.91	0.3624
**center1(variable)**	FVC	-0.1269	0.1053	187	-1.21	0.2297
**center1(variable)**	FEV1	-0.1752	0.09513	187	-1.84	0.0671
**center1(variable)**	PEFR	-0.3656	0.3221	187	-1.14	0.2577

	FEF2575	-0.2698	0.1400	187	-1.93	0.0555
**center2(variable)**	FVC	-0.1147	0.1035	187	-1.11	0.2691
**center2(variable)**	FEV1	-0.1437	0.09353	187	-1.54	0.1261
**center2(variable)**	PEFR	-0.3587	0.3166	187	-1.13	0.2587
**center2(variable)**	FEF2575	-0.2758	0.1376	187	-2.00	0.0465
**center3(variable)**	FVC	-0.1092	0.1050	187	-1.04	0.2993
**center3(variable)**	FEV1	-0.1842	0.09484	187	-1.94	0.0537
**center3(variable)**	PEFR	-0.5509	0.3211	187	-1.72	0.0878
**center3(variable)**	FEF2575	-0.3431	0.1396	187	-2.46	0.0149
**center5(variable)**	FVC	-0.1053	0.1060	187	-0.99	0.3217
**center5(variable)**	FEV1	-0.2282	0.09574	187	-2.38	0.0181
**center5(variable)**	PEFR	-0.4687	0.3241	187	-1.45	0.1498
**center5(variable)**	FEF2575	-0.4741	0.1409	187	-3.36	0.0009
**center6(variable)**	FVC	0.01965	0.1038	187	0.19	0.8501
**center6(variable)**	FEV1	-0.07733	0.09381	187	-0.82	0.4108
**center6(variable)**	PEFR	-0.4229	0.3176	187	-1.33	0.1846
**center6(variable)**	FEF2575	-0.2587	0.1381	187	-1.87	0.0625
**center7(variable)**	FVC	-0.07649	0.1120	187	-0.68	0.4954
**center7(variable)**	FEV1	-0.2006	0.1012	187	-1.98	0.0489
**center7(variable)**	PEFR	-0.3102	0.3425	187	-0.91	0.3663
**center7(variable)**	FEF2575	-0.4110	0.1489	187	-2.76	0.0064
**center8(variable)**	FVC	-0.08905	0.1024	187	-0.87	0.3855
**center8(variable)**	FEV1	-0.2389	0.09251	187	-2.58	0.0106
**center8(variable)**	PEFR	-0.5190	0.3132	187	-1.66	0.0992
**center8(variable)**	FEF2575	-0.4801	0.1361	187	-3.53	0.0005
**center9(variable)**	FVC	0.05942	0.1073	187	0.55	0.5805
**center9(variable)**	FEV1	-0.1234	0.09699	187	-1.27	0.2049
**center9(variable)**	PEFR	-0.4757	0.3284	187	-1.45	0.1491
**center9(variable)**	FEF2575	-0.4125	0.1427	187	-2.89	0.0043
**center10(variable)**	FVC	0.1527	0.1120	187	1.36	0.1742
**center10(variable)**	FEV1	0.08790	0.1012	187	0.87	0.3861
**center10(variable)**	PEFR	0.4330	0.3426	187	1.26	0.2078
**center10(variable)**	FEF2575	-0.04328	0.1489	187	-0.29	0.7716

Type 3 Tests of Fixed Effects

Effect	Num DF	Den DF	F Value	Pr > F
mu_ab(variable)	4	193	3.39	0.0105
mu_c(variable)	4	200	3.51	0.0085
lambda(variable)	4	368	5.03	0.0006
gamma_1a(variable)	4	190	0.53	0.7124
gamma_2a(variable)	4	200	0.60	0.6616
gamma_3a(variable)	4	179	2.13	0.0788
gamma_4a(variable)	4	179	2.68	0.0333
gamma_5a(variable)	4	178	0.75	0.5587
gamma_1b(variable)	4	186	6.52	<.0001
gamma_2b(variable)	4	187	2.73	0.0303
gamma_3b(variable)	4	218	4.16	0.0029
gamma_4b(variable)	4	218	3.18	0.0145
gamma_5b(variable)	4	186	4.07	0.0035
gamma_2c(variable)	4	185	0.87	0.4849
gamma_3c(variable)	4	187	2.49	0.0449
gamma_4c(variable)	4	187	5.46	0.0004
gamma_5c(variable)	4	198	1.63	0.1676
center1(variable)	4	187	1.11	0.3531
center2(variable)	4	187	1.63	0.1678
center3(variable)	4	187	2.04	0.0908
center5(variable)	4	187	3.19	0.0145
center6(variable)	4	187	1.73	0.1460
center7(variable)	4	187	1.95	0.1038
center8(variable)	4	187	3.36	0.0111
center9(variable)	4	187	3.29	0.0125
center10(variable)	4	187	0.82	0.5138

LR test statistic and p-value for H_0: mu_A=mu_B

Obs	Descr	reduced_model_likeli	full_model_likeli	lr_statistic	df	p_value
1	-2 Log Likelihood	-862.8	-914.9	52.0808	4	1.3268E-10

Multivariate Analysis with Center as covariate - reduced model mu_A=mu_C

The Mixed Procedure

Model Information	
Data Set	WORK.TALC_3PERIOD_TEST_MEAN
Dependent Variable	outcome
Covariance Structure	Unstructured
Subject Effect	participant
Estimation Method	ML
Residual Variance Method	None
Fixed Effects SE Method	Model-Based
Degrees of Freedom Method	Satterthwaite

Class Level Information		
Class	Levels	Values
participant	187	1 2 3 5 7 8 10 11 12 13 14 15 16 17 18 19 20 21 22 23 24 25 26 27 28 29 30 31 32 33 35 36 37 38 39 41 42 43 44 45 47 48 50 52 53 54 55 56 57 58 59 60 61 62 63 65 66 67 68 69 70 71 72 73 74 75 76 77 78 79 80 81 82 83 84 85 86 87 88 91 92 93 94 95 96 97 98 99 100 101 102 103 104 105 106 108 109 110 111 113 114 116 117 118 119 120 121 122 123 124 125 126 127 128 129 130 131 132 133 134 135 136 137 139 140 141 142 143 144 145 146 147 148 149 150 151 152 153 154 155 156 157 158 159 160 161 163 164 165 167 168 170 171 173 174 175 176 179 180 181 182 183 184 185 186 187 188 189 190 191 192 193 194 196 197 198 199 200 201 202 203 204 206 207 208 209 210
variable	4	FVC FEV1 PEFR FEF2575
sequence	6	ABC ACB BAC BCA CAB CBA
period	3	1 2 3
treatment	3	Tiotropium Double-glucocorticoid Salmeterol

Dimensions	
Covariance Parameters	78
Columns in X	104
Columns in Z	0
Subjects	187
Max Obs per Subject	12

Number of Observations	
Number of Observations Read	2244
Number of Observations Used	2244

Number of Observations Not Used	0

Iteration History

Iteration	Evaluations	-2 Log Like	Criterion
0	1	2524.20405270	
1	2	-899.61321140	0.00001617
2	1	-899.65478617	0.00000002
3	1	-899.65484402	0.00000000

Convergence criteria met.

Covariance Parameter Estimates

Cov Parm	Subject	Estimate
UN(1,1)	participant	0.05819
UN(2,1)	participant	0.02166
UN(2,2)	participant	0.05408
UN(3,1)	participant	0.02610
UN(3,2)	participant	0.01663
UN(3,3)	participant	0.04805
UN(4,1)	participant	0.03930
UN(4,2)	participant	0.01439
UN(4,3)	participant	0.02220
UN(4,4)	participant	0.04573
UN(5,1)	participant	0.01683
UN(5,2)	participant	0.03007
UN(5,3)	participant	0.01548
UN(5,4)	participant	0.01777
UN(5,5)	participant	0.03087
UN(6,1)	participant	0.01974
UN(6,2)	participant	0.01433
UN(6,3)	participant	0.03713
UN(6,4)	participant	0.02471
UN(6,5)	participant	0.01941
UN(6,6)	participant	0.04730
UN(7,1)	participant	0.1169
UN(7,2)	participant	0.03213

**UN(7,3)**	participant	0.03969
**UN(7,4)**	participant	0.1039
**UN(7,5)**	participant	0.03022
**UN(7,6)**	participant	0.04923
**UN(7,7)**	participant	0.6001
**UN(8,1)**	participant	0.04243
**UN(8,2)**	participant	0.08444
**UN(8,3)**	participant	0.02228
**UN(8,4)**	participant	0.02511
**UN(8,5)**	participant	0.06102
**UN(8,6)**	participant	0.02730
**UN(8,7)**	participant	0.2275
**UN(8,8)**	participant	0.4323
**UN(9,1)**	participant	0.02868
**UN(9,2)**	participant	0.02839
**UN(9,3)**	participant	0.06378
**UN(9,4)**	participant	0.01521
**UN(9,5)**	participant	0.03740
**UN(9,6)**	participant	0.09637
**UN(9,7)**	participant	0.1969
**UN(9,8)**	participant	0.2088
**UN(9,9)**	participant	0.5512
**UN(10,1)**	participant	0.01645
**UN(10,2)**	participant	0.01370
**UN(10,3)**	participant	0.01526
**UN(10,4)**	participant	0.05014
**UN(10,5)**	participant	0.01889
**UN(10,6)**	participant	0.02681
**UN(10,7)**	participant	0.07923
**UN(10,8)**	participant	0.007535
**UN(10,9)**	participant	-0.00818
**UN(10,10)**	participant	0.1159
**UN(11,1)**	participant	0.006693
**UN(11,2)**	participant	0.000593
**UN(11,3)**	participant	0.01205

	participant	0.02069
**UN(11,5)**	participant	0.02848
**UN(11,6)**	participant	0.02184
**UN(11,7)**	participant	0.01283
**UN(11,8)**	participant	0.004889
**UN(11,9)**	participant	0.03503
**UN(11,10)**	participant	0.03265
**UN(11,11)**	participant	0.06977
**UN(12,1)**	participant	0.01405
**UN(12,2)**	participant	0.01549
**UN(12,3)**	participant	0.03066
**UN(12,4)**	participant	0.03294
**UN(12,5)**	participant	0.02620
**UN(12,6)**	participant	0.06267
**UN(12,7)**	participant	0.06035
**UN(12,8)**	participant	0.02225
**UN(12,9)**	participant	0.1259
**UN(12,10)**	participant	0.06336
**UN(12,11)**	participant	0.03900
**UN(12,12)**	participant	0.1235

Fit Statistics	
**-2 Log Likelihood**	-899.7
**AIC (Smaller is Better)**	-535.7
**AICC (Smaller is Better)**	-503.3
**BIC (Smaller is Better)**	52.4

Null Model Likelihood Ratio Test

DF	Chi-Square	Pr > ChiSq
77	3423.86	<.0001

Solution for Fixed Effects

Effect	variable	Estimate	Standard Error	DF	t Value	Pr > |t|
**mu_ac(variable)**	FVC	0.1941	0.09969	192	1.95	0.0530
**mu_ac(variable)**	FEV1	0.3120	0.09017	192	3.46	0.0007

**mu_ac(variable)**	PEFR	0.9208	0.3053	194	3.02	0.0029
**mu_ac(variable)**	FEF2575	0.5203	0.1332	196	3.91	0.0001
**mu_b(variable)**	FVC	0.1499	0.09993	195	1.50	0.1352
**mu_b(variable)**	FEV1	0.2415	0.08989	191	2.69	0.0079
**mu_b(variable)**	PEFR	0.6298	0.3050	195	2.07	0.0402
**mu_b(variable)**	FEF2575	0.4142	0.1323	193	3.13	0.0020
**lambda(variable)**	FVC	-0.06807	0.01649	397	-4.13	<.0001
**lambda(variable)**	FEV1	-0.04680	0.01388	361	-3.37	0.0008
**lambda(variable)**	PEFR	-0.01407	0.05404	342	-0.26	0.7948
**lambda(variable)**	FEF2575	-0.03459	0.02293	371	-1.51	0.1322
**gamma_1a(variable)**	FVC	-0.05389	0.03736	213	-1.44	0.1507
**gamma_1a(variable)**	FEV1	-0.06109	0.03284	228	-1.86	0.0642
**gamma_1a(variable)**	PEFR	-0.02794	0.1192	203	-0.23	0.8150
**gamma_1a(variable)**	FEF2575	-0.05844	0.05232	230	-1.12	0.2652
**gamma_2a(variable)**	FVC	0.008843	0.03729	206	0.24	0.8128
**gamma_2a(variable)**	FEV1	-0.00627	0.03311	205	-0.19	0.8499
**gamma_2a(variable)**	PEFR	0.1328	0.1193	207	1.11	0.2669
**gamma_2a(variable)**	FEF2575	-0.03446	0.05245	210	-0.66	0.5119
**gamma_3a(variable)**	FVC	0.06916	0.03986	183	1.74	0.0844
**gamma_3a(variable)**	FEV1	0.08841	0.03523	185	2.51	0.0129
**gamma_3a(variable)**	PEFR	0.01252	0.1285	184	0.10	0.9225
**gamma_3a(variable)**	FEF2575	0.1185	0.05570	188	2.13	0.0347
**gamma_4a(variable)**	FVC	0.02352	0.03987	183	0.59	0.5559
**gamma_4a(variable)**	FEV1	-0.02834	0.03523	185	-0.80	0.4223
**gamma_4a(variable)**	PEFR	-0.2377	0.1286	184	-1.85	0.0661
**gamma_4a(variable)**	FEF2575	-0.1107	0.05571	188	-1.99	0.0484
**gamma_5a(variable)**	FVC	-0.04359	0.04016	181	-1.09	0.2792
**gamma_5a(variable)**	FEV1	-0.01449	0.03576	184	-0.41	0.6859
**gamma_5a(variable)**	PEFR	-0.1717	0.1279	183	-1.34	0.1811
**gamma_5a(variable)**	FEF2575	0.02607	0.05717	187	0.46	0.6489
**gamma_1b(variable)**	FVC	-0.08034	0.03497	187	-2.30	0.0227
**gamma_1b(variable)**	FEV1	-0.04263	0.02569	198	-1.66	0.0986
**gamma_1b(variable)**	PEFR	-0.2053	0.09831	194	-2.09	0.0381
**gamma_1b(variable)**	FEF2575	-0.06165	0.03896	194	-1.58	0.1152
**gamma_2b(variable)**	FVC	-0.00866	0.03759	186	-0.23	0.8181

	FEV1	-0.00641	0.02859	192	-0.22	0.8229
gamma_2b(variable)	PEFR	0.1233	0.1071	193	1.15	0.2511
gamma_2b(variable)	FEF2575	0.03622	0.04287	191	0.84	0.3993
gamma_3b(variable)	FVC	0.04221	0.03690	206	1.14	0.2540
gamma_3b(variable)	FEV1	0.07169	0.02897	218	2.47	0.0141
gamma_3b(variable)	PEFR	-0.1203	0.1077	223	-1.12	0.2652
gamma_3b(variable)	FEF2575	0.09345	0.04359	221	2.14	0.0331
gamma_4b(variable)	FVC	0.03305	0.03693	206	0.89	0.3720
gamma_4b(variable)	FEV1	-0.04730	0.02901	218	-1.63	0.1044
gamma_4b(variable)	PEFR	-0.06117	0.1078	223	-0.57	0.5711
gamma_4b(variable)	FEF2575	-0.1438	0.04364	221	-3.30	0.0011
gamma_5b(variable)	FVC	-0.1062	0.03824	186	-2.78	0.0060
gamma_5b(variable)	FEV1	-0.03286	0.02949	192	-1.11	0.2667
gamma_5b(variable)	PEFR	-0.3511	0.1095	192	-3.21	0.0016
gamma_5b(variable)	FEF2575	0.08730	0.04438	189	1.97	0.0506
gamma_2c(variable)	FVC	0.03698	0.03515	184	1.05	0.2941
gamma_2c(variable)	FEV1	0.01833	0.03452	178	0.53	0.5961
gamma_2c(variable)	PEFR	0.1617	0.1166	183	1.39	0.1672
gamma_2c(variable)	FEF2575	0.002675	0.05599	182	0.05	0.9619
gamma_3c(variable)	FVC	0.04975	0.03518	187	1.41	0.1590
gamma_3c(variable)	FEV1	0.08439	0.03490	179	2.42	0.0166
gamma_3c(variable)	PEFR	0.008774	0.1198	185	0.07	0.9417
gamma_3c(variable)	FEF2575	0.1607	0.05602	183	2.87	0.0046
gamma_4c(variable)	FVC	-0.02031	0.03523	187	-0.58	0.5650
gamma_4c(variable)	FEV1	-0.09801	0.03493	180	-2.81	0.0056
gamma_4c(variable)	PEFR	-0.2947	0.1199	186	-2.46	0.0149
gamma_4c(variable)	FEF2575	-0.2395	0.05607	184	-4.27	<.0001
gamma_5c(variable)	FVC	-0.05205	0.03504	199	-1.49	0.1390
gamma_5c(variable)	FEV1	-0.00954	0.03449	187	-0.28	0.7823
gamma_5c(variable)	PEFR	-0.1404	0.1178	195	-1.19	0.2350
gamma_5c(variable)	FEF2575	0.04170	0.05536	192	0.75	0.4523
center1(variable)	FVC	-0.1295	0.1054	186	-1.23	0.2209
center1(variable)	FEV1	-0.1783	0.09538	185	-1.87	0.0631
center1(variable)	PEFR	-0.3715	0.3223	186	-1.15	0.2506
center1(variable)	FEF2575	-0.2731	0.1402	186	-1.95	0.0529

	FVC	-0.1180	0.1037	186	-1.14	0.2566
**center2(variable)**	FEV1	-0.1476	0.09378	185	-1.57	0.1173
**center2(variable)**	PEFR	-0.3660	0.3169	186	-1.15	0.2496
**center2(variable)**	FEF2575	-0.2799	0.1378	186	-2.03	0.0437
**center3(variable)**	FVC	-0.1123	0.1051	186	-1.07	0.2868
**center3(variable)**	FEV1	-0.1878	0.09509	185	-1.97	0.0498
**center3(variable)**	PEFR	-0.5578	0.3213	186	-1.74	0.0842
**center3(variable)**	FEF2575	-0.3469	0.1398	186	-2.48	0.0139
**center5(variable)**	FVC	-0.1072	0.1061	186	-1.01	0.3135
**center5(variable)**	FEV1	-0.2306	0.09600	185	-2.40	0.0173
**center5(variable)**	PEFR	-0.4731	0.3244	186	-1.46	0.1464
**center5(variable)**	FEF2575	-0.4765	0.1411	186	-3.38	0.0009
**center6(variable)**	FVC	0.01703	0.1040	186	0.16	0.8701
**center6(variable)**	FEV1	-0.08045	0.09406	185	-0.86	0.3935
**center6(variable)**	PEFR	-0.4287	0.3178	186	-1.35	0.1790
**center6(variable)**	FEF2575	-0.2620	0.1382	186	-1.90	0.0596
**center7(variable)**	FVC	-0.07909	0.1121	186	-0.71	0.4815
**center7(variable)**	FEV1	-0.2037	0.1014	185	-2.01	0.0461
**center7(variable)**	PEFR	-0.3160	0.3428	186	-0.92	0.3577
**center7(variable)**	FEF2575	-0.4143	0.1491	186	-2.78	0.0060
**center8(variable)**	FVC	-0.09220	0.1025	186	-0.90	0.3697
**center8(variable)**	FEV1	-0.2427	0.09275	185	-2.62	0.0096
**center8(variable)**	PEFR	-0.5261	0.3134	186	-1.68	0.0950
**center8(variable)**	FEF2575	-0.4840	0.1363	186	-3.55	0.0005
**center9(variable)**	FVC	0.05668	0.1075	186	0.53	0.5986
**center9(variable)**	FEV1	-0.1266	0.09725	185	-1.30	0.1945
**center9(variable)**	PEFR	-0.4818	0.3286	186	-1.47	0.1443
**center9(variable)**	FEF2575	-0.4160	0.1429	186	-2.91	0.0041
**center10(variable)**	FVC	0.1506	0.1121	186	1.34	0.1808
**center10(variable)**	FEV1	0.08543	0.1015	185	0.84	0.4009
**center10(variable)**	PEFR	0.4283	0.3428	186	1.25	0.2131
**center10(variable)**	FEF2575	-0.04589	0.1491	186	-0.31	0.7586

Type 3 Tests of Fixed Effects

Effect	Num DF	Den DF	F Value	Pr > F

**mu_ac(variable)**	4	196	5.02	0.0007
**mu_b(variable)**	4	195	3.00	0.0196
**lambda(variable)**	4	375	6.61	<.0001
**gamma_1a(variable)**	4	215	1.25	0.2901
**gamma_2a(variable)**	4	208	0.65	0.6247
**gamma_3a(variable)**	4	186	2.51	0.0432
**gamma_4a(variable)**	4	186	2.72	0.0312
**gamma_5a(variable)**	4	185	0.72	0.5819
**gamma_1b(variable)**	4	194	6.28	<.0001
**gamma_2b(variable)**	4	190	2.70	0.0322
**gamma_3b(variable)**	4	220	4.04	0.0035
**gamma_4b(variable)**	4	220	3.35	0.0109
**gamma_5b(variable)**	4	190	4.13	0.0031
**gamma_2c(variable)**	4	186	0.88	0.4770
**gamma_3c(variable)**	4	189	2.75	0.0294
**gamma_4c(variable)**	4	189	5.23	0.0005
**gamma_5c(variable)**	4	200	1.61	0.1725
**center1(variable)**	4	187	1.13	0.3414
**center2(variable)**	4	187	1.66	0.1617
**center3(variable)**	4	187	2.07	0.0867
**center5(variable)**	4	187	3.21	0.0140
**center6(variable)**	4	187	1.74	0.1435
**center7(variable)**	4	187	1.97	0.1000
**center8(variable)**	4	187	3.40	0.0103
**center9(variable)**	4	187	3.30	0.0121
**center10(variable)**	4	187	0.81	0.5220

LR test statistic and p-value for H_0: mu_A=mu_C

Obs	Descr	reduced_model_likeli	full_model_likeli	lr_statistic	df	p_value
1	-2 Log Likelihood	-899.7	-914.9	15.2553	4	.004199924

Multivariate Analysis with Center as covariate - reduced model mu_B=mu_C

The Mixed Procedure

Model Information	
Data Set	WORK.TALC_3PERIOD_TEST_MEAN
Dependent Variable	outcome
Covariance Structure	Unstructured
Subject Effect	participant
Estimation Method	ML
Residual Variance Method	None
Fixed Effects SE Method	Model-Based
Degrees of Freedom Method	Satterthwaite

Class Level Information		
Class	Levels	Values
participant	187	1 2 3 5 7 8 10 11 12 13 14 15 16 17 18 19 20 21 22 23 24 25 26 27 28 29 30 31 32 33 35 36 37 38 39 41 42 43 44 45 47 48 50 52 53 54 55 56 57 58 59 60 61 62 63 65 66 67 68 69 70 71 72 73 74 75 76 77 78 79 80 81 82 83 84 85 86 87 88 91 92 93 94 95 96 97 98 99 100 101 102 103 104 105 106 108 109 110 111 113 114 116 117 118 119 120 121 122 123 124 125 126 127 128 129 130 131 132 133 134 135 136 137 139 140 141 142 143 144 145 146 147 148 149 150 151 152 153 154 155 156 157 158 159 160 161 163 164 165 167 168 170 171 173 174 175 176 179 180 181 182 183 184 185 186 187 188 189 190 191 192 193 194 196 197 198 199 200 201 202 203 204 206 207 208 209 210
variable	4	FVC FEV1 PEFR FEF2575
sequence	6	ABC ACB BAC BCA CAB CBA
period	3	1 2 3
treatment	3	Tiotropium Double-glucocorticoid Salmeterol

Dimensions	
Covariance Parameters	78
Columns in X	104
Columns in Z	0
Subjects	187
Max Obs per Subject	12

Number of Observations	
Number of Observations Read	2244
Number of Observations Used	2244

Number of Observations Not Used	0

Iteration History

Iteration	Evaluations	-2 Log Like	Criterion
0	1	2518.11722372	
1	2	-904.52477482	0.00002420
2	1	-904.58724250	0.00000005
3	1	-904.58735654	0.00000000

Convergence criteria met.

Covariance Parameter Estimates

Cov Parm	Subject	Estimate
UN(1,1)	participant	0.05756
UN(2,1)	participant	0.02166
UN(2,2)	participant	0.05409
UN(3,1)	participant	0.02635
UN(3,2)	participant	0.01646
UN(3,3)	participant	0.04774
UN(4,1)	participant	0.03857
UN(4,2)	participant	0.01437
UN(4,3)	participant	0.02252
UN(4,4)	participant	0.04490
UN(5,1)	participant	0.01682
UN(5,2)	participant	0.03006
UN(5,3)	participant	0.01529
UN(5,4)	participant	0.01775
UN(5,5)	participant	0.03083
UN(6,1)	participant	0.02021
UN(6,2)	participant	0.01407
UN(6,3)	participant	0.03679
UN(6,4)	participant	0.02531
UN(6,5)	participant	0.01913
UN(6,6)	participant	0.04701
UN(7,1)	participant	0.1152
UN(7,2)	participant	0.03212

UN(7,3)	participant	0.04074
UN(7,4)	participant	0.1020
UN(7,5)	participant	0.03023
UN(7,6)	participant	0.05117
UN(7,7)	participant	0.5956
UN(8,1)	participant	0.04257
UN(8,2)	participant	0.08467
UN(8,3)	participant	0.02156
UN(8,4)	participant	0.02519
UN(8,5)	participant	0.06115
UN(8,6)	participant	0.02602
UN(8,7)	participant	0.2278
UN(8,8)	participant	0.4342
UN(9,1)	participant	0.03089
UN(9,2)	participant	0.02755
UN(9,3)	participant	0.06317
UN(9,4)	participant	0.01801
UN(9,5)	participant	0.03665
UN(9,6)	participant	0.09624
UN(9,7)	participant	0.2055
UN(9,8)	participant	0.2042
UN(9,9)	participant	0.5532
UN(10,1)	participant	0.01574
UN(10,2)	participant	0.01367
UN(10,3)	participant	0.01568
UN(10,4)	participant	0.04933
UN(10,5)	participant	0.01886
UN(10,6)	participant	0.02760
UN(10,7)	participant	0.07737
UN(10,8)	participant	0.007477
UN(10,9)	participant	-0.00468
UN(10,10)	participant	0.1151
UN(11,1)	participant	0.006608
UN(11,2)	participant	0.000562
UN(11,3)	participant	0.01193

	participant	0.02059
UN(11,5)	participant	0.02842
UN(11,6)	participant	0.02170
UN(11,7)	participant	0.01268
UN(11,8)	participant	0.004908
UN(11,9)	participant	0.03489
UN(11,10)	participant	0.03256
UN(11,11)	participant	0.06970
UN(12,1)	participant	0.01468
UN(12,2)	participant	0.01510
UN(12,3)	participant	0.03049
UN(12,4)	participant	0.03378
UN(12,5)	participant	0.02583
UN(12,6)	participant	0.06281
UN(12,7)	participant	0.06316
UN(12,8)	participant	0.02014
UN(12,9)	participant	0.1278
UN(12,10)	participant	0.06452
UN(12,11)	participant	0.03887
UN(12,12)	participant	0.1246

Fit Statistics	
-2 Log Likelihood	-904.6
AIC (Smaller is Better)	-540.6
AICC (Smaller is Better)	-508.3
BIC (Smaller is Better)	47.5

Null Model Likelihood Ratio Test		
DF	Chi-Square	Pr > ChiSq
77	3422.70	<.0001

Solution for Fixed Effects						
Effect	variable	Estimate	Standard Error	DF	t Value	Pr > |t|
mu_a(variable)	FVC	0.2062	0.09974	196	2.07	0.0400
mu_a(variable)	FEV1	0.3196	0.08993	194	3.55	0.0005

**mu_a(variable)**	PEFR	0.9284	0.3063	197	3.03	0.0028
**mu_a(variable)**	FEF2575	0.5158	0.1328	197	3.88	0.0001
**mu_bc(variable)**	FVC	0.1428	0.09942	192	1.44	0.1526
**mu_bc(variable)**	FEV1	0.2261	0.08972	191	2.52	0.0126
**mu_bc(variable)**	PEFR	0.6129	0.3049	193	2.01	0.0458
**mu_bc(variable)**	FEF2575	0.3909	0.1322	192	2.96	0.0035
**lambda(variable)**	FVC	-0.05722	0.01646	400	-3.48	0.0006
**lambda(variable)**	FEV1	-0.02882	0.01374	372	-2.10	0.0366
**lambda(variable)**	PEFR	0.03669	0.05388	351	0.68	0.4964
**lambda(variable)**	FEF2575	-0.00695	0.02292	376	-0.30	0.7620
**gamma_1a(variable)**	FVC	-0.02121	0.03721	218	-0.57	0.5693
**gamma_1a(variable)**	FEV1	-0.01712	0.03263	234	-0.52	0.6003
**gamma_1a(variable)**	PEFR	0.1076	0.1180	213	0.91	0.3630
**gamma_1a(variable)**	FEF2575	-0.00482	0.05221	232	-0.09	0.9265
**gamma_2a(variable)**	FVC	0.01294	0.03711	208	0.35	0.7277
**gamma_2a(variable)**	FEV1	0.002079	0.03284	209	0.06	0.9496
**gamma_2a(variable)**	PEFR	0.1565	0.1189	208	1.32	0.1898
**gamma_2a(variable)**	FEF2575	-0.01909	0.05230	212	-0.37	0.7154
**gamma_3a(variable)**	FVC	0.05966	0.03965	186	1.50	0.1341
**gamma_3a(variable)**	FEV1	0.07466	0.03490	190	2.14	0.0337
**gamma_3a(variable)**	PEFR	-0.02930	0.1280	186	-0.23	0.8191
**gamma_3a(variable)**	FEF2575	0.1004	0.05551	191	1.81	0.0721
**gamma_4a(variable)**	FVC	0.01425	0.03966	186	0.36	0.7198
**gamma_4a(variable)**	FEV1	-0.04179	0.03490	190	-1.20	0.2327
**gamma_4a(variable)**	PEFR	-0.2785	0.1280	186	-2.18	0.0308
**gamma_4a(variable)**	FEF2575	-0.1284	0.05552	191	-2.31	0.0218
**gamma_5a(variable)**	FVC	-0.05280	0.03996	184	-1.32	0.1881
**gamma_5a(variable)**	FEV1	-0.02723	0.03548	189	-0.77	0.4436
**gamma_5a(variable)**	PEFR	-0.2102	0.1275	184	-1.65	0.1009
**gamma_5a(variable)**	FEF2575	0.009408	0.05703	189	0.16	0.8691
**gamma_1b(variable)**	FVC	-0.06430	0.03503	186	-1.84	0.0680
**gamma_1b(variable)**	FEV1	-0.01926	0.02579	194	-0.75	0.4560
**gamma_1b(variable)**	PEFR	-0.1570	0.09877	190	-1.59	0.1136
**gamma_1b(variable)**	FEF2575	-0.03666	0.03907	192	-0.94	0.3492
**gamma_2b(variable)**	FVC	-0.01729	0.03760	186	-0.46	0.6462

	FEV1	-0.01923	0.02856	192	-0.67	0.5015
gamma_2b(variable)	PEFR	0.08835	0.1073	191	0.82	0.4114
gamma_2b(variable)	FEF2575	0.01959	0.04284	191	0.46	0.6480
gamma_3b(variable)	FVC	0.04696	0.03688	206	1.27	0.2044
gamma_3b(variable)	FEV1	0.07891	0.02890	217	2.73	0.0069
gamma_3b(variable)	PEFR	-0.09450	0.1078	221	-0.88	0.3816
gamma_3b(variable)	FEF2575	0.1055	0.04355	222	2.42	0.0162
gamma_4b(variable)	FVC	0.03801	0.03692	206	1.03	0.3044
gamma_4b(variable)	FEV1	-0.03979	0.02894	217	-1.37	0.1706
gamma_4b(variable)	PEFR	-0.03443	0.1079	221	-0.32	0.7500
gamma_4b(variable)	FEF2575	-0.1314	0.04360	222	-3.01	0.0029
gamma_5b(variable)	FVC	-0.1146	0.03825	185	-3.00	0.0031
gamma_5b(variable)	FEV1	-0.04529	0.02947	192	-1.54	0.1259
gamma_5b(variable)	PEFR	-0.3839	0.1097	191	-3.50	0.0006
gamma_5b(variable)	FEF2575	0.07114	0.04437	190	1.60	0.1105
gamma_2c(variable)	FVC	0.03448	0.03506	185	0.98	0.3267
gamma_2c(variable)	FEV1	0.01388	0.03446	179	0.40	0.6875
gamma_2c(variable)	PEFR	0.1468	0.1170	183	1.25	0.2112
gamma_2c(variable)	FEF2575	-0.00385	0.05627	182	-0.07	0.9455
gamma_3c(variable)	FVC	0.04345	0.03507	188	1.24	0.2169
gamma_3c(variable)	FEV1	0.07418	0.03476	181	2.13	0.0342
gamma_3c(variable)	PEFR	-0.01959	0.1198	185	-0.16	0.8703
gamma_3c(variable)	FEF2575	0.1451	0.05624	183	2.58	0.0106
gamma_4c(variable)	FVC	-0.02639	0.03511	189	-0.75	0.4532
gamma_4c(variable)	FEV1	-0.1079	0.03480	182	-3.10	0.0022
gamma_4c(variable)	PEFR	-0.3221	0.1199	186	-2.69	0.0079
gamma_4c(variable)	FEF2575	-0.2547	0.05628	183	-4.53	<.0001
gamma_5c(variable)	FVC	-0.04385	0.03495	201	-1.25	0.2111
gamma_5c(variable)	FEV1	0.004012	0.03439	189	0.12	0.9072
gamma_5c(variable)	PEFR	-0.1003	0.1179	195	-0.85	0.3962
gamma_5c(variable)	FEF2575	0.06231	0.05559	191	1.12	0.2637
center1(variable)	FVC	-0.1251	0.1053	187	-1.19	0.2364
center1(variable)	FEV1	-0.1726	0.09519	187	-1.81	0.0714
center1(variable)	PEFR	-0.3535	0.3224	186	-1.10	0.2743
center1(variable)	FEF2575	-0.2663	0.1401	187	-1.90	0.0588

	FVC	-0.1130	0.1035	187	-1.09	0.2766
center2(variable)	FEV1	-0.1412	0.09359	187	-1.51	0.1331
center2(variable)	PEFR	-0.3469	0.3170	186	-1.09	0.2753
center2(variable)	FEF2575	-0.2724	0.1377	187	-1.98	0.0494
center3(variable)	FVC	-0.1076	0.1050	187	-1.02	0.3068
center3(variable)	FEV1	-0.1818	0.09490	187	-1.92	0.0570
center3(variable)	PEFR	-0.5398	0.3215	186	-1.68	0.0948
center3(variable)	FEF2575	-0.3398	0.1396	187	-2.43	0.0159
center5(variable)	FVC	-0.1039	0.1060	187	-0.98	0.3281
center5(variable)	FEV1	-0.2262	0.09580	187	-2.36	0.0192
center5(variable)	PEFR	-0.4595	0.3245	186	-1.42	0.1585
center5(variable)	FEF2575	-0.4714	0.1410	187	-3.34	0.0010
center6(variable)	FVC	0.02138	0.1038	187	0.21	0.8371
center6(variable)	FEV1	-0.07484	0.09387	187	-0.80	0.4263
center6(variable)	PEFR	-0.4113	0.3180	186	-1.29	0.1974
center6(variable)	FEF2575	-0.2554	0.1381	187	-1.85	0.0661
center7(variable)	FVC	-0.07491	0.1120	187	-0.67	0.5044
center7(variable)	FEV1	-0.1983	0.1012	187	-1.96	0.0516
center7(variable)	PEFR	-0.2996	0.3429	186	-0.87	0.3834
center7(variable)	FEF2575	-0.4079	0.1490	187	-2.74	0.0068
center8(variable)	FVC	-0.08702	0.1024	187	-0.85	0.3965
center8(variable)	FEV1	-0.2360	0.09257	187	-2.55	0.0116
center8(variable)	PEFR	-0.5054	0.3136	186	-1.61	0.1087
center8(variable)	FEF2575	-0.4761	0.1362	187	-3.50	0.0006
center9(variable)	FVC	0.06090	0.1074	187	0.57	0.5712
center9(variable)	FEV1	-0.1212	0.09705	187	-1.25	0.2132
center9(variable)	PEFR	-0.4657	0.3288	186	-1.42	0.1583
center9(variable)	FEF2575	-0.4096	0.1428	187	-2.87	0.0046
center10(variable)	FVC	0.1545	0.1120	187	1.38	0.1693
center10(variable)	FEV1	0.09054	0.1012	187	0.89	0.3723
center10(variable)	PEFR	0.4452	0.3430	186	1.30	0.1958
center10(variable)	FEF2575	-0.03971	0.1490	187	-0.27	0.7901

Type 3 Tests of Fixed Effects

Effect	Num DF	Den DF	F Value	Pr > F

**mu_a(variable)**	4	199	4.97	0.0008
**mu_bc(variable)**	4	193	2.76	0.0291
**lambda(variable)**	4	380	5.38	0.0003
**gamma_1a(variable)**	4	222	0.81	0.5189
**gamma_2a(variable)**	4	209	0.67	0.6167
**gamma_3a(variable)**	4	187	2.19	0.0712
**gamma_4a(variable)**	4	187	3.17	0.0149
**gamma_5a(variable)**	4	186	0.85	0.4929
**gamma_1b(variable)**	4	191	6.38	<.0001
**gamma_2b(variable)**	4	190	2.55	0.0407
**gamma_3b(variable)**	4	219	4.10	0.0032
**gamma_4b(variable)**	4	220	2.92	0.0222
**gamma_5b(variable)**	4	190	4.32	0.0023
**gamma_2c(variable)**	4	187	0.83	0.5070
**gamma_3c(variable)**	4	189	2.44	0.0487
**gamma_4c(variable)**	4	190	5.73	0.0002
**gamma_5c(variable)**	4	200	1.63	0.1680
**center1(variable)**	4	187	1.07	0.3705
**center2(variable)**	4	187	1.59	0.1776
**center3(variable)**	4	187	1.99	0.0981
**center5(variable)**	4	187	3.15	0.0156
**center6(variable)**	4	187	1.68	0.1554
**center7(variable)**	4	187	1.92	0.1093
**center8(variable)**	4	187	3.29	0.0124
**center9(variable)**	4	187	3.24	0.0134
**center10(variable)**	4	187	0.84	0.5041

LR test statistic and p-value for H_0: mu_B=mu_C

Obs	Descr	reduced_model_likeli	full_model_likeli	lr_statistic	df	p_value
1	-2 Log Likelihood	-904.6	-914.9	10.3228	4	0.035327

Multivariate Analysis with Center as covariate - reduced model mu_A=mu_B=mu_C

The Mixed Procedure

Model Information	
Data Set	WORK.TALC_3PERIOD_TEST_MEAN
Dependent Variable	outcome
Covariance Structure	Unstructured
Subject Effect	participant
Estimation Method	ML
Residual Variance Method	None
Fixed Effects SE Method	Model-Based
Degrees of Freedom Method	Satterthwaite

Class Level Information		
Class	Levels	Values
participant	187	1 2 3 5 7 8 10 11 12 13 14 15 16 17 18 19 20 21 22 23 24 25 26 27 28 29 30 31 32 33 35 36 37 38 39 41 42 43 44 45 47 48 50 52 53 54 55 56 57 58 59 60 61 62 63 65 66 67 68 69 70 71 72 73 74 75 76 77 78 79 80 81 82 83 84 85 86 87 88 91 92 93 94 95 96 97 98 99 100 101 102 103 104 105 106 108 109 110 111 113 114 116 117 118 119 120 121 122 123 124 125 126 127 128 129 130 131 132 133 134 135 136 137 139 140 141 142 143 144 145 146 147 148 149 150 151 152 153 154 155 156 157 158 159 160 161 163 164 165 167 168 170 171 173 174 175 176 179 180 181 182 183 184 185 186 187 188 189 190 191 192 193 194 196 197 198 199 200 201 202 203 204 206 207 208 209 210
variable	4	FVC FEV1 PEFR FEF2575
sequence	6	ABC ACB BAC BCA CAB CBA
period	3	1 2 3
treatment	3	Tiotropium Double-glucocorticoid Salmeterol

Dimensions	
Covariance Parameters	78
Columns in X	100
Columns in Z	0
Subjects	187
Max Obs per Subject	12

Number of Observations	
Number of Observations Read	2244
Number of Observations Used	2244

Number of Observations Not Used	0

Iteration History

Iteration	Evaluations	-2 Log Like	Criterion
0	1	2586.98597919	
1	2	-852.68016787	0.00036209
2	1	-853.67348429	0.00001186
3	1	-853.70371982	0.00000002
4	1	-853.70376287	0.00000000

Convergence criteria met.

Covariance Parameter Estimates

Cov Parm	Subject	Estimate
UN(1,1)	participant	0.05972
UN(2,1)	participant	0.02060
UN(2,2)	participant	0.05456
UN(3,1)	participant	0.02649
UN(3,2)	participant	0.01647
UN(3,3)	participant	0.04759
UN(4,1)	participant	0.04190
UN(4,2)	participant	0.01276
UN(4,3)	participant	0.02272
UN(4,4)	participant	0.05003
UN(5,1)	participant	0.01551
UN(5,2)	participant	0.03067
UN(5,3)	participant	0.01525
UN(5,4)	participant	0.01575
UN(5,5)	participant	0.03159
UN(6,1)	participant	0.02069
UN(6,2)	participant	0.01393
UN(6,3)	participant	0.03642
UN(6,4)	participant	0.02601
UN(6,5)	participant	0.01888
UN(6,6)	participant	0.04628
UN(7,1)	participant	0.1250

**UN(7,2)**	participant	0.02721
**UN(7,3)**	participant	0.04091
**UN(7,4)**	participant	0.1170
**UN(7,5)**	participant	0.02409
**UN(7,6)**	participant	0.05280
**UN(7,7)**	participant	0.6408
**UN(8,1)**	participant	0.03536
**UN(8,2)**	participant	0.08776
**UN(8,3)**	participant	0.02139
**UN(8,4)**	participant	0.01421
**UN(8,5)**	participant	0.06510
**UN(8,6)**	participant	0.02506
**UN(8,7)**	participant	0.1952
**UN(8,8)**	participant	0.4554
**UN(9,1)**	participant	0.03086
**UN(9,2)**	participant	0.02769
**UN(9,3)**	participant	0.06106
**UN(9,4)**	participant	0.01778
**UN(9,5)**	participant	0.03643
**UN(9,6)**	participant	0.09245
**UN(9,7)**	participant	0.2048
**UN(9,8)**	participant	0.2066
**UN(9,9)**	participant	0.5395
**UN(10,1)**	participant	0.02103
**UN(10,2)**	participant	0.01113
**UN(10,3)**	participant	0.01592
**UN(10,4)**	participant	0.05746
**UN(10,5)**	participant	0.01569
**UN(10,6)**	participant	0.02861
**UN(10,7)**	participant	0.1014
**UN(10,8)**	participant	-0.00977
**UN(10,9)**	participant	-0.00526
**UN(10,10)**	participant	0.1280
**UN(11,1)**	participant	0.005728
**UN(11,2)**	participant	0.000976

	participant	0.01188
**UN(11,4)**	participant	0.01924
**UN(11,5)**	participant	0.02894
**UN(11,6)**	participant	0.02146
**UN(11,7)**	participant	0.008424
**UN(11,8)**	participant	0.007561
**UN(11,9)**	participant	0.03432
**UN(11,10)**	participant	0.03042
**UN(11,11)**	participant	0.07006
**UN(12,1)**	participant	0.01660
**UN(12,2)**	participant	0.01433
**UN(12,3)**	participant	0.02983
**UN(12,4)**	participant	0.03664
**UN(12,5)**	participant	0.02471
**UN(12,6)**	participant	0.06168
**UN(12,7)**	participant	0.07111
**UN(12,8)**	participant	0.01510
**UN(12,9)**	participant	0.1213
**UN(12,10)**	participant	0.06890
**UN(12,11)**	participant	0.03797
**UN(12,12)**	participant	0.1235

**Fit Statistics**	
**-2 Log Likelihood**	-853.7
**AIC (Smaller is Better)**	-497.7
**AICC (Smaller is Better)**	-466.8
**BIC (Smaller is Better)**	77.4

Null Model Likelihood Ratio Test

**DF**	**Chi-Square**	**Pr > ChiSq**
77	3440.69	<.0001

Solution for Fixed Effects

**Effect**	**variable**	**Estimate**	**Standard Error**	**DF**	**t Value**	**Pr > |t|**
**mu_abc(variable)**	FVC	0.1684	0.09928	192	1.70	0.0914

**mu_abc(variable)**	FEV1	0.2617	0.08960	191	2.92	0.0039
**mu_abc(variable)**	PEFR	0.7424	0.3042	193	2.44	0.0156
**mu_abc(variable)**	FEF2575	0.4249	0.1320	192	3.22	0.0015
**lambda(variable)**	FVC	-0.06572	0.01660	397	-3.96	<.0001
**lambda(variable)**	FEV1	-0.04008	0.01414	379	-2.83	0.0048
**lambda(variable)**	PEFR	0.01537	0.05525	359	0.28	0.7810
**lambda(variable)**	FEF2575	-0.02295	0.02317	385	-0.99	0.3225
**gamma_1a(variable)**	FVC	-0.04014	0.03745	213	-1.07	0.2850
**gamma_1a(variable)**	FEV1	-0.04356	0.03361	214	-1.30	0.1963
**gamma_1a(variable)**	PEFR	0.08032	0.1210	198	0.66	0.5077
**gamma_1a(variable)**	FEF2575	-0.03775	0.05376	218	-0.70	0.4833
**gamma_2a(variable)**	FVC	0.01135	0.03767	199	0.30	0.7634
**gamma_2a(variable)**	FEV1	0.000604	0.03432	185	0.02	0.9860
**gamma_2a(variable)**	PEFR	0.1540	0.1224	192	1.26	0.2099
**gamma_2a(variable)**	FEF2575	-0.02164	0.05471	188	-0.40	0.6929
**gamma_3a(variable)**	FVC	0.06553	0.04029	177	1.63	0.1057
**gamma_3a(variable)**	FEV1	0.08283	0.03663	168	2.26	0.0250
**gamma_3a(variable)**	PEFR	-0.01841	0.1321	172	-0.14	0.8893
**gamma_3a(variable)**	FEF2575	0.1110	0.05828	171	1.90	0.0585
**gamma_4a(variable)**	FVC	0.01994	0.04030	177	0.49	0.6214
**gamma_4a(variable)**	FEV1	-0.03386	0.03664	168	-0.92	0.3568
**gamma_4a(variable)**	PEFR	-0.2680	0.1321	172	-2.03	0.0440
**gamma_4a(variable)**	FEF2575	-0.1181	0.05830	171	-2.03	0.0443
**gamma_5a(variable)**	FVC	-0.04764	0.04068	175	-1.17	0.2432
**gamma_5a(variable)**	FEV1	-0.02028	0.03739	166	-0.54	0.5882
**gamma_5a(variable)**	PEFR	-0.2027	0.1323	170	-1.53	0.1274
**gamma_5a(variable)**	FEF2575	0.01834	0.06005	170	0.31	0.7604
**gamma_1b(variable)**	FVC	-0.07644	0.03507	186	-2.18	0.0306
**gamma_1b(variable)**	FEV1	-0.03680	0.02592	190	-1.42	0.1573
**gamma_1b(variable)**	PEFR	-0.1732	0.1003	182	-1.73	0.0861
**gamma_1b(variable)**	FEF2575	-0.05606	0.03901	193	-1.44	0.1523
**gamma_2b(variable)**	FVC	-0.01216	0.03769	184	-0.32	0.7474
**gamma_2b(variable)**	FEV1	-0.01225	0.02878	186	-0.43	0.6709
**gamma_2b(variable)**	PEFR	0.09438	0.1092	180	0.86	0.3886
**gamma_2b(variable)**	FEF2575	0.02915	0.04287	190	0.68	0.4973

	FVC	0.04386	0.03700	204	1.19	0.2372
**gamma_3b(variable)**	FEV1	0.07523	0.02915	211	2.58	0.0105
**gamma_3b(variable)**	PEFR	-0.1041	0.1097	207	-0.95	0.3437
**gamma_3b(variable)**	FEF2575	0.09916	0.04360	222	2.27	0.0239
**gamma_4b(variable)**	FVC	0.03474	0.03704	204	0.94	0.3493
**gamma_4b(variable)**	FEV1	-0.04370	0.02918	211	-1.50	0.1358
**gamma_4b(variable)**	PEFR	-0.04437	0.1098	207	-0.40	0.6865
**gamma_4b(variable)**	FEF2575	-0.1381	0.04365	222	-3.16	0.0018
**gamma_5b(variable)**	FVC	-0.1084	0.03842	183	-2.82	0.0053
**gamma_5b(variable)**	FEV1	-0.03681	0.02983	183	-1.23	0.2188
**gamma_5b(variable)**	PEFR	-0.3712	0.1124	176	-3.30	0.0012
**gamma_5b(variable)**	FEF2575	0.08202	0.04446	188	1.84	0.0667
**gamma_2c(variable)**	FVC	0.03728	0.03504	185	1.06	0.2887
**gamma_2c(variable)**	FEV1	0.01778	0.03430	180	0.52	0.6049
**gamma_2c(variable)**	PEFR	0.1527	0.1157	188	1.32	0.1886
**gamma_2c(variable)**	FEF2575	0.002568	0.05627	179	0.05	0.9636
**gamma_3c(variable)**	FVC	0.04808	0.03501	190	1.37	0.1713
**gamma_3c(variable)**	FEV1	0.08027	0.03450	185	2.33	0.0211
**gamma_3c(variable)**	PEFR	-0.00889	0.1186	190	-0.07	0.9403
**gamma_3c(variable)**	FEF2575	0.1536	0.05593	184	2.75	0.0066
**gamma_4c(variable)**	FVC	-0.02193	0.03506	190	-0.63	0.5323
**gamma_4c(variable)**	FEV1	-0.1021	0.03454	185	-2.96	0.0035
**gamma_4c(variable)**	PEFR	-0.3118	0.1187	191	-2.63	0.0093
**gamma_4c(variable)**	FEF2575	-0.2465	0.05598	184	-4.40	<.0001
**gamma_5c(variable)**	FVC	-0.05014	0.03492	202	-1.44	0.1526
**gamma_5c(variable)**	FEV1	-0.00440	0.03422	193	-0.13	0.8978
**gamma_5c(variable)**	PEFR	-0.1158	0.1170	203	-0.99	0.3234
**gamma_5c(variable)**	FEF2575	0.05030	0.05544	192	0.91	0.3654
**center1(variable)**	FVC	-0.1285	0.1053	187	-1.22	0.2240
**center1(variable)**	FEV1	-0.1770	0.09517	187	-1.86	0.0644
**center1(variable)**	PEFR	-0.3593	0.3222	187	-1.12	0.2662
**center1(variable)**	FEF2575	-0.2721	0.1400	187	-1.94	0.0535
**center2(variable)**	FVC	-0.1165	0.1035	187	-1.13	0.2620
**center2(variable)**	FEV1	-0.1458	0.09357	187	-1.56	0.1210
**center2(variable)**	PEFR	-0.3519	0.3168	187	-1.11	0.2681

	FEF2575	-0.2783	0.1377	187	-2.02	0.0447
**center3(variable)**	FVC	-0.1109	0.1050	187	-1.06	0.2922
**center3(variable)**	FEV1	-0.1861	0.09488	187	-1.96	0.0514
**center3(variable)**	PEFR	-0.5444	0.3212	187	-1.69	0.0918
**center3(variable)**	FEF2575	-0.3454	0.1396	187	-2.47	0.0143
**center5(variable)**	FVC	-0.1065	0.1060	187	-1.01	0.3162
**center5(variable)**	FEV1	-0.2297	0.09579	187	-2.40	0.0175
**center5(variable)**	PEFR	-0.4639	0.3243	187	-1.43	0.1543
**center5(variable)**	FEF2575	-0.4758	0.1409	187	-3.38	0.0009
**center6(variable)**	FVC	0.01794	0.1038	187	0.17	0.8630
**center6(variable)**	FEV1	-0.07932	0.09385	187	-0.85	0.3991
**center6(variable)**	PEFR	-0.4161	0.3177	187	-1.31	0.1919
**center6(variable)**	FEF2575	-0.2611	0.1381	187	-1.89	0.0602
**center7(variable)**	FVC	-0.07793	0.1120	187	-0.70	0.4874
**center7(variable)**	FEV1	-0.2023	0.1012	187	-2.00	0.0471
**center7(variable)**	PEFR	-0.3046	0.3427	187	-0.89	0.3753
**center7(variable)**	FEF2575	-0.4130	0.1489	187	-2.77	0.0061
**center8(variable)**	FVC	-0.09091	0.1024	187	-0.89	0.3758
**center8(variable)**	FEV1	-0.2411	0.09255	187	-2.60	0.0099
**center8(variable)**	PEFR	-0.5117	0.3133	187	-1.63	0.1041
**center8(variable)**	FEF2575	-0.4827	0.1362	187	-3.54	0.0005
**center9(variable)**	FVC	0.05793	0.1074	187	0.54	0.5901
**center9(variable)**	FEV1	-0.1251	0.09704	187	-1.29	0.1989
**center9(variable)**	PEFR	-0.4698	0.3285	187	-1.43	0.1543
**center9(variable)**	FEF2575	-0.4146	0.1428	187	-2.90	0.0041
**center10(variable)**	FVC	0.1512	0.1120	187	1.35	0.1787
**center10(variable)**	FEV1	0.08612	0.1012	187	0.85	0.3960
**center10(variable)**	PEFR	0.4390	0.3427	187	1.28	0.2018
**center10(variable)**	FEF2575	-0.04546	0.1490	187	-0.31	0.7606

Type 3 Tests of Fixed Effects

Effect	Num DF	Den DF	F Value	Pr > F
**mu_abc(variable)**	4	192	3.37	0.0109
**lambda(variable)**	4	381	6.72	<.0001
**gamma_1a(variable)**	4	218	1.48	0.2103

**gamma_2a(variable)**	4	200	0.67	0.6109
**gamma_3a(variable)**	4	180	2.45	0.0477
**gamma_4a(variable)**	4	180	2.92	0.0228
**gamma_5a(variable)**	4	179	0.84	0.4998
**gamma_1b(variable)**	4	190	6.05	0.0001
**gamma_2b(variable)**	4	187	2.50	0.0444
**gamma_3b(variable)**	4	215	4.03	0.0036
**gamma_4b(variable)**	4	215	3.09	0.0169
**gamma_5b(variable)**	4	185	4.19	0.0029
**gamma_2c(variable)**	4	186	0.84	0.4985
**gamma_3c(variable)**	4	188	2.64	0.0355
**gamma_4c(variable)**	4	189	5.53	0.0003
**gamma_5c(variable)**	4	200	1.59	0.1772
**center1(variable)**	4	187	1.12	0.3477
**center2(variable)**	4	187	1.64	0.1668
**center3(variable)**	4	187	2.04	0.0909
**center5(variable)**	4	187	3.20	0.0143
**center6(variable)**	4	187	1.71	0.1501
**center7(variable)**	4	187	1.96	0.1017
**center8(variable)**	4	187	3.37	0.0108
**center9(variable)**	4	187	3.27	0.0127
**center10(variable)**	4	187	0.82	0.5112

LR test statistic and p-value for H_0: mu_A=mu_B=mu_C

Obs	Descr	reduced_model_likeli	full_model_likeli	lr_statistic	df	p_value
1	-2 Log Likelihood	-853.7	-914.9	61.2064	8	2.7013E-10
