# Supplementary material for: Multivariate Analysis of Data From Crossover Clinical Trials
Source: Biom J. 2026 Oct 11;68(5):e70193. doi: 10.1002/bimj.70193 (PMC13655226; doi:10.1002/bimj.70193)
Supplement: Supplementary file 1 [file BIMJ-68-e70193-s001.zip › code_and_data_supplement/readme.html]

Aim 1 code and data supplement — README


# README

## R environment

- R version: 4.1.1
- Platform: x86\_64-w64-mingw32/x64 (64-bit)
- Main packages: `MASS` (7.3-54) and `dplyr` (1.0.7)

## Repository tree

- `README.md` and `readme.html` — documentation files.
- `Data_Application/`
  - `pseudo_TALC3period.csv` — pseudo TALC data used for the application analysis.
  - `talc3period_analysis.sas` — SAS application analysis.
  - `SAS_output_results.pdf` — saved SAS output from the pseudo-data application analysis, provided for the reproducibility audit.
- `Simulation/`
  - `fixed_effects_3period.csv` — REML estimates from the original data, used to generate simulation data sets.
  - `Rmatrix_3period.csv` — REML covariance-matrix estimates from the original data, used to generate simulation data sets.
  - `pseudo_TALC3period.csv` — pseudo data resembling the original data, used to generate simulation data sets.
  - `generated/scenario{1,2,3}/` — 45 saved R-generated simulation data sets (15 per scenario).
  - `results/r/generation_tasks/` — one compact manifest per generated data set, recording the scenario, data-set identifier, seed, output filename, and row count.
  - `Scenario1/` — corresponds to results in Table 1; contains the R generator, PowerShell launcher, and SAS analysis.
  - `Scenario2/` — same structure as Scenario1; corresponds to Table 2.
  - `Scenario3/` — same structure as Scenario1; corresponds to Table 3.

The supplied CSV inputs are pseudo data rather than the original trial data. Therefore, the application-analysis results are not expected to be identical to those reported in the manuscript.

## Reproduction guide

Prerequisites: R (4.1.1), SAS (9.4), PowerShell, and `Rscript` available on the system `PATH`.

All R paths are determined from the location of the R script. Each SAS program sets its working directory to the folder containing the current program file and then uses relative paths. Thus the complete supplement can be moved as one folder without editing source-code paths. If `SAS_EXECFILEPATH` and `SAS_EXECFILENAME` are unavailable in a particular SAS environment, manually set SAS's current working directory to the folder containing the program before submitting it.

1. **Regenerate simulation data sets.**
   - Run each PowerShell launcher from the supplement root. Each launcher runs the R generator for all 15 archived seeds; each generated data set contains 1,000 sub-datasets.

   ```
   .\Simulation\Scenario1\run_generation.ps1
   .\Simulation\Scenario2\run_generation.ps1
   .\Simulation\Scenario3\run_generation.ps1
   ```

   - Generated CSV files are saved as `Simulation/generated/scenarioN/simu_data_3period_sN(<dataset>).csv`. Task manifests are saved in `Simulation/results/r/generation_tasks/`.
   - To regenerate one Scenario 1 data set, run the following from `Simulation/Scenario1/`:

   ```
   Rscript .\data_generation_3period_s1_auto.R --dataset=1 --seed=952163715
   ```
2. **Run simulation analyses.**
   - For each scenario, run `TALC_3period_simulation_lrt_auto.sas` in the respective `Simulation/ScenarioN/` folder. It reads `../generated/scenarioN/simu_data_3period_sN(<dataset>).csv`.
   - Set the data-set number near the start of the SAS program:

   ```
   %let DATASET = 15;
   ```

   Replace `15` with an integer from 1 through 15. No other code edits are required. The output is statistical power from the multivariate and univariate approaches, printed by `PROC MEANS`.
3. **Run the application analysis.**
   - Run `Data_Application/talc3period_analysis.sas`. It reads `pseudo_TALC3period.csv` from the same folder.
   - `Data_Application/SAS_output_results.pdf` is the saved output from this analysis on the supplied pseudo data. It provides the compact intermediate tables and test statistics for the audit; a reviewer can compare their re-run with this PDF.

## Outputs

The SAS simulation programs produce the power summaries used for Tables 1–3. The saved generated data sets and their manifests support spot checks of individual scenario/data-set combinations. `SAS_output_results.pdf` is the supplied intermediate output for the pseudo-data application analysis.
